# Ground- and excited-state dynamic control of an anion receptor by hydrostatic pressure†

**DOI:** 10.1039/d1sc00664a

**Published:** 2021-04-15

**Authors:** Tomokazu Kinoshita, Yohei Haketa, Hiromitsu Maeda, Gaku Fukuhara

**Affiliations:** Department of Chemistry, Tokyo Institute of Technology 2-12-1 Ookayama, Meguro-ku Tokyo 152-8551 Japan gaku@chem.titech.ac.jp; Department of Applied Chemistry, College of Life Sciences, Ritsumeikan University Kusatsu 525-8577 Japan maedahir@ph.ritsumei.ac.jp; JST, PRESTO 4-1-8 Honcho, Kawaguchi Saitama 332-0012 Japan

## Abstract

Stimulus-responsive supramolecular architectures have become an attractive alternative to conventional ones for many applications in sensing, drug-delivery and switchable memory systems. Herein, we used an anion receptor (**H**: host) as a hydrostatic-pressure-manipulatable fluorescence foldamer and halide anions as chiral (binaphthylammonium) and achiral (tetrabutylammonium) ion pairs (**SS** or **RR***·*X and TBA·X; X = Cl, Br), and then investigated their (chir)optical properties and molecular recognition behavior under hydrostatic pressures. The conformational changes and optical properties of **H** in various organic solvents were revealed by UV/vis absorption and fluorescence spectra and fluorescence lifetimes upon hydrostatic pressurization. The anion-recognition abilities of **H** upon interactions with **SS** or **RR**·X and TBA·X at different pressure ranges were determined by hydrostatic-pressure spectroscopy to quantitatively afford the binding constant (*K*_anion_) and apparent reaction volume changes 
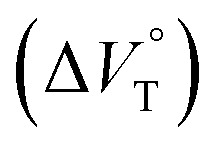
. The results obtained indicate that hydrostatic pressure as well as solvation plays significant roles in the dynamic control of the present supramolecular system in the ground and excited states. This work will provide a new guideline for further developing hydrostatic-pressure-responsive foldamers and supramolecular materials.

## Abstract 2

Hydrostatic pressure can control interactions of chiral countercations with helical receptors containing anions, causing remarkable chiroptical changes.

## Graphical abstract

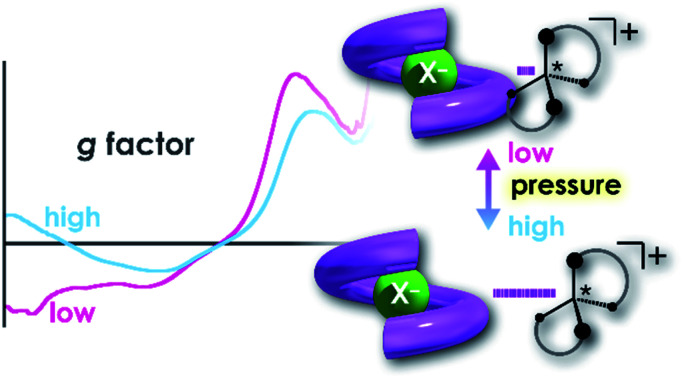

## Introduction

A large number of chemosensors, pre-organized hosts and supramolecular assemblies/polymers have been exploited in a rather long history since the great discovery of crown ethers in 1967.^1–3^ Today, sophisticated supramolecular materials triggered by a wide variety of external stimuli, *e.g*., temperature, solvent, pH or electronic excitation, have been developed to achieve “molecular machines” that can alter the molecular-level structure/function/properties.^4–6^ Even apart from such artificial molecular machines, the creation of stimulus-responsive supramolecular architectures is a recent great trend for some applications in chemical and apoptosis sensing,^7–12^ drug-delivery^13–15^ and switchable memory systems.^16–19^

Hydrostatic pressure, one of the mechanical stimuli, has attracted attention for a long time since the early 1960s,^20–27^ since hydrostatic pressurization of object solutions can control not only ground-state thermodynamic equilibria in molecular recognition^28–35^ and biomolecular events^36–38^ but also excited-state kinetic rates in photophysics and photochemical reactions.^39–42^ Despite being quite an old topic, this area has come into the limelight again from the viewpoints of mechanochromic chemistry^43–49^ and mechanobiology^37,50–52^ as relatively new scientific fields, and thus presents a major challenge in current chemistry. Indeed, we have recently revealed that optical properties, molecular and biomolecular recognition behavior and photo-physical/chemical processes in solutions of various molecular, supramolecular, macromolecular and biomacromolecular systems are precisely regulated by hydrostatic pressure.^53–63^ Hence, these trends prompted us to newly investigate an applicable supramolecular recognition system under hydrostatic pressures.

To this end, we focus on foldamers as artificial receptors or chemosensors which are a type of synthetic oligomers that show dynamic folded and unfolded states in solutions.^64^ Foldamers consist of rather flexible strands and form well-organized supramolecular structures with neutral and/or anionic guest molecules, and thus may be considered as an induced-fit type receptor.^65–67^ In fact, Moore and Drickamer *et al.* demonstrated that binding constants (*K*) of the piperazine or terpene guest upon interactions with oligo (*m*-phenylene ethynylene) foldamers in polymer matrixes decreased with increasing pressure of up to 65 000 MPa, *i.e*., positive reaction volumes (Δ*V*° > 0), revealed by the diamond anvil cell technique.^68^ Nevertheless, solution-state molecular recognition behavior of foldamers under hydrostatic pressures has not been examined in any detail, and hence, is still a challenge for the further evolution of supramolecular chemistry.

**Fig. 1:**
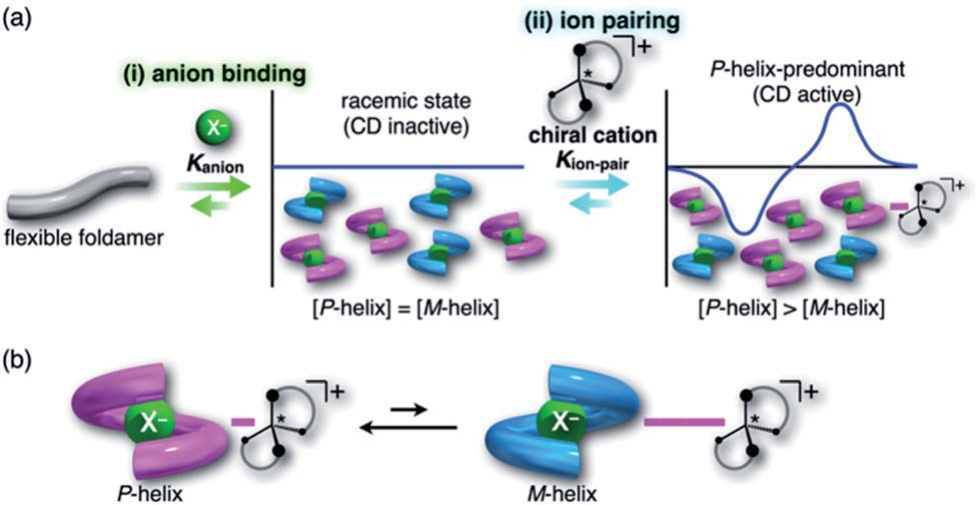
(a) Conceptual diagram for (i) anion-binding and (ii) ion-pairing chirality induction behaviors of a flexible anion-responsive foldamer with the chiral countercation and (b) equilibrium between two ion pair species comprising an *M*-helix foldamer with a tightly associated chiral countercation and a *P*-helix foldamer with a weakly associated chiral countercation. In (a), *K*_anion_ is defined as the binding constant of the receptor and anion and *K*_ion-pair_ is defined as the association constant of the receptor–anion complex and countercation.

For foldamers (receptors) that show helical structures upon binding anions as a guest species,^67^ chirality of the helical anion complexes can be predominantly regulated by proximally locating the chiral countercation (Fig. 1a). The chirality regulation can be achieved by two crucial processes: (i) anion binding of the receptor and (ii) ion pairing with the chiral countercation. In the anion-binding step (i), anion complexes exist as equimolar amounts of *M*- and *P*-helices, resulting in the achiral racemic state without circular dichroism (CD) signs. In the ion-pairing step (ii), either of the helicities is predominantly formed upon ion-pair formation with the chiral countercation, inducing the asymmetric state with enhanced CD signs. The diastereoselective formation of helical structures is achieved by the equilibrium between ion pairs with *M*- and *P*-helical structures that show different stabilities (Fig. 1b). Such a multi-step chirality induction system can be easily regulated because the chirality induction of the foldamer is a result of the ion pair formation, which is sensitive to the solvation conditions. Therefore, the helical structures are tunable by external pressure, which influences solvation conditions.

**Fig. 2:**
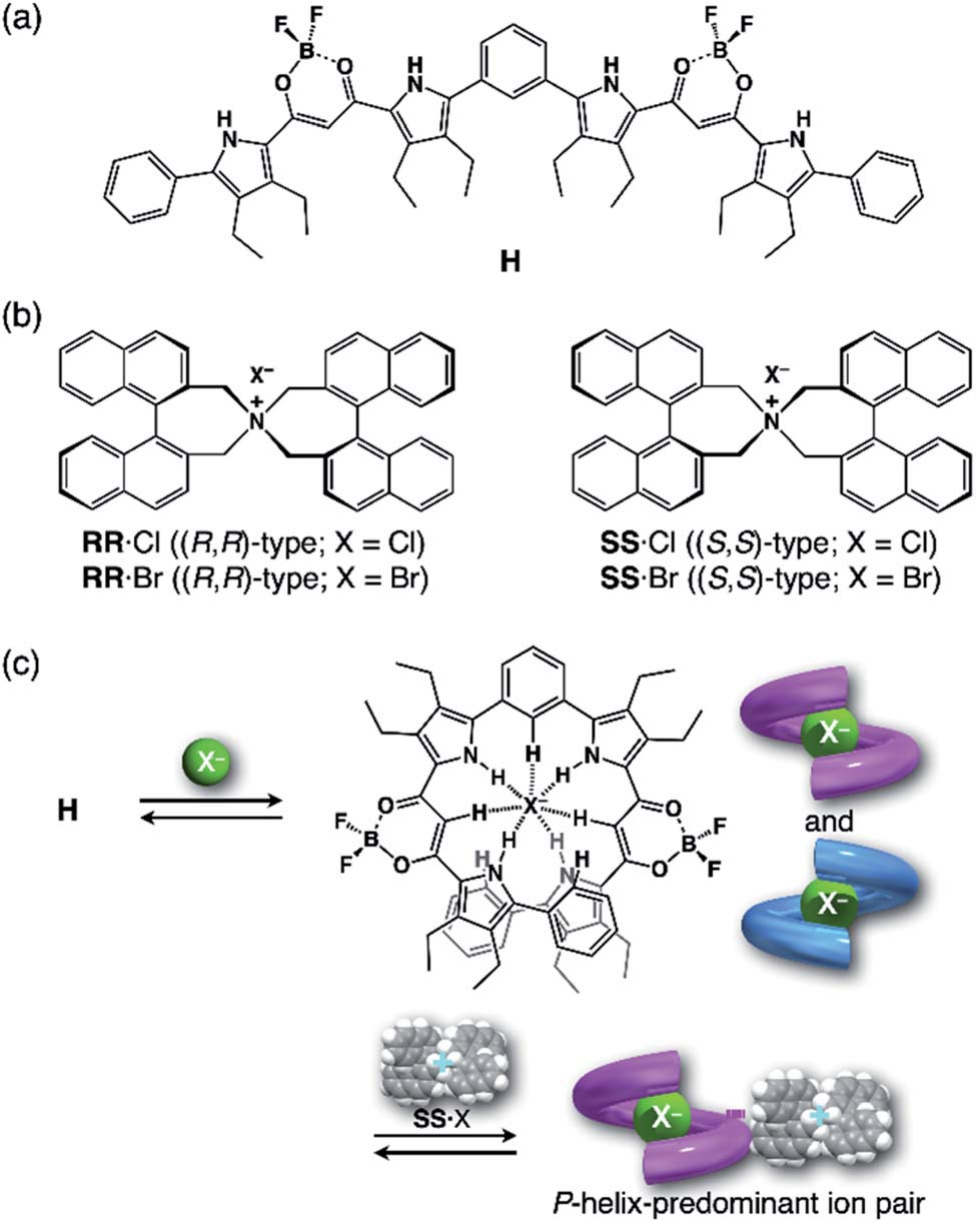
(a) Fluorescence anion receptor (**H**: host), (b) chiral ion pairs (**SS** or **RR**·X; X = Cl, Br) and (c) their supramolecular complexation mode.

In the present study, to thus apply the hydrostatic pressure-control concept to chemical sensing of foldamers in solutions, we chose a combination of an anion receptor (**H**: host) as a fluorescence foldamer and chiral ion pairs (**SS** or **RR**·X) as guests, as shown in Fig. 2a and b, and investigated their (chir)optical properties and molecular recognition behavior upon hydrostatic pressurization. This host–guest combination seems to be rather reasonable for achieving the present purpose, since we have clearly revealed the molecular recognitions at an atmospheric condition (0.1 MPa) (Fig. 2c).^69,70^ Also, among many outstanding anion receptors reported so far,^71–75^ both of them used here were spectroscopically powerful due to their colorimetric, fluorometric and optical properties. Herein, we wish to report an unprecedented dynamic supramolecular control of **H** in both ground and excited states, induced by hydrostatic pressure. The results obtained here and the concepts and methodology proposed in this paper provide deeper insights for constructing further smart and dynamic supramolecular architectures.

## Results and discussion

### Dynamic behavior of the fluorescence anion receptor (**H**) upon hydrostatic pressurization

**Fig. 3:**
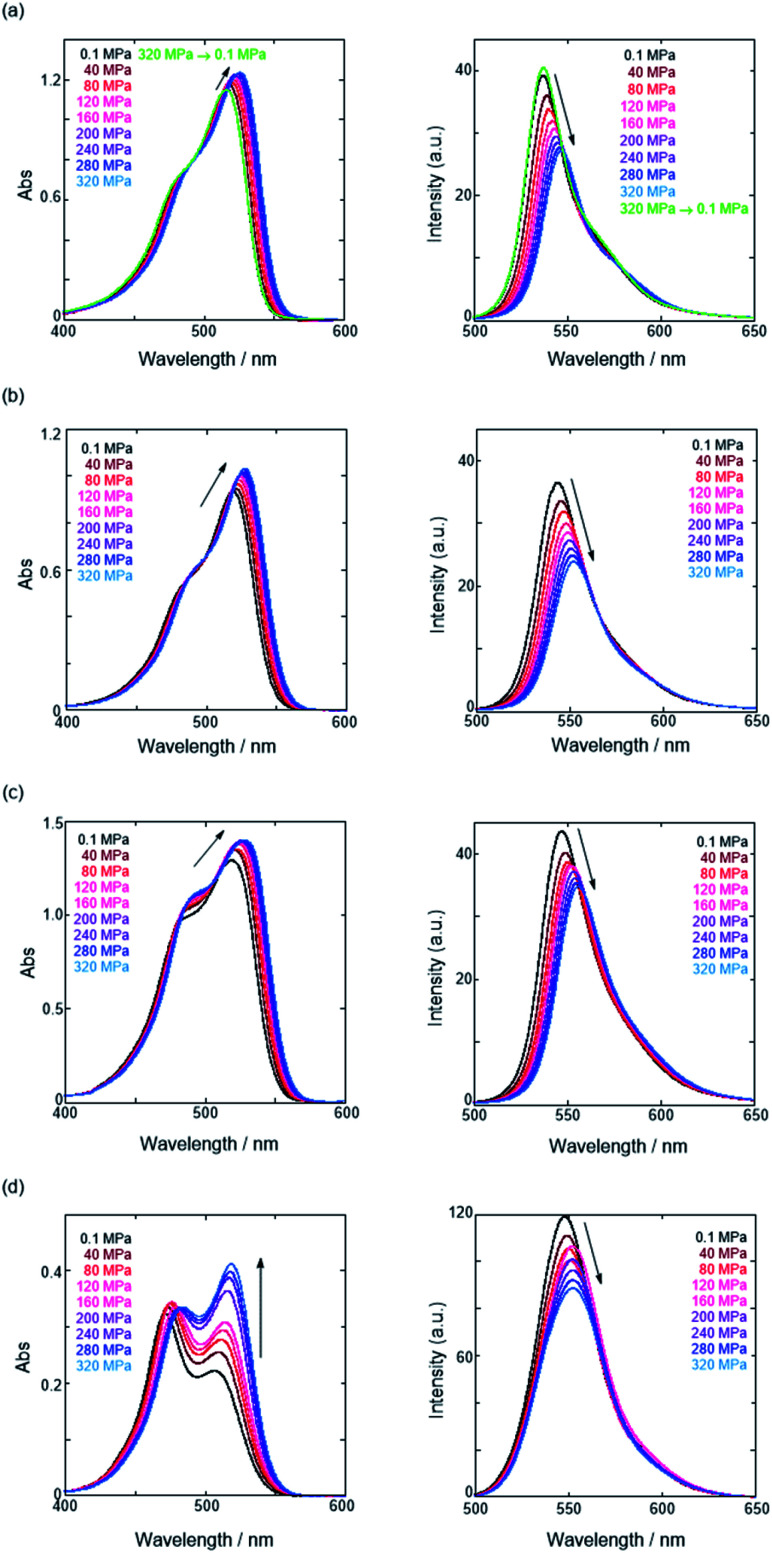
UV/vis absorption (*left panels*) and fluorescence spectra (*right panels*, *λ*_ex_ 404 nm) of **H** at 0.1, 40, 80, 120, 160, 200, 240, 280 and 320 MPa (from black to sky blue lines) in (a) toluene (44 μM), (b) chloroform (42 μM), (c) dichloromethane (49 μM) and (d) acetonitrile (15 μM) at room temperature, measured in a high-pressure cell. The green line shows the spectrum at 0.1 MPa, depressurized from 320 MPa. The absorbances at the excitation wavelength of 404 nm were identical.

First, we investigated UV/vis absorption and fluorescence spectral changes of the anion receptor (**H**) in various organic solvents under hydrostatic pressures (for the detailed hydrostatic-pressure apparatus, see Fig. S1 in the ESI†). To clearly discuss microenvironmental polarity changes around **H** under hydrostatic pressures, in this paper, we used the *E*_T_ parameter^76^ based on the pressure effects of solvatochromic Reichardt's dye.^58^ Solutions of **H** were prepared by dissolving in toluene, chloroform, dichloromethane and acetonitrile (*E*_T_: 33.9–45.6 kcal mol^−1^), and then subjected to hydrostatic-pressurized UV/vis absorption (Fig. 3, *left panels*), fluorescence (Fig. 3, *right panels*) and excitation spectroscopies (Fig. S2 in the ESI†). Each solution was adjusted to an appropriate concentration of <50 μM for avoiding high-pressure crystallization.

**Fig. 4:**
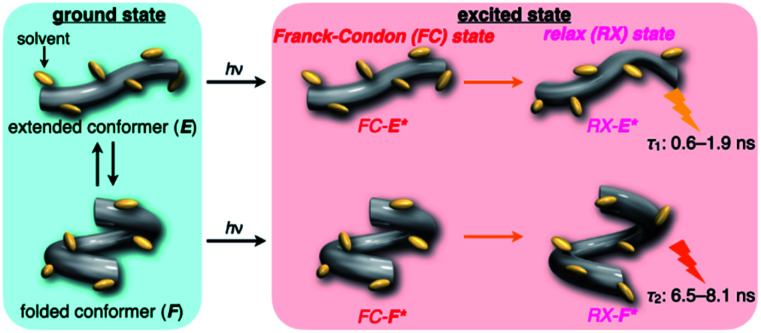
Schematic illustration of the dynamic behavior of **H** in the ground and excited states.

The spectra at 0.1 MPa (green line) depressurized from 320 MPa are almost superimposable with the original spectra (black line), indicating a reversible process upon pressurization. All the UV/vis absorption spectra (Fig. 3, *left*) exhibited gradual bathochromic shifts and hyperchromic effects with increasing pressure, of which the former were plotted against pressure to quantitatively afford a spectral shift per unit pressure for the 0–0 absorption band (*α*_A_) listed in Table 1; see Fig. S3 in the ESI† for the plots. As the former reason, it is well-known that solvent density changes upon hydrostatic pressurization perturbate orbitals in π systems to cause pressure-induced red shifts.^20,21^ The latter are simply due to the increase in the effective concentration upon pressurization.^21^ Since the hydrostatic pressure effect on the red shift is generally observed as *ca.* 1 cm^−1^ MPa^−1^ in using common organic dyes,^54–63^ the *α*_A_ values obtained in toluene, chloroform and dichloromethane are reasonably pronounced as the general hydrostatic-pressure-induced spectral change, which means that a particular conformational change in the foldamer skeleton does not occur upon pressurization. Very interestingly, only in acetonitrile, the 0–0 absorption band is quite suppressed under low pressures, compared to the 0–1 band. According to the previous mechanistic studies,^69,70^ the reversal phenomenon of such bands was attributable to the formation of the folded conformer (see Fig. 2c). Indeed, the spectra in acetonitrile at the low pressures are very similar to those observed upon anion recognition in which multiple hydrogen bonds form at the recognition sites (see Fig. 6a). Certainly, at 0.1 MPa, the 0–0 band at 507 nm in acetonitrile seems to be rather hypsochromically shifted compared to that at 516 nm in toluene, 519 nm in chloroform or 519 nm in dichloromethane, which also reinforces the formation of a folded, or conjugation-shortened, conformer. The relatively higher polarity solvent, *i.e*., acetonitrile (*E*_T_ 45.6 kcal mol^−1^), is considered to facilitate an intrachain assembly due to the solvophobic effect.^77^ Hence, the **H** foldamer adopts the extended (**E**) and folded (**F**) conformers in the ground state, as shown in Fig. 4 (*left side*). Most importantly, the relative abundance of the **F** conformer gradually shifted to the **E**-rich state upon the stepwise pressurization on the basis of the 0–0/0–1 band's ratio changes as can be seen in Fig. 3d. This is consistent with the fact that larger *α*_A_ values mean more remarkable conjugation perturbations in π systems by hydrostatic pressure. The pressure-dependent dynamic conformational change is highly likely that solvated molecules around the wide-surface **E** are released easier than the crowded **F**; 
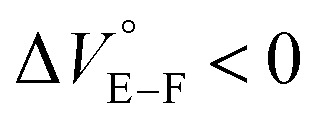
. It is therefore noted that the conformations in the flexible foldamer **H** can dynamically be controlled simply by changing hydrostatic pressure.

**Table 1:** Hydrostatic pressure-induced spectral shifts of the anion receptor (**H**) in each solvent

Solvent	***E***_T_ a/kcal mol^−1^	*α*_A_ b/cm^−1^ MPa^−1^	*α*_F_ c/cm^−1^ MPa^−1^	Δα^d^/cm^−1^ MPa^−1^
Toluene	33.9	−1.05	−1.01	0.04
Chloroform	39.1	−0.97	−0.85	0.12
Dichloromethane	40.7	−0.96	−0.91	0.05
Acetonitrile	45.6	−1.34	−0.91^e^	0.43

a Empirical polarity parameter, determined using Reichardt's dye; see ref. 76.

b Slope of the 0–0 absorption band.

c Slope of the 0–0 fluorescence band.

d Differential slope is *α*_F_ − *α*_A_.

e Until 160 MPa.

On the other hand, all the fluorescence spectra showed gradual bathochromic shifts and quenching in intensity upon the gradual pressurization. The pressurized excitation spectra in Fig. S2† nearly overlapped with the corresponding UV/vis absorption spectrum, indicating the nonexistence of specific fluorescent species. The pressure-induced red shifts in the excited state (*α*_F_) were also calculated by plotting wavenumber changes against pressure (Fig. S4 in the ESI†) as is the case with the UV/vis absorption data (see Table 1). The degree of *α*_F_ (−0.85 to −1.01 cm^−1^ MPa^−1^) is also the same as the data obtained for common fluorescence dyes,^54,57,59^ but the quenching behavior is somewhat puzzling. In general, fluorescence intensities of solutions of emissive dyes upon hydrostatic pressurization follow the Förster–Hoffmann equation [log(*I*_F_) = *A* log(*η*) + *B*],^27^ where fluorescence intensity (*I*_F_) increases with pressure-induced viscosity (*η*) increasing due to the suppression of collisional deactivation by solvent, and thus increase. Nevertheless, the results that the fluorescence quenches were observed in all solvents against the Förster–Hoffmann's behavior can reasonably be accounted as follows. Upon electronic excitation under high pressures, the Franck–Condon state (relatively planar) generated from the ground state (Fig. 4, *center*) may relax to a twisted state (Fig. 4, *right side*). Consequently, solvent reorientation through this planar-to-twisted relaxation process may cause particular quenches by overcoming the pressure-gained viscosity benefits.

**Fig. 5:**
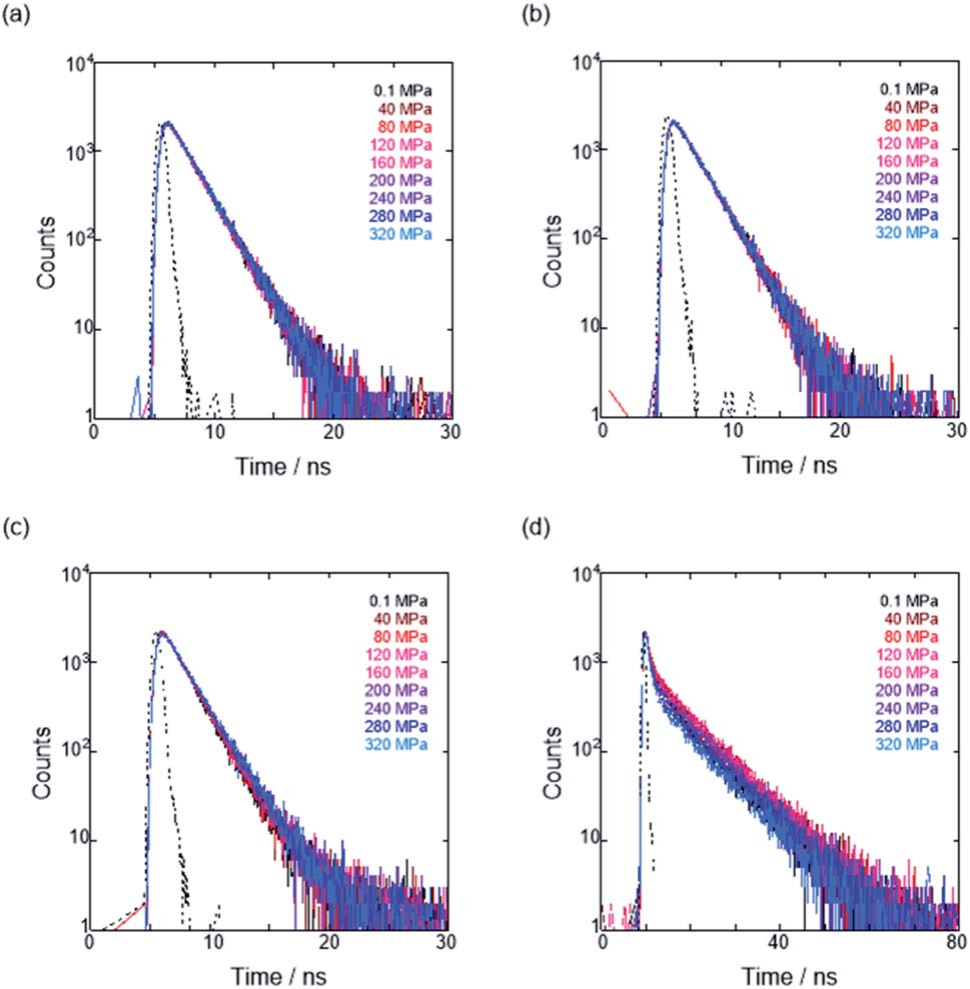
Time-correlated fluorescence decays (*λ*_ex_ 405 nm) of **H** at 0.1, 40, 80, 120, 160, 200, 240, 280 and 320 MPa (from black to sky blue lines) in (a) toluene (44 μM), (b) chloroform (42 μM), (c) dichloromethane (49 μM) and (d) acetonitrile (15 μM) at room temperature, measured in a high-pressure cell. The black dotted line represents the instrument response function.

To further elucidate the origin of fluorescence excited species and the above-mentioned scenario in the excited state, fluorescence lifetimes were measured by the hydrostatic-pressure time-correlated single photon counting method (see Fig. S1 in the ESI† for the set-up). As shown in Fig. 5, the fluorescence decay profiles measured at 550 nm in toluene, chloroform and dichloromethane under high pressures showed appreciable single exponential fitting to give single excited species, indicating that the excited **E** conformer (E*) emits to decay with the lifetime of 1.7–1.9 ns (Table 2; see Table S1 in the ESI† for the data of all lifetimes). Intriguingly, the profiles observed at 546 nm in acetonitrile under high pressures were obviously of multiple components and reasonably fitted to a sum of two exponential functions to afford the lifetimes of 0.6–0.7 ns and 6.5–8.1 ns (Tables 2 and S1†). The lifetime results are consistent with the fact that the dynamic conformational equilibria, determined by the hydrostatic-pressure steady-state UV/vis absorption and fluorescence spectroscopies, exist. Eventually, the shorter-lived species (*τ*_1_) is attributable to E* and the longer-lived one (*τ*_2_) to F* by assuming 
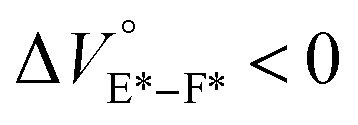
 even in the excited states, since the relative abundance of *τ*_1_ (*τ*_2_) gradually increases (decreases) with increasing pressure. It can be, therefore, emphasized that the fluorescence foldamer's conformational and optical properties are dynamically manipulatable in both ground and excited states by external stimuli such as solvent and particularly hydrostatic pressure.

**Table 2:** Fluorescence lifetimes of the anion receptor (**H**) in each solvent under hydrostatic pressures^a^

Solvent	*λ*_em_ b/nm	*P*/MPa	*τ*_1_	*A*_1_	*τ*_2_	*A*_2_	*χ* ^2^
Toluene	550	0.1	1.8	1.00			1.2
		160	1.9	1.00			1.0
		320	1.9	1.00			1.1
Chloroform	550	0.1	1.9	1.00			1.1
		160	1.8	1.00			1.3
		320	1.9	1.00			1.3
Dichloromethane	550	0.1	1.7	1.00			1.3
		160	1.8	1.00			1.0
		320	1.9	1.00			1.0
Acetonitrile	546	0.1	0.7	0.27	6.5	0.73	1.2
		160	0.6	0.22	7.7	0.78	1.1
		320	0.7	0.38	7.6	0.62	1.2

a Fluorescence lifetime (*τ*_i_) and relative abundance (*A*_i_) of each component, determined by the hydrostatic-pressure single photon counting method in nondegassed solution at room temperature; *λ*_ex_ 405 nm.

b Monitoring wavelength.

### Hydrostatic-pressure effects on anion recognition of the anion receptor (**H**) upon interactions with guests

Next, we investigated the anion-recognition behavior of **H** in solutions upon hydrostatic pressurization by using Cl and Br anions with chiral binaphthylammonium (**SS** or **RR**; see Fig. 2b) or the tetrabutylammonium (TBA) cation as chiral or the achiral ion pair; chloroform as a good solvent was employed in this section, which can dissolve both host–guest combination well even under high pressures.

**Fig. 6:**
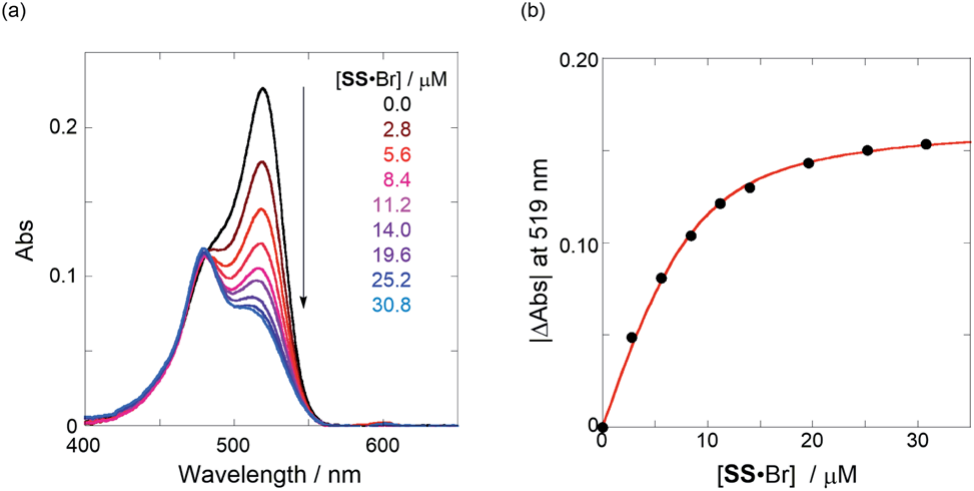
(a) UV/vis absorption spectral changes of a chloroform solution of **H** (8.0 μM, black line) upon gradual addition of **SS**·Br (2.8–30.8 μM, colored line) at room temperature at 40 MPa and (b) nonlinear least-squares fitting, assuming 1 : 1 stoichiometry of **SS**·Br with **H**, to determine the binding constant (*K*_anion_) at room temperature at 40 MPa.

The binding constant (*K*_anion_) of the anions with **H**, defined in Fig. 1a, was determined at room temperature at different pressures by UV/vis absorption spectral titration. As can be seen in Fig. 6a, gradual addition of **SS**·Br (0–30.8 μM) to a chloroform solution of **H** (8.0 μM) led to a steady decrease of the 0–0 band maxima accompanied by the isosbestic point at 481 nm. Nonlinear least-squares fit of the UV/vis absorption spectral changes at 519 nm (Fig. 6b), assuming the 1 : 1 stoichiometry (see the complex structure in Fig. 2c), afforded the *K*_anion_ value of 519 000 M^−1^ at 40 MPa for **SS**·Br. All the titration and fitting data are shown in Fig. S9–S24.† The apparent reaction volume changes 
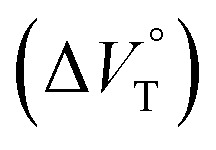
 of the present host–guest complexation were assessed according to eqn (1).1
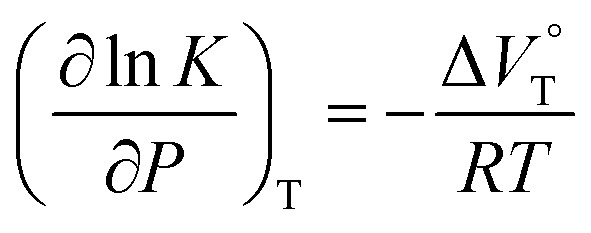


**Fig. 7:**
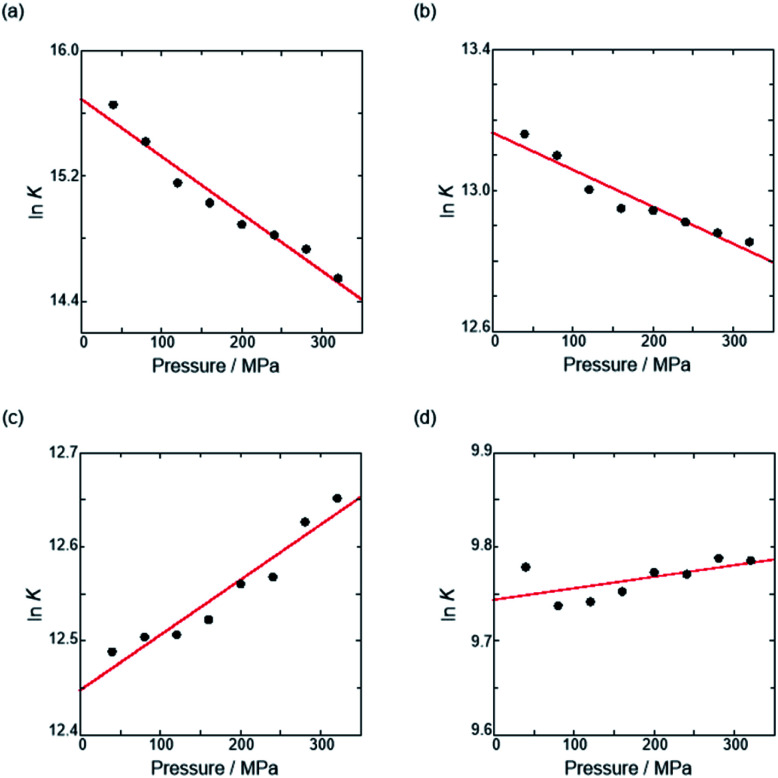
Pressure dependence of the binding constant (*K*_anion_) in anion recognition of **H** with (a) **RR**·Cl, (b) **SS**·Br, (c) TBA·Cl and (d) TBA·Br in chloroform at room temperature.

Thus, the natural logarithm of the *K*_anion_ values obtained at different pressures was plotted as a function of pressure to give good straight lines (Fig. 7 and Table 3; see Tables S2 and S3 in the ESI† for the data of all binding constants), indicating that the 1 : 1 supramolecular complexation mode does not change in the pressure ranges employed (∼320 MPa). The 
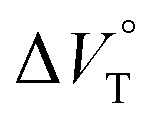
 for the foldamer-anion complexation can be written as eqn (2):2
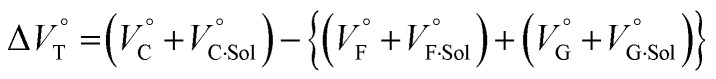
3
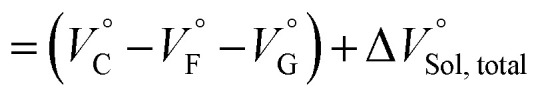
where the subscripts C, F and G represent the van der Waals volume of the complex, foldamer and guest (ion pair), and C·Sol, F·Sol, G·Sol and Sol, total refer to the solvated molar volume of the complex, foldamer, guest (ion pair) and their total at infinite dilution. Eqn (3) was further obtained by rearranging eqn (2). Since the intrinsic volume of the foldamer may not change upon interactions with ion pairs,^31^ we can ignore the first term;
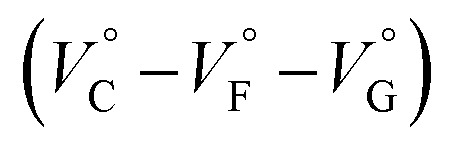
 should be zero. This means that the total volume changes based on solvation 
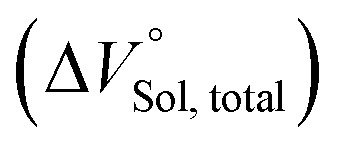
 play critical roles in the evaluation of 
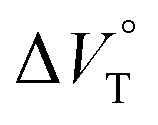
 in the present hydrostatic-pressurized supramolecular system.

**Table 3:** Stoichiometric 1 : 1 binding constant (*K*_anion_) and apparent reaction volume changes 
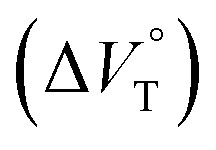
 for the anion receptor (**H**) with some anions in CHCl_3_ under hydrostatic pressures^a^

Guest	*P*/MPa	*K*_anion_/M^−1^	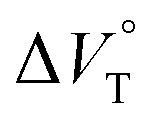 /cm^3^ mol^−1^
**RR**·Cl	40	(6.28 ± 4.17) × 10^6^	+9.1 ± 0.8
	160	(3.36 ± 1.80) × 10^6^	
	320	(2.08 ± 0.91) × 10^6^	
**SS**·Br	40	(5.19 ± 0.56) × 10^5^	+2.6 ± 0.3
	160	(4.20 ± 0.70) × 10^5^	
	320	(3.82 ± 0.67) × 10^5^	
TBA·Cl	40	(2.65 ± 0.28) × 10^5^	−1.5 ± 0.2
	160	(2.74 ± 0.29) × 10^5^	
	320	(3.12 ± 0.39) × 10^5^	
TBA·Br	40	(1.77 ± 0.14) × 10^4^	−0.3 ± 0.2
	160	(1.72 ± 0.18) × 10^4^	
	320	(1.78 ± 0.23) × 10^4^	

a Measured at 298 K.

**Fig. 8:**
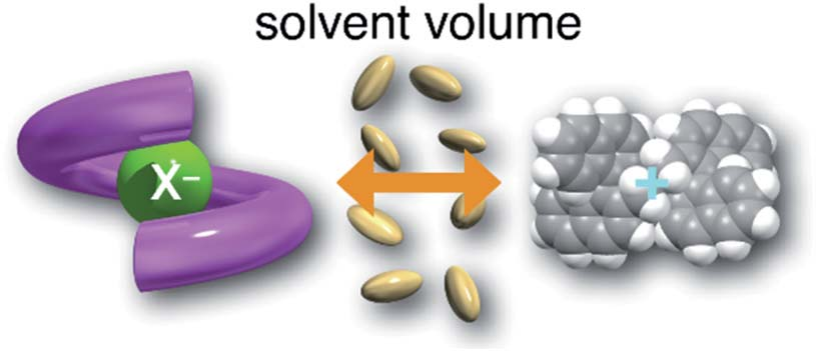
Schematic representation of the ion pairs by solvation.

The order of 
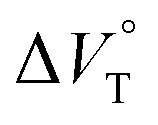
 for the binaphthylammonium salt was Cl^−^ > Br^−^ in positive. Inherently, these values originate from solvated structures that adopt solvent-dependent ion-pairing modes revealed by means of several spectroscopic analyses (Fig. 8).^70^ As noted below, a volumetric-expanded solvation of the solvent-shared ion pair's conformer in the 
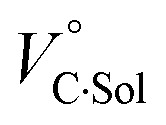
 term turned out to be the main factor controlling 
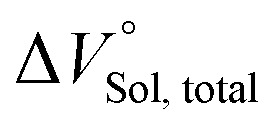
, rather than solvation of free guest ion pairs (
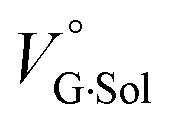
; see the effect on TBA). Certainly, the 
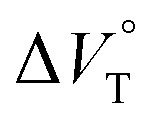
 values for the TBA salts were quite small with the sign inversion in negative. This can reasonably be accounted for in terms of the fact that an applicable volumetric solvation of both the ion-pair complexes may be more difficult than that of free guests due to the smaller van der Waals volume and/or dipole moments of TBA compared to the binaphthylammonium cation.

**Fig. 9:**
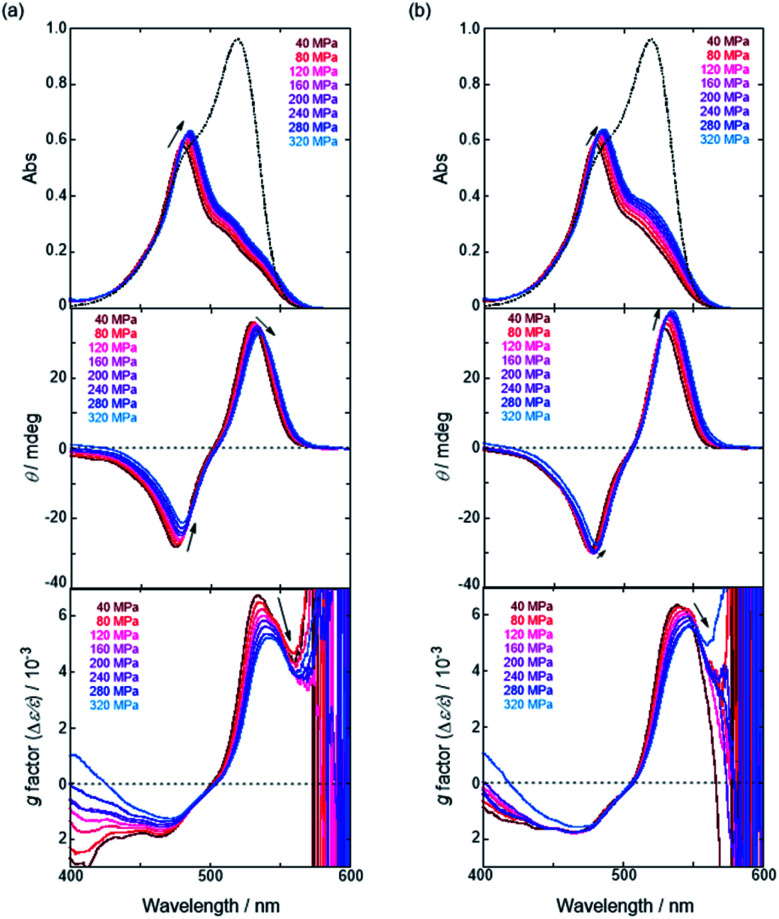
Pressure-dependent UV/vis absorption (top), CD (middle) and anisotropy (*g* factor = Δ*ε*/*ε*) (bottom) spectra of **H** (34 μM) in the presence of (a) **SS**·Cl (120 μM) and (b) **SS**·Br (130 μM) in chloroform at room temperature at 40, 80, 120, 160, 200, 240, 280 and 320 MPa, measured in a high-pressure cell. The dotted lines are UV/vis absorption spectra of **H** (34 μM) in the absence of the corresponding guest at 0.1 MPa.

**Fig. 10:**
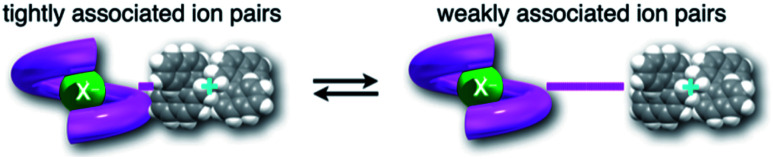
Schematic illustration of the interconversion between tightly and weakly associated ion pairs.

We have already demonstrated that the chiroptical properties of the optically active ion-pair complexes can be traced by means of CD spectroscopy.^70^ Hence, to more clearly elucidate the specific solvation effects on 
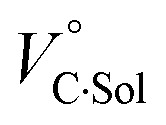
 under high pressures, hydrostatic-pressure CD spectroscopy (see Fig. S1d†) is a rather powerful tool for the use of the unique chiral cation. The examined concentration conditions of host–guest combinations were determined from *K*_anion_ at each pressure (Tables 3 and S3†); [**H**] = 34 μM, [**SS**·Cl] = 120 μM, [**SS**·Br] = 130 μM, and the guest occupancy in **H** was set as >99.9% under pressure conditions performed. As shown in Fig. 9, importantly, anisotropy factor (*g* = Δ*ε*/*ε*) maxima, particularly at the positive band, gradually decreased with pressurization-characteristic bathochromic shifts upon gradual pressurization despite the constant abundance (>99.9%) of each chiral complex. These results strongly indicate that the pressure-induced equilibrium in the complex conformers shifts to a chirally weakened complex as a weakly associated ion pair (Fig. 10). As seen in Fig. S25 in the ESI,† on using Cl^−^, a larger decrease of *g* values at the positive maxima (slope of −7.66 × 10^−6^ MPa^−1^) was observed compared to the use of Br^−^ (slope of −4.77 × 10^−6^ MPa^−1^), facilitating further formation of the weakly associated ion pair upon pressurization probably due to the stronger interaction of Cl^−^ with **H**. The pressure-induced formation of this volumetric-expanded conformer again reinforces the origin of the considerable differences in 
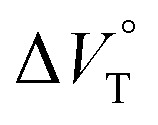
.

**Fig. 11:**
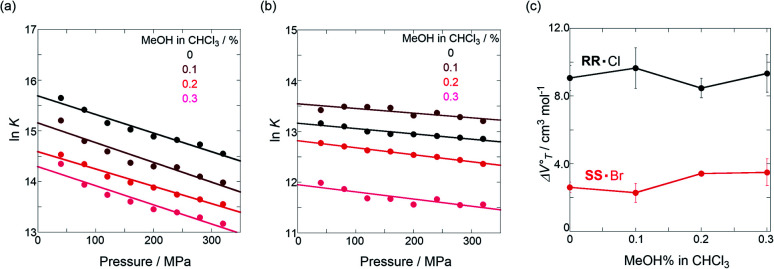
Pressure dependence of the binding constant (*K*_anion_) obtained in the supramolecular complexation of (a) **RR**·Cl and (b) **SS**·Br with **H** under hydrostatic pressures (40–320 MPa) at room temperature in chloroform containing methanol (0–0.3%). (c) Plots of 
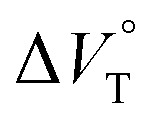
 as a function of methanol content in chloroform.

To finally examine pressure effects on the solvated complexes, we mixed small amounts of methanol as a solvated solvent with chloroform solutions, and then subjected to the same hydrostatic-pressure titration experiments (Fig. S13–S24†) to afford a series of *K*_anion_ and 
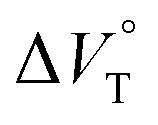
 values (Fig. 11 and Table 4). Unexpectedly, both 
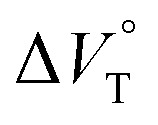
 values for Cl^−^ and Br^−^ are constant, irrespective of the methanol content of ∼0.3%, while *K*_anion_ decreased with increasing methanol concentration. This unprecedented 
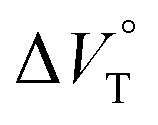
 behavior is highly likely to be mutual specific solvation of methanol to the complex 
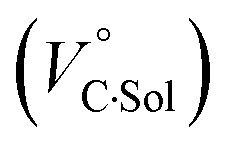
 and free guest 
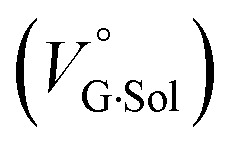
.

**Table 4:** Apparent reaction volume changes 
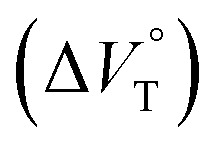
 in CHCl_3_ containing MeOH (0–0.3%)^a^

Guest	MeOH/%	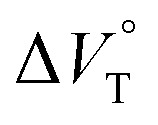 /cm^3^ mol^−1^
**RR**·Cl	0	+9.1 ± 0.8
	0.1	+9.7 ± 1.2
	0.2	+8.5 ± 0.6
	0.3	+9.3 ± 1.1
**SS**·Br	0	+2.6 ± 0.3
	0.1	+2.3 ± 0.6
	0.2	+3.4 ± 0.1
	0.3	+3.5 ± 0.8

a Measured at 298 K; [**H**] = 6.0–9.4 μM.

## Conclusions

We have demonstrated the ground- and excited-state dynamic control of the anion receptor **H** induced by hydrostatic pressure. The extended (**E**) and folded (**F**) conformers of **H** are mutually regulatable in response to solvation as well as hydrostatic pressure. Intriguingly, the optical properties based on these two conformers were very different from each other, thus leading to the creation of a hydrostatic-pressure-responsive optical molecular spring. The solvations of the supramolecular complexes play pivotal roles in the anion recognition behavior of **H** with achiral and chiral ion pairs, quantitatively revealed by the apparent reaction volume changes 
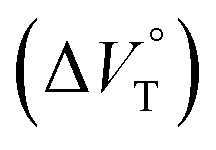
. Eventually, it is noteworthy that the strategy proposed herein, *i.e*., the hydrostatic-pressure control, may be extended to other foldamer and guest combinations that are difficult to sense each other or to non-lock-and-key systems.

## Author contributions

G. F. initiated the project. G. F. and H. M. designed the experiments. T. K. carried out spectroscopic experiments, analyzed the data, and prepared the manuscript. Y. H. prepared the materials. All authors revised the manuscript.

## Conflicts of interest

There are no conflicts to declare.

## Supplementary Material

SC-012-D1SC00664A-s001
