# Supplementary material for: Ground- and excited-state dynamic control of an anion receptor by hydrostatic pressure
Source: Chem Sci. 2021 Apr 15;12(19):6691–8. doi: 10.1039/d1sc00664a (PMC8132960; doi:10.1039/d1sc00664a)
Supplement: SC-012-D1SC00664A-s001 [file SC-012-D1SC00664A-s001.pdf]

Electronic Supplementary Material (ESI) for Chemical Science.
This journal is © The Royal Society of Chemistry 2021

*Electronic Supplementary Information*

# Ground- and excited-state dynamic control of an anion receptor by hydrostatic pressure

*Tomokazu Kinoshita,*[a] *Yohei Haketa,*[a] *Hiromitsu Maeda*[*a] *and Gaku Fukuhara*[*ac]

[a]Department of Chemistry, Tokyo Institute of Technology, 2-12-1 Ookayama, Meguro-ku, Tokyo 152-8551, Japan

[b]Department of Applied Chemistry, College of Life Sciences, Ritsumeikan University, 1-1-1 Nojihigashi, Kusatsu 525-8577, Japan

[c]JST, PRESTO, 4-1-8 Honcho, Kawaguchi, Saitama 332-0012, Japan

*E-mail: maedahir@ph.ritsumei.ac.jp (H.M.); gaku@chem.titech.ac.jp (G.F.)

## EXPERIMENTAL SCTION

**General.** UV/vis absorption, CD, and fluorescence spectra were recorded by using a JASCO V-560, JASCO J-720WI, and JASCO FP-8500 spectrometers, respectively. Fluorescence lifetimes were measured by a Hamamatsu Quantaurus-Tau single photon counting apparatus fitted with an LED light ($\lambda_{ex}$ 405 nm).

**Materials.** Spectrophotometric-grade solvents (toluene, dichloromethane, chloroform, acetonitrile, and methanol) were used without further purification. The anion receptor (**H**) and chiral guests were prepared, and the data thus obtained showed satisfactory agreement with the literatures.[69,70] Binaphthylammonium **RR** and **SS** cations as chiral sources are mutually enantiomers. Therefore, these chiral cations afford same results (*i.e.* anion-binding behaviors and pressure-responsive properties), except for the signs of CD spectra derived from the helical conformations of **H**.

**Hydrostatic-pressure spectroscopy.** All spectroscopic experiments were performed by a custom-built high-pressure apparatus.[53] Briefly, sapphire windows were used for UV/vis absorption and fluorescence spectroscopy and lifetime measurements, while birefringence-free diamond windows were employed for CD spectroscopy. A quartz cell for high pressure (3 mm width, 2 mm depth, and 7 mm height) was filled with a sample solution. The solution was pressurized by water from 0.1 to 320 MPa (see Figure S1).

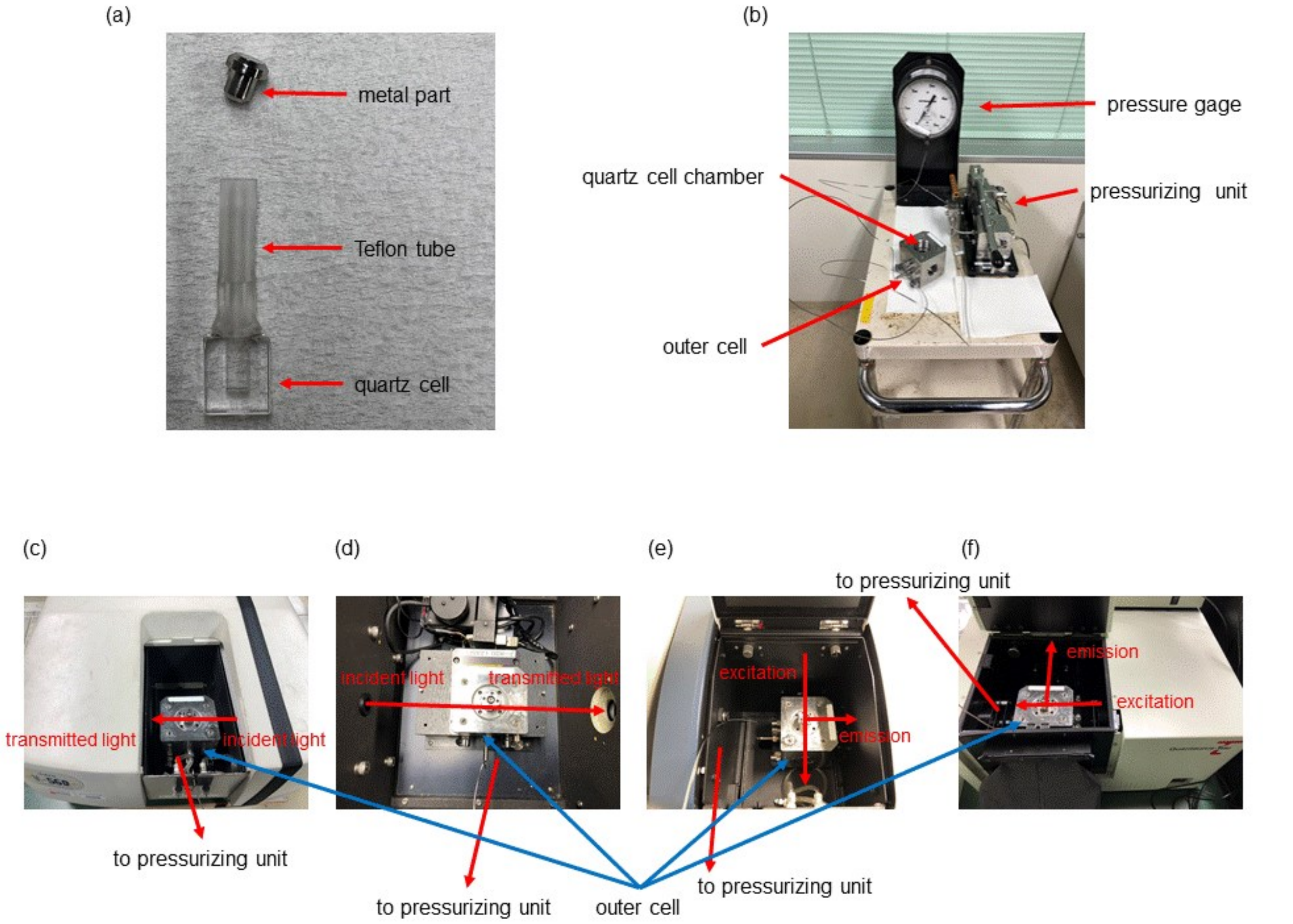


**Figure S1.** Photographs of (a) quartz cell, (b) the pressurizing apparatus, and the set up for (c) UV/vis absorption, (d) circular dichroism, (e) fluorescence, and (d) lifetime measurements. Reproduced from ref. 59 with permission. Copyright 2020 Wiley-VCH.

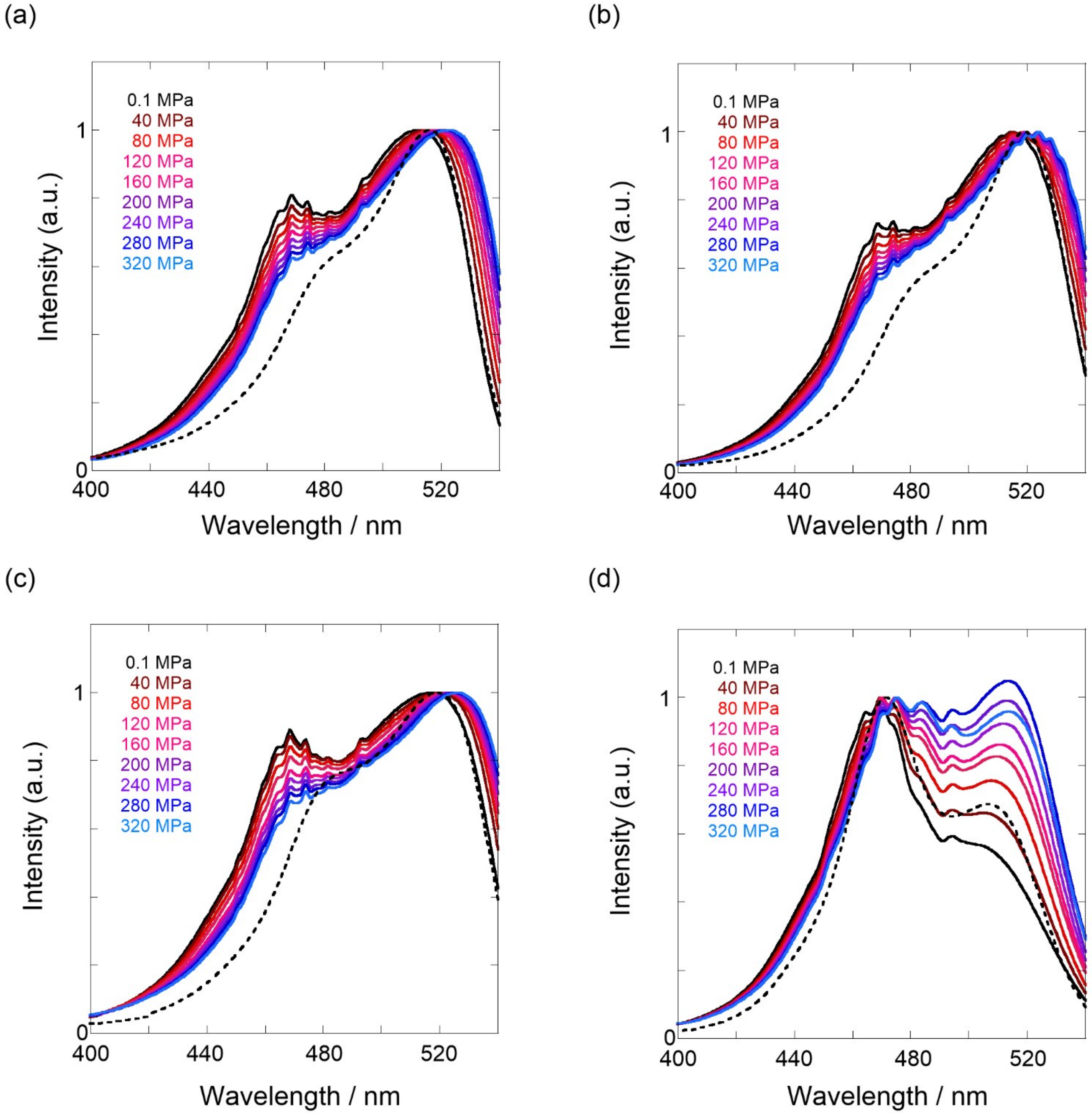


**Figure S2.** Excitation spectra ($\lambda_{em}$ 550 nm) of **H** at 0.1, 40, 80, 120, 160, 200, 240, 280, and 320 MPa (from black to sky blue lines) in (a) toluene (44 μM), (b) chloroform (42 μM), (c) dichloromethane (49 μM), and (d) acetonitrile (15 μM) at room temperature, measured in a high-pressure cell. Black dotted line represents the normalized UV/vis absorption spectrum at 0.1 MPa.

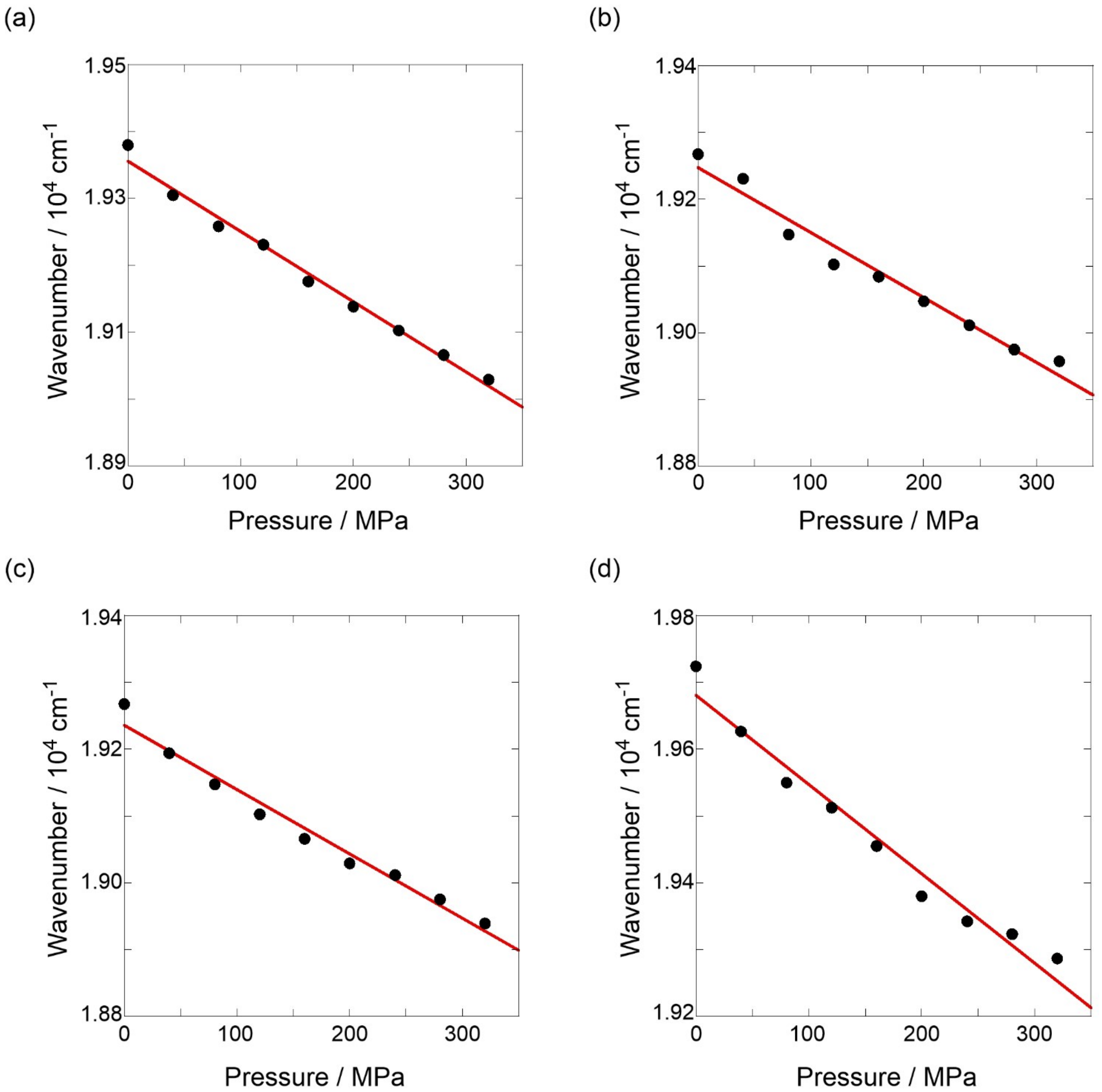


**Figure S3.** Plots of wavenumber changes for the pressure-induced 0-0 absorption maxima of **H**; (a) in toluene, correlation coefficient $r$ = 0.995, slope; –1.05 cm$^{-1}$ MPa$^{-1}$, (b) in chloroform, $r$ = 0.985, slope; –0.97 cm$^{-1}$ MPa$^{-1}$, (c) in dichloromethane, $r$ = 0.988, slope; –0.96 cm$^{-1}$ MPa$^{-1}$, and (d) in acetonitrile, $r$ = 0.984, slope; –1.34 cm$^{-1}$ MPa$^{-1}$.

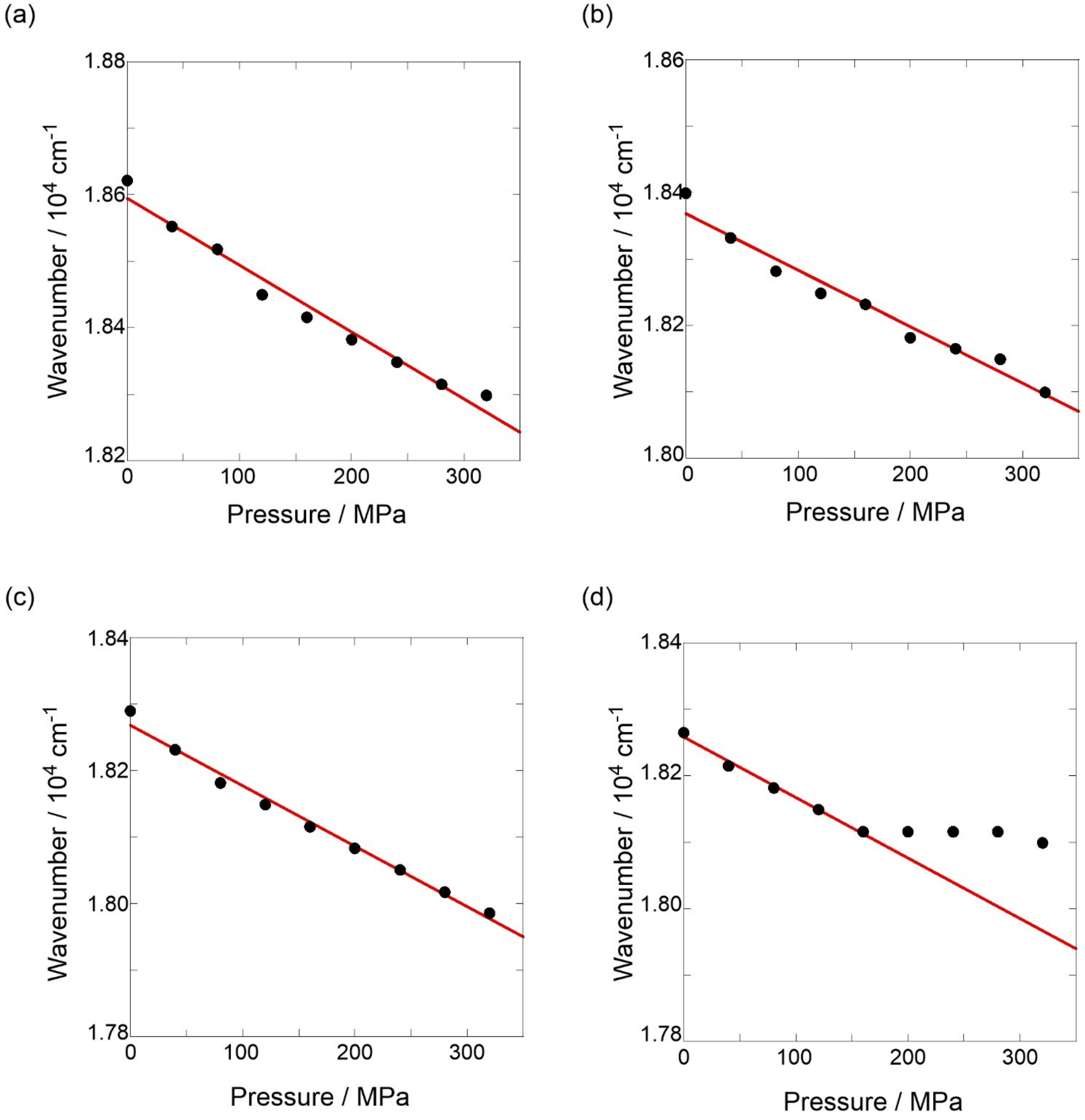


**Figure S4.** Plots of wavenumber changes for the pressure-induced fluorescence maxima of **H**; (a) in toluene, $r$ = 0.988, slope; –1.01 cm$^{-1}$ MPa$^{-1}$, (b) in chloroform, $r$ = 0.984, slope; –0.85 cm$^{-1}$ MPa$^{-1}$, (c) in dichloromethane, $r$ = 0.994, slope; –0.91 cm$^{-1}$ MPa$^{-1}$, and (d) in acetonitrile, $r$ = 0.996, slope; –0.91 cm$^{-1}$ MPa$^{-1}$.

**Table S1. Fluorescence Lifetimes of the Anion Receptor (H) in Each Solvent under Hydrostatic Pressures[a]**

| Solvent | $\lambda_{em}{}^{b}$/nm | $P$/MPa | $\tau_1$/ns | $A_1$ | $\tau_2$/ns | $A_2$ | $\chi^2$ |
|---|---|---|---|---|---|---|---|
| toluene | 550 | 0.1 | 1.8 | | | | 1.2 |
| | | 40 | 1.8 | | | | 1.0 |
| | | 80 | 1.9 | | | | 0.7 |
| | | 120 | 1.9 | | | | 0.9 |
| | | 160 | 1.9 | | | | 1.0 |
| | | 200 | 1.9 | | | | 1.0 |
| | | 240 | 1.9 | | | | 1.0 |
| | | 280 | 1.9 | | | | 1.1 |
| | | 320 | 1.9 | | | | 1.1 |
| chloroform | 550 | 0.1 | 1.9 | | | | 1.1 |
| | | 40 | 1.9 | | | | 1.3 |
| | | 80 | 1.9 | | | | 1.1 |
| | | 120 | 1.9 | | | | 1.2 |
| | | 160 | 1.8 | | | | 1.3 |
| | | 200 | 1.9 | | | | 1.1 |
| | | 240 | 1.9 | | | | 1.1 |
| | | 280 | 1.9 | | | | 1.3 |
| | | 320 | 1.9 | | | | 1.3 |
| dichloromethane | 550 | 0.1 | 1.7 | | | | 1.3 |
| | | 40 | 1.7 | | | | 1.3 |
| | | 80 | 1.7 | | | | 1.3 |
| | | 120 | 1.8 | | | | 1.3 |
| | | 160 | 1.8 | | | | 1.0 |
| | | 200 | 1.8 | | | | 1.3 |
| | | 240 | 1.8 | | | | 1.3 |
| | | 280 | 1.9 | | | | 1.3 |
| | | 320 | 1.9 | | | | 1.0 |
| acetonitrile | 550 | 0.1 | 0.7 | 0.27 | 6.5 | 0.73 | 1.2 |
| | | 40 | 0.7 | 0.21 | 7.1 | 0.79 | 1.3 |
| | | 80 | 0.7 | 0.23 | 7.6 | 0.77 | 1.3 |
| | | 120 | 0.6 | 0.19 | 7.8 | 0.81 | 1.2 |
| | | 160 | 0.6 | 0.22 | 7.7 | 0.78 | 1.1 |
| | | 200 | 0.7 | 0.27 | 8.1 | 0.73 | 1.2 |
| | | 240 | 0.6 | 0.30 | 7.8 | 0.70 | 1.3 |
| | | 280 | 0.6 | 0.34 | 7.6 | 0.66 | 1.3 |
| | | 320 | 0.7 | 0.38 | 7.6 | 0.62 | 1.2 |

[a] Fluorescence lifetime ($\tau_i$) and relative abundance ($A_i$) of each component, determined by the hydrostatic-pressure single photon counting method in nondegassed solution at room temperature; $\lambda_{ex}$ 405 nm. [b] Monitoring wavelength.

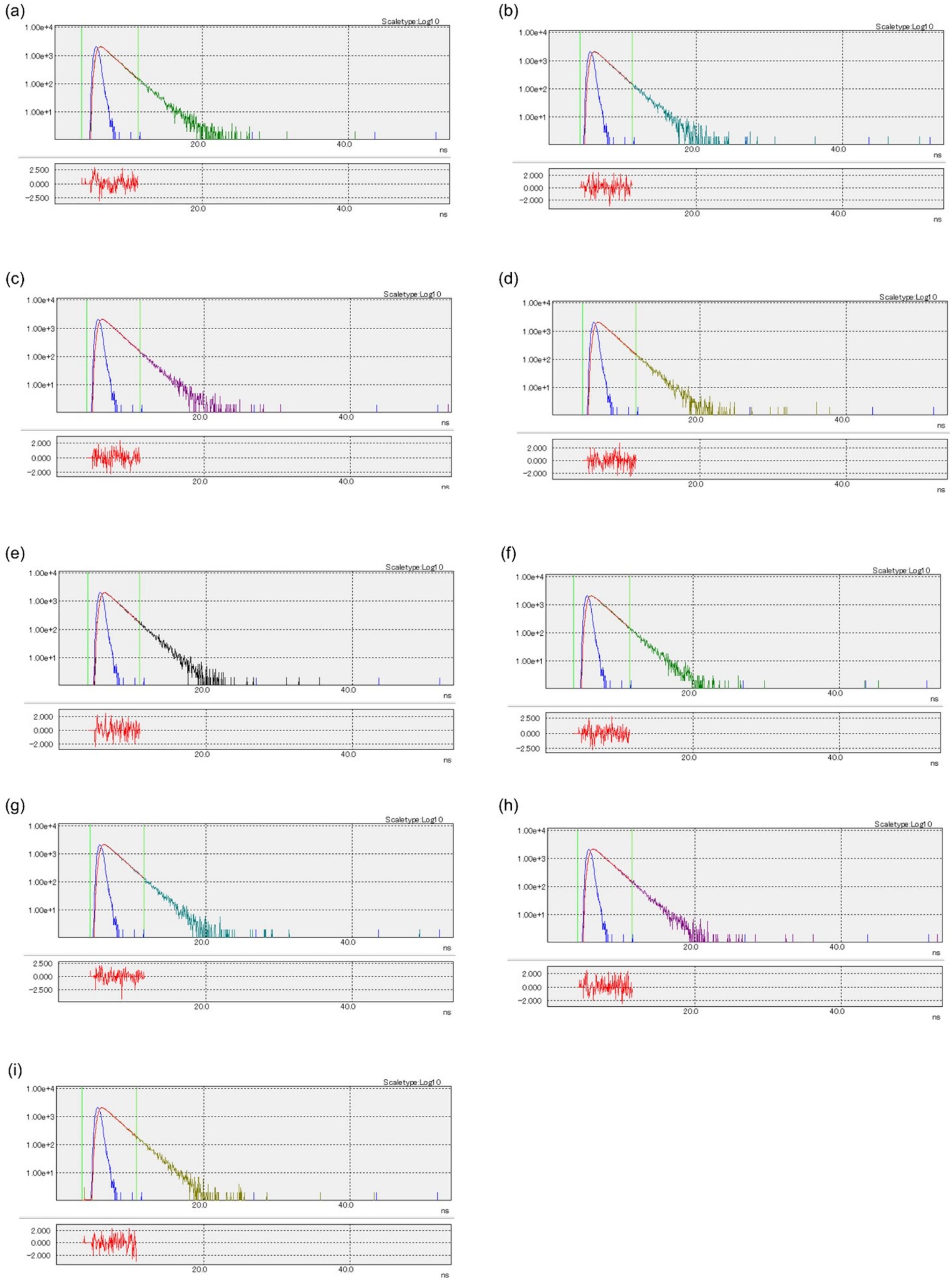


**Figure S5.** Time-correlated fluorescence decays of **H** in toluene (44 μM) monitored at 550 nm at (a) 0.1, (b) 40, (c) 80, (d) 120, (e) 160, (f) 200, (g) 240, (h) 280, and (i) 320 MPa at room temperature, measured in a high-pressure cell, where the colored, red, and blue lines represent the fluorescence decay, fitting result, and the instrument response function, respectively.

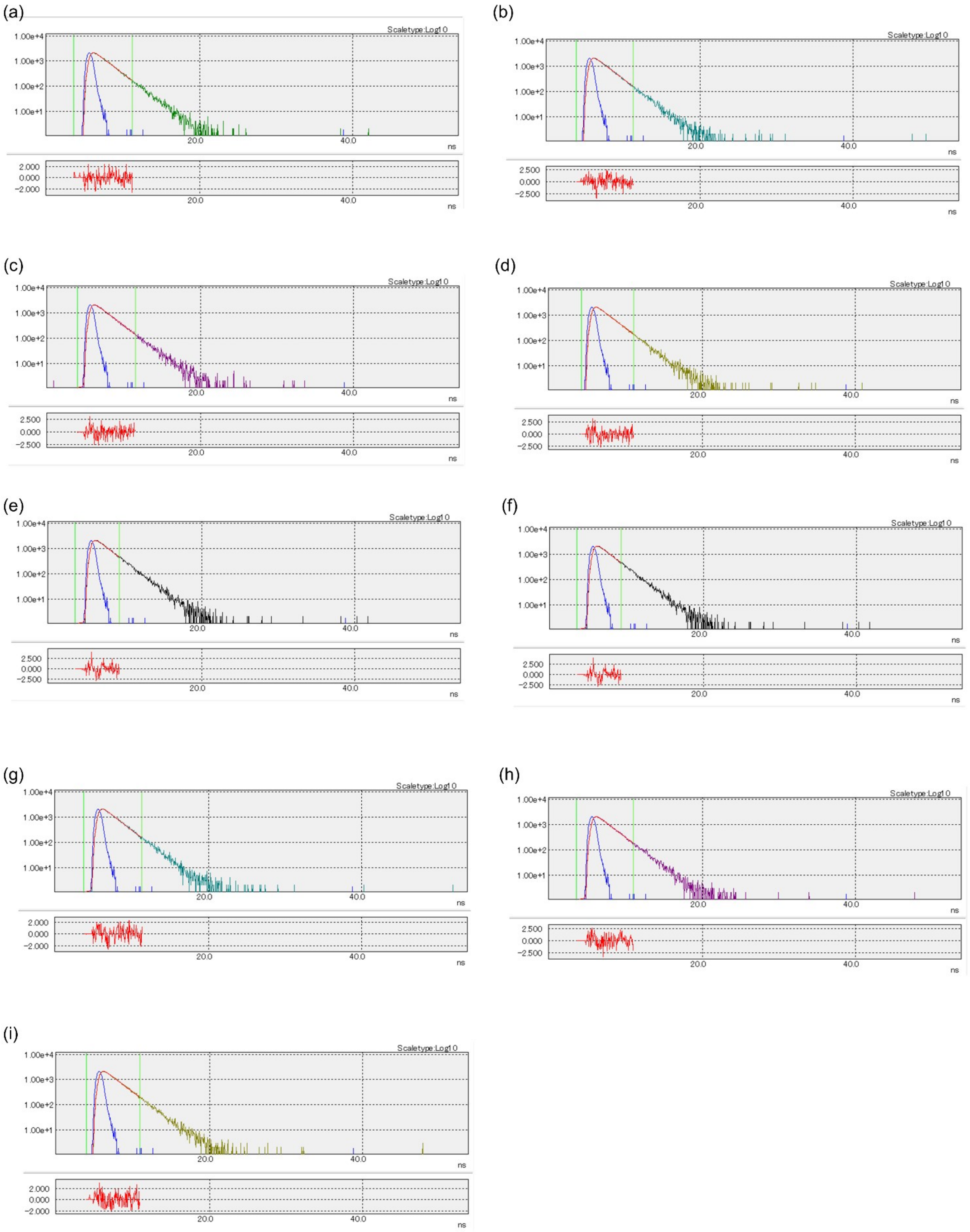


**Figure S6.** Time-correlated fluorescence decays of **H** in chloroform (42 μM) monitored at 550 nm at (a) 0.1, (b) 40, (c) 80, (d) 120, (e) 160, (f) 200, (g) 240, (h) 280, and (i) 320 MPa at room temperature, measured in a high-pressure cell, where the colored, red, and blue lines represent the fluorescence decay, fitting result, and the instrument response function, respectively.

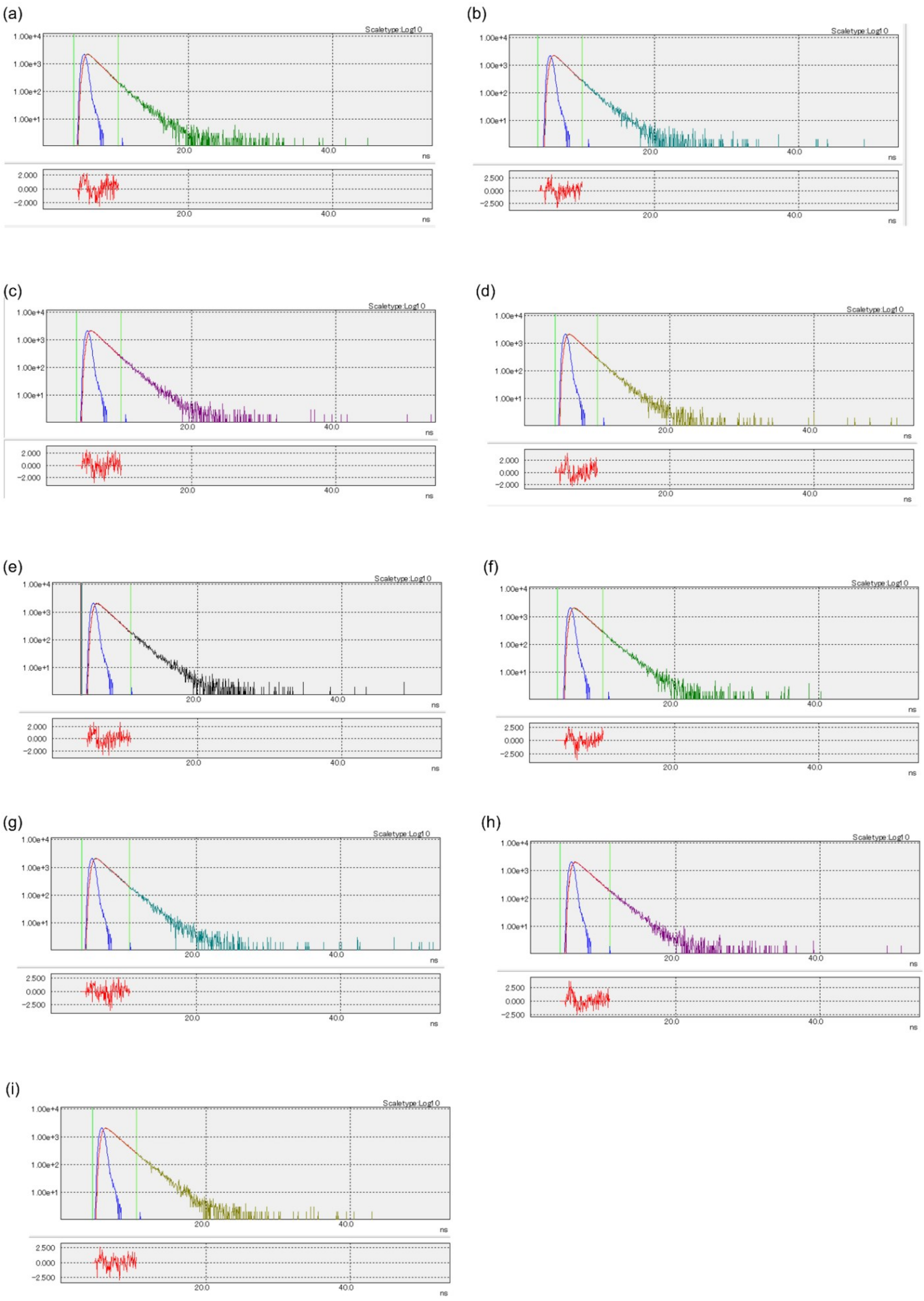

**Figure S7.** Time-correlated fluorescence decays of **H** in dichloromethane (49 μM) monitored at 550 nm at (a) 0.1, (b) 40, (c) 80, (d) 120, (e) 160, (f) 200, (g) 240, (h) 280, and (i) 320 MPa at room temperature, measured in a high-pressure cell, where the colored, red, and blue lines represent the fluorescence decay, fitting result, and the instrument response function, respectively.

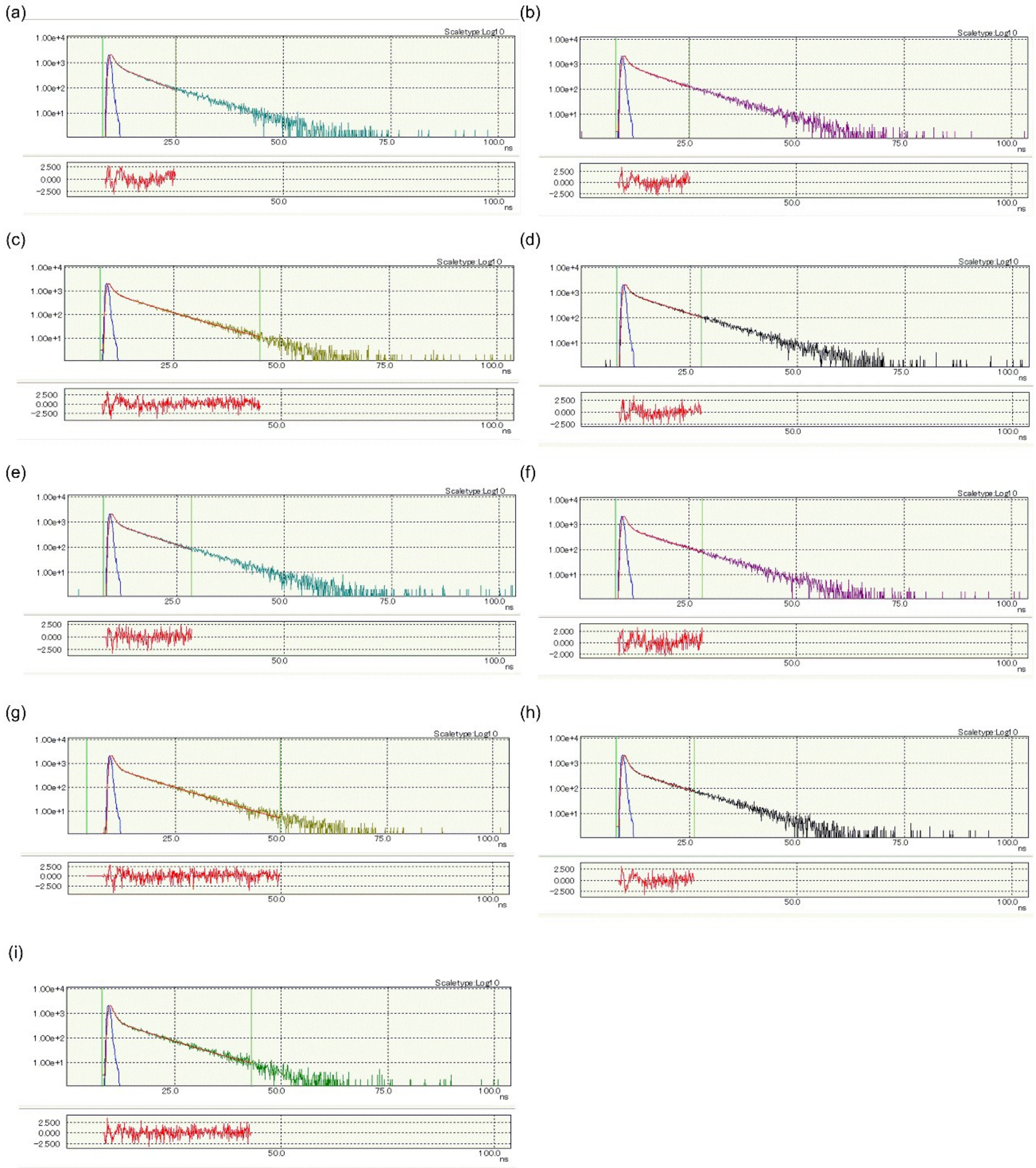


**Figure S8.** Time-correlated fluorescence decays of **H** in acetonitrile (15 μM) monitored at 546 nm at (a) 0.1, (b) 40, (c) 80, (d) 120, (e) 160, (f) 200, (g) 240, (h) 280, and (i) 320 MPa at room temperature, measured in a high-pressure cell, where the colored, red, and blue lines represent the fluorescence decay, fitting result, and the instrument response function, respectively.

(a)

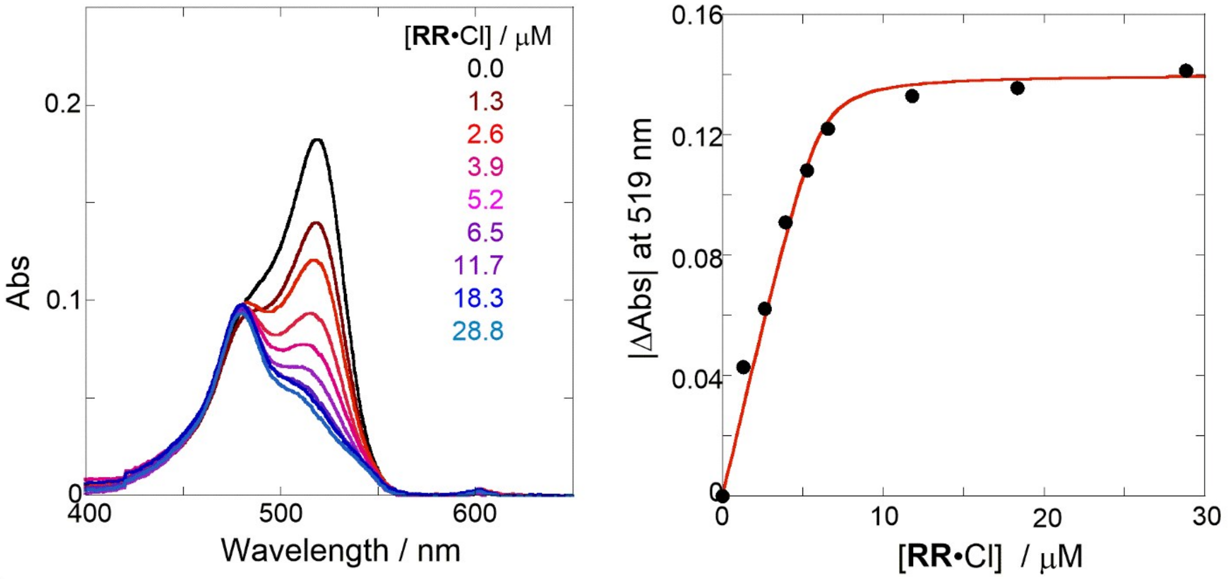
[RR•Cl] / μM
0.0
1.3
2.6
3.9
5.2
6.5
11.7
18.3
28.8
Abs
Wavelength / nm
|ΔAbs| at 519 nm
[RR•Cl] / μM

(b)

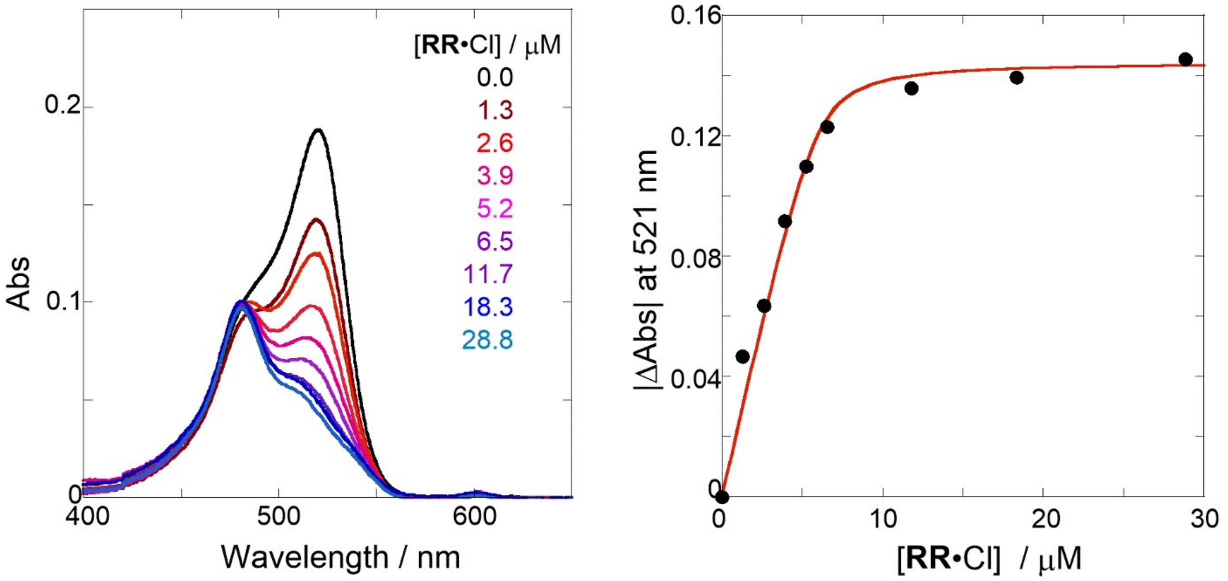
[RR•Cl] / μM
0.0
1.3
2.6
3.9
5.2
6.5
11.7
18.3
28.8
Abs
Wavelength / nm
|ΔAbs| at 521 nm
[RR•Cl] / μM

(c)

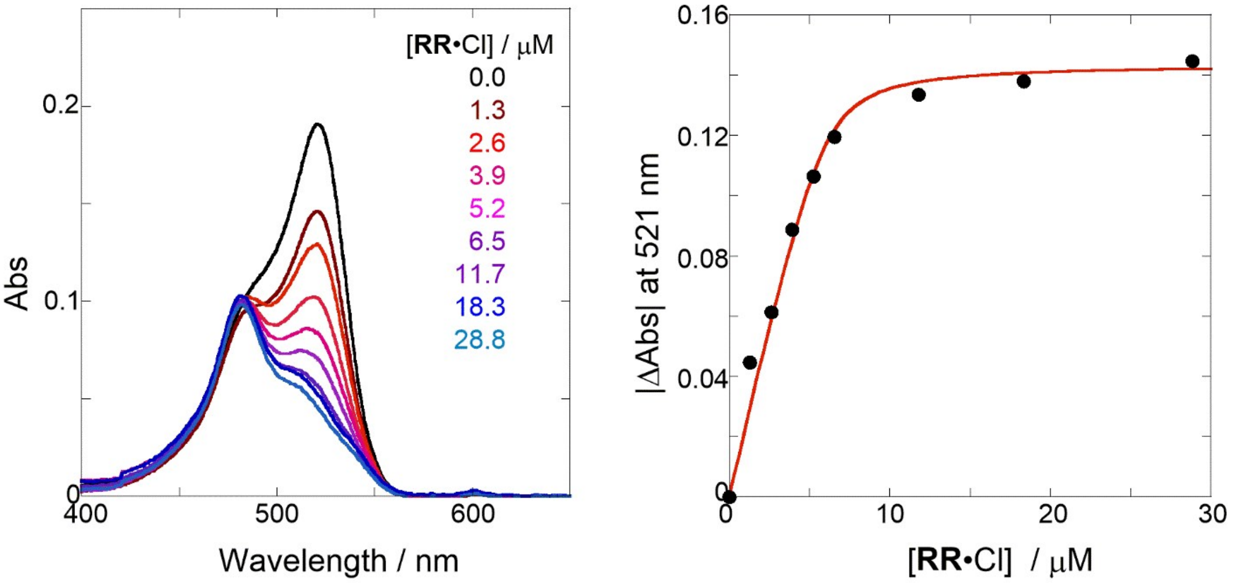
[RR•Cl] / μM
0.0
1.3
2.6
3.9
5.2
6.5
11.7
18.3
28.8
Abs
Wavelength / nm
|ΔAbs| at 521 nm
[RR•Cl] / μM

(d)

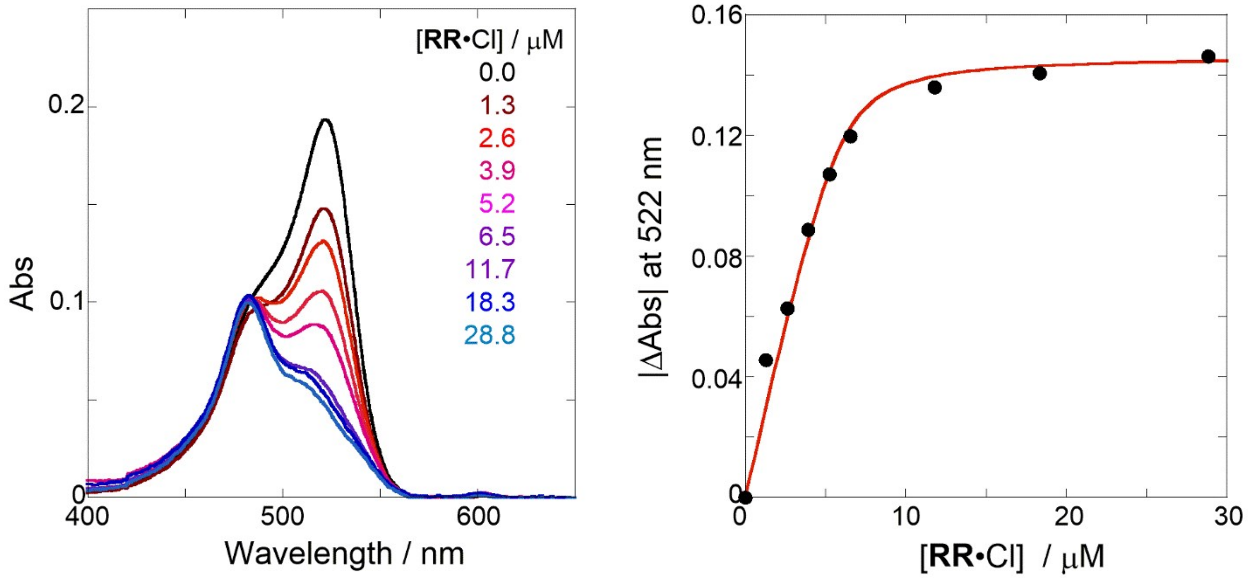
[RR•Cl] / μM
0.0
1.3
2.6
3.9
5.2
6.5
11.7
18.3
28.8
Abs
Wavelength / nm
|ΔAbs| at 522 nm
[RR•Cl] / μM

(e)

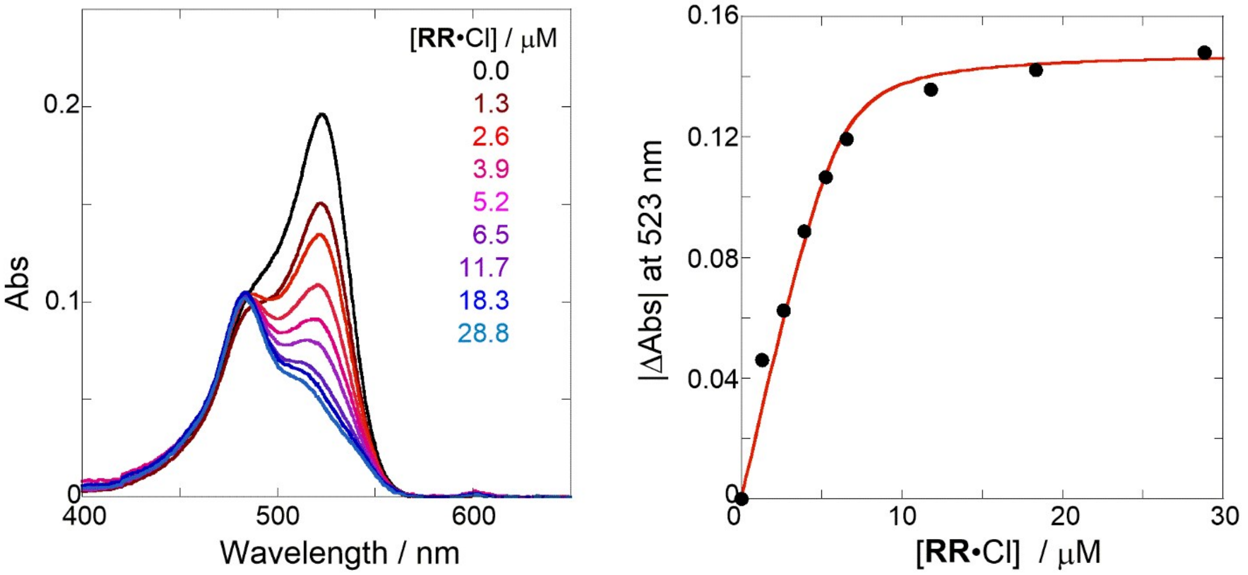
[RR•Cl] / μM
0.0
1.3
2.6
3.9
5.2
6.5
11.7
18.3
28.8
Abs
Wavelength / nm
|ΔAbs| at 523 nm
[RR•Cl] / μM

(f)

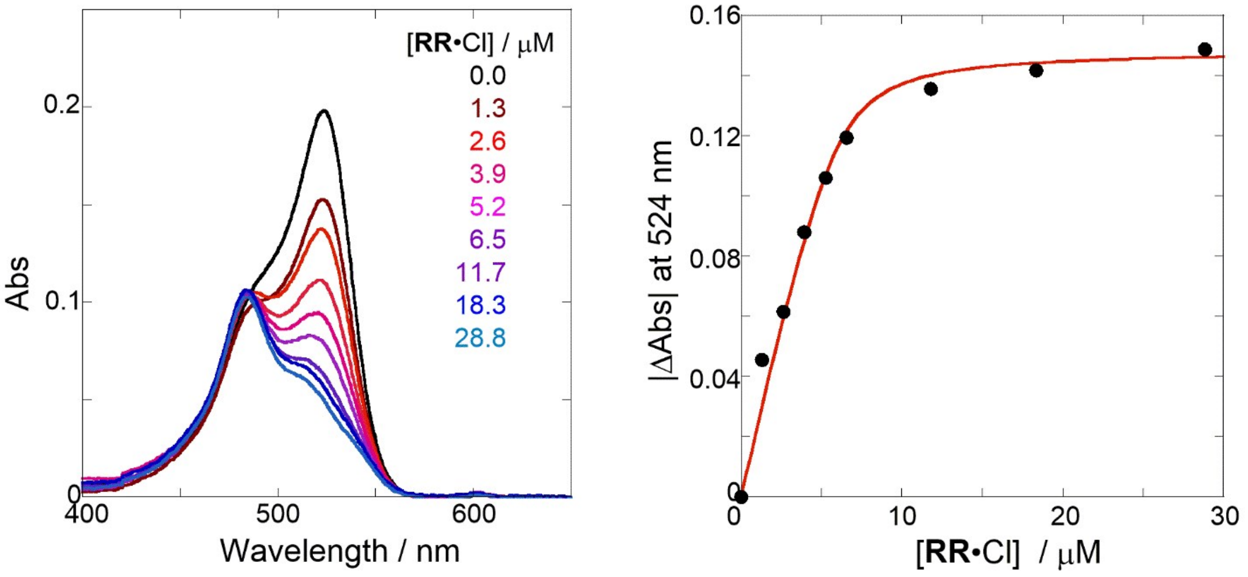
[RR•Cl] / μM
0.0
1.3
2.6
3.9
5.2
6.5
11.7
18.3
28.8
Abs
Wavelength / nm
|ΔAbs| at 524 nm
[RR•Cl] / μM

(g)

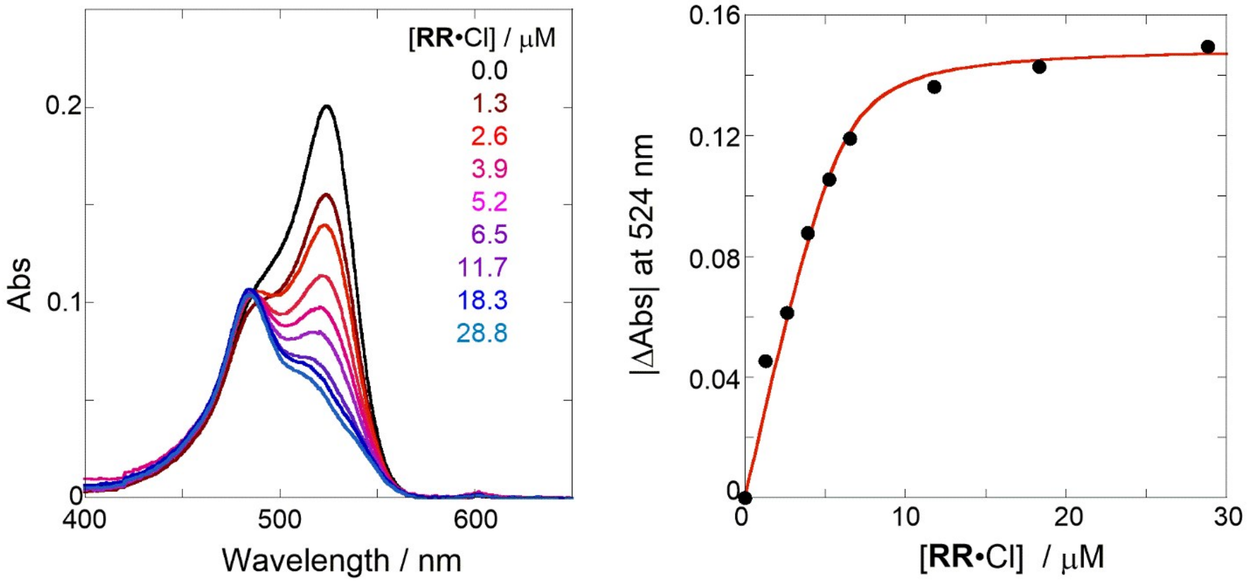


(h)

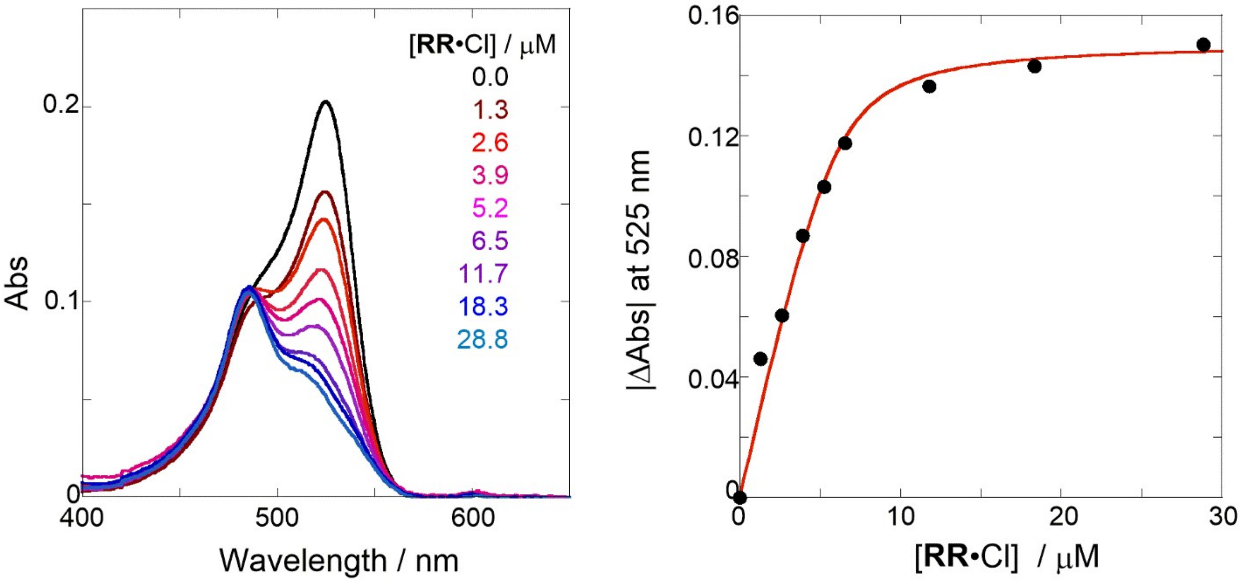


**Figure S9.** (*Left panels*) UV/vis absorption spectral changes of a chloroform solution of **H** (6.0 μM, black line) upon gradual addition of **RR**•Cl (1.3–28.8 μM, colored line) and (*Right panels*) nonlinear least-squares fitting, assuming 1:1 stoichiometry of **RR**•Cl with **H**, to determine the binding constant ($K_{anion}$) at room temperature at (a) 40, (b) 80, (c) 120, (d) 160, (e) 200, (f) 240, (g) 280, and (h) 320 MPa, measured in a high-pressure cell.

(a)

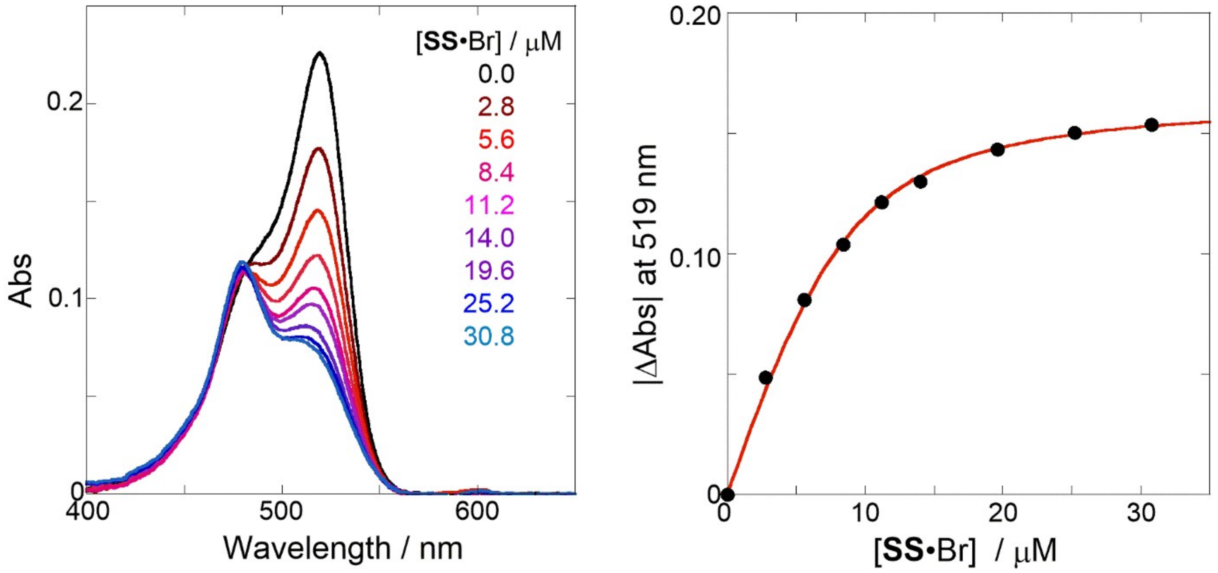

[SS•Br] / μM
0.0
2.8
5.6
8.4
11.2
14.0
19.6
25.2
30.8
Abs
0.2
0.1
0
400
500
600
Wavelength / nm
0.20
0.10
0
10
20
30
|ΔAbs| at 519 nm
[SS•Br] / μM


(b)

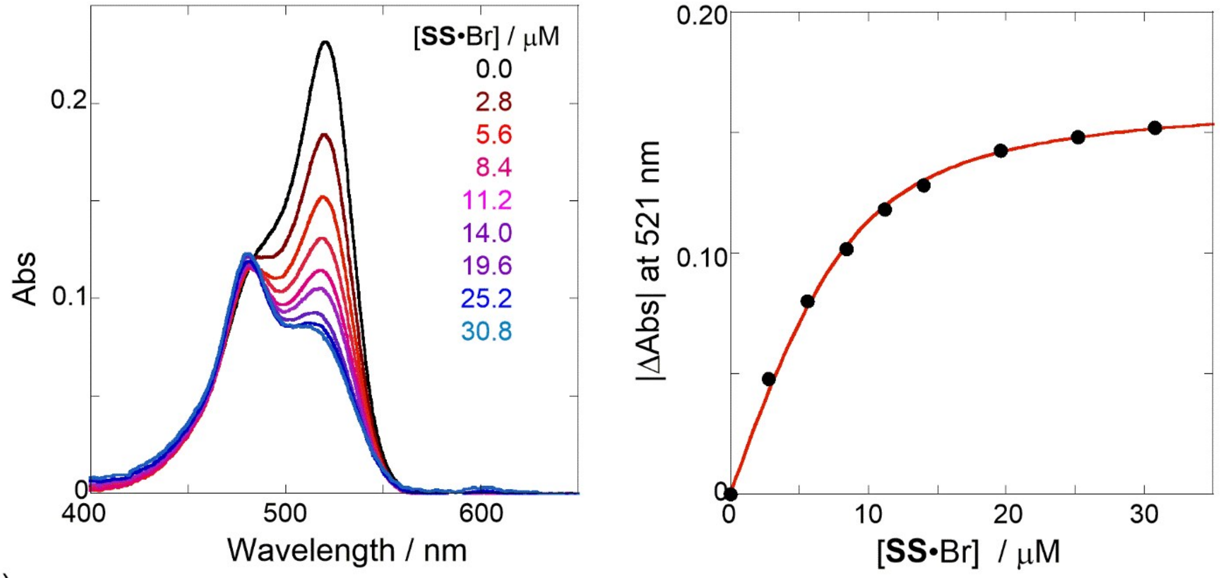

[SS•Br] / μM
0.0
2.8
5.6
8.4
11.2
14.0
19.6
25.2
30.8
Abs
0.2
0.1
0
400
500
600
Wavelength / nm
0.20
0.10
0
10
20
30
|ΔAbs| at 521 nm
[SS•Br] / μM


(c)

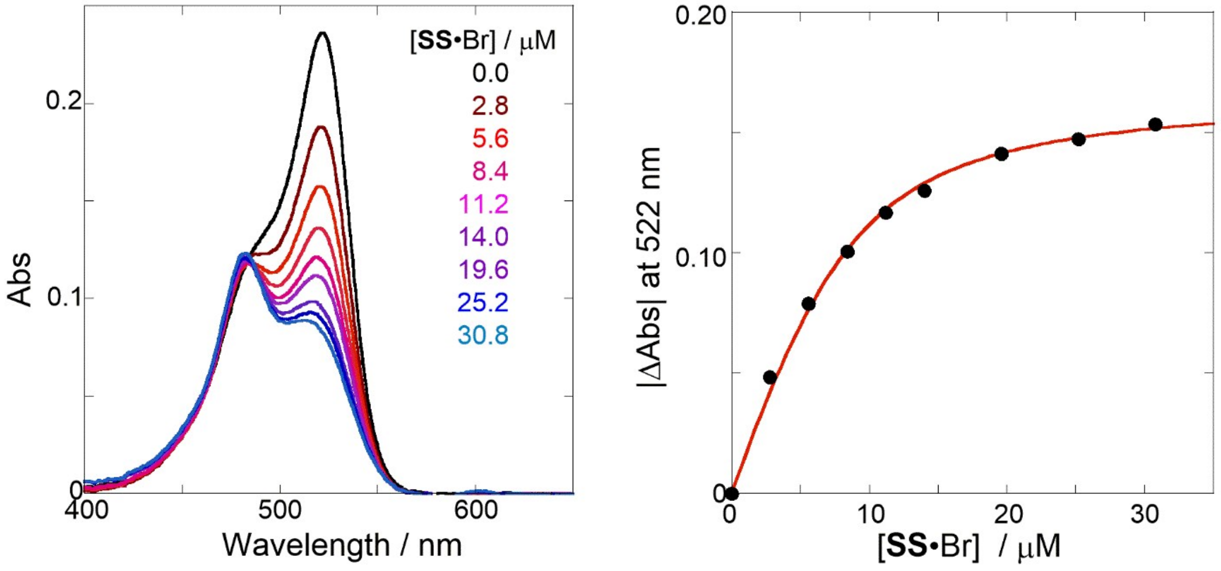

[SS•Br] / μM
0.0
2.8
5.6
8.4
11.2
14.0
19.6
25.2
30.8
Abs
0.2
0.1
0
400
500
600
Wavelength / nm
0.20
0.10
0
10
20
30
|ΔAbs| at 522 nm
[SS•Br] / μM

(d)

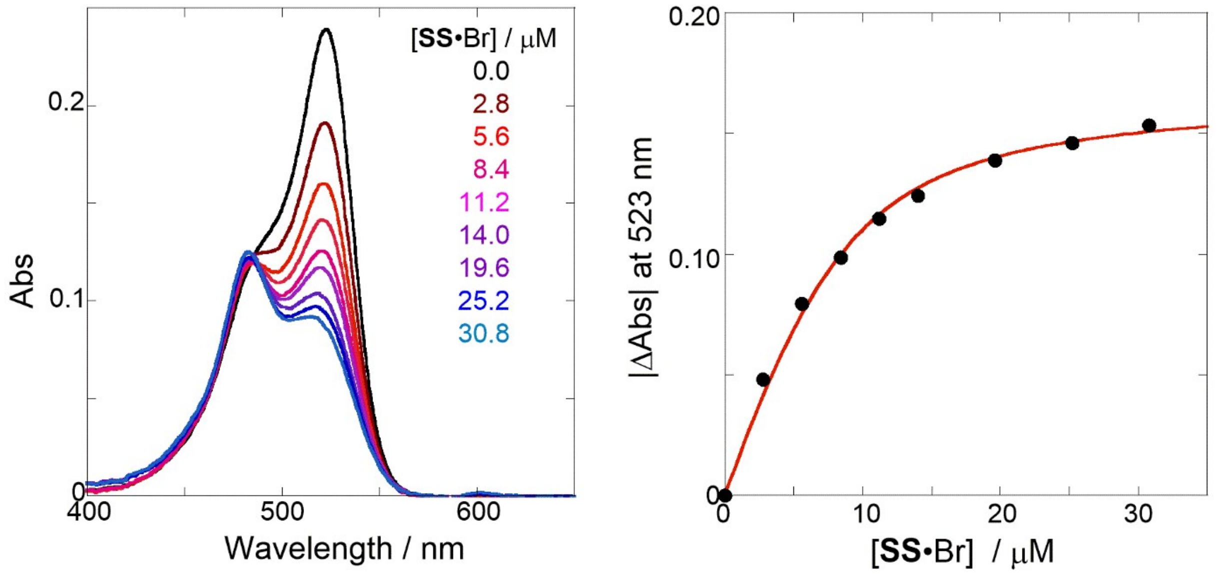
[SS•Br] / μM
0.0
2.8
5.6
8.4
11.2
14.0
19.6
25.2
30.8
Abs
0
0.1
0.2
400
500
600
Wavelength / nm
|ΔAbs| at 523 nm
0
0.10
0.20
0
10
20
30
[SS•Br] / μM

(e)

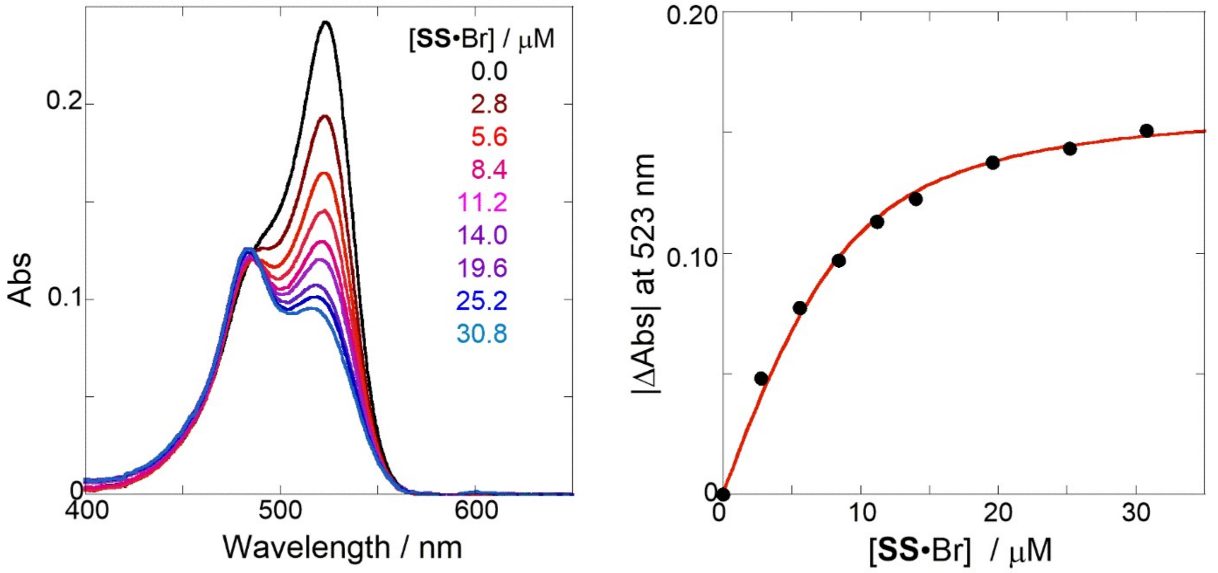
[SS•Br] / μM
0.0
2.8
5.6
8.4
11.2
14.0
19.6
25.2
30.8
Abs
0
0.1
0.2
400
500
600
Wavelength / nm
|ΔAbs| at 523 nm
0
0.10
0.20
0
10
20
30
[SS•Br] / μM

(f)

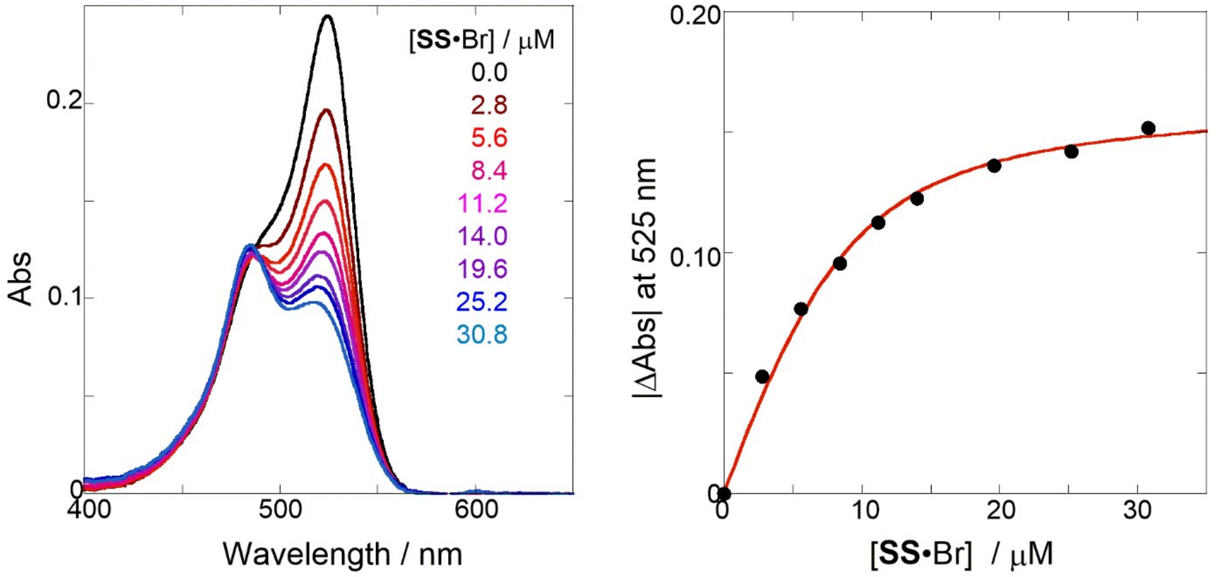
[SS•Br] / μM
0.0
2.8
5.6
8.4
11.2
14.0
19.6
25.2
30.8
Abs
0
0.1
0.2
400
500
600
Wavelength / nm
|ΔAbs| at 525 nm
0
0.10
0.20
0
10
20
30
[SS•Br] / μM

(g)

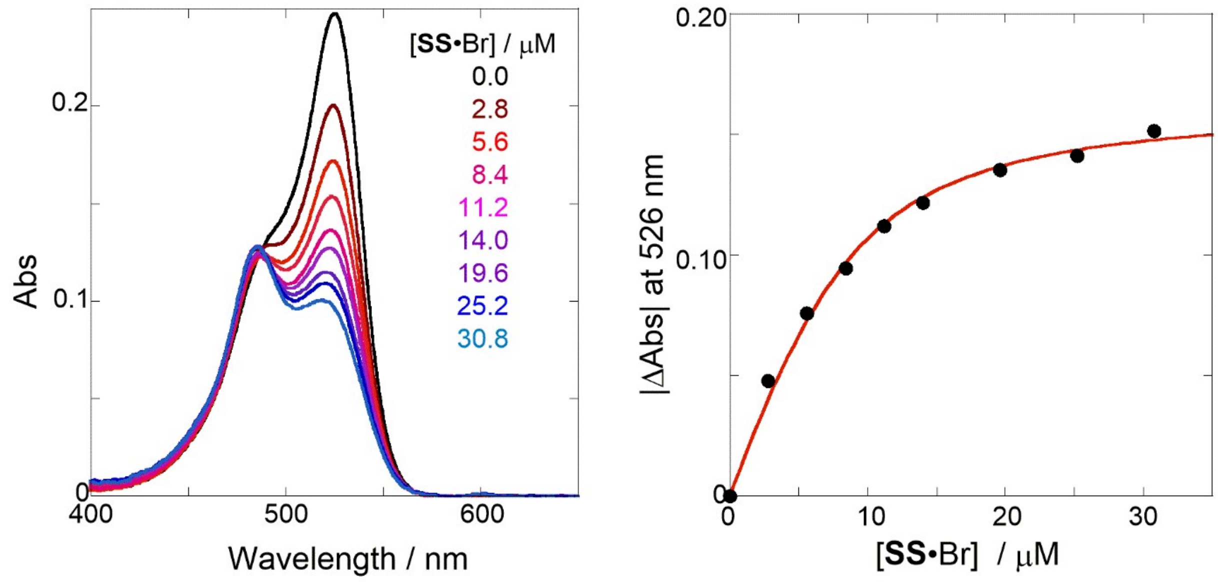


(h)

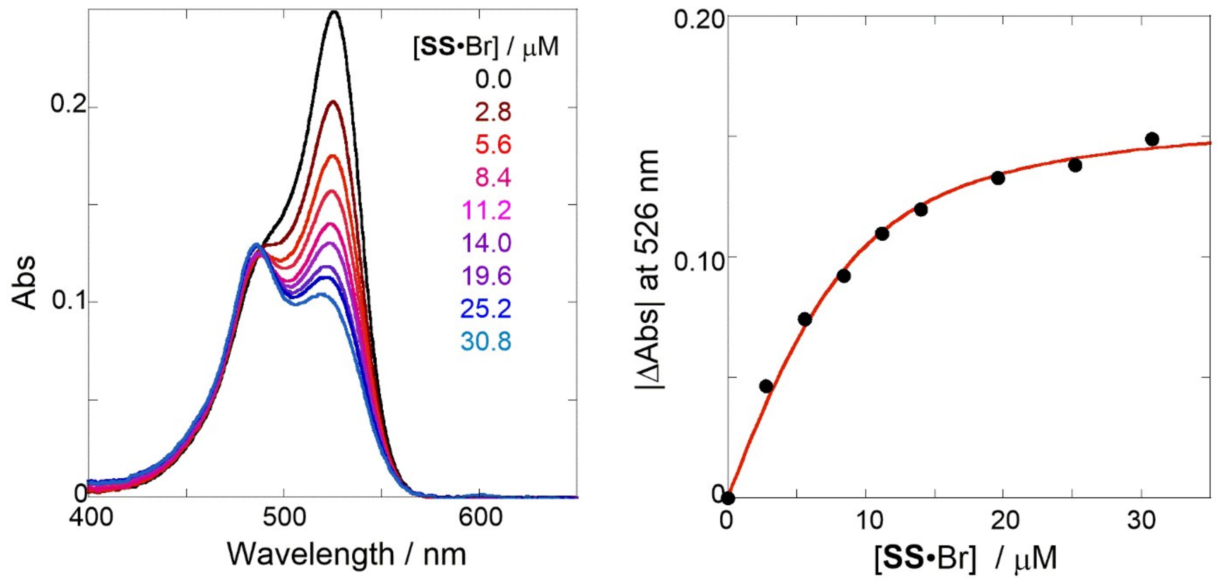


**Figure S10.** (*Left panels*) UV/vis absorption spectral changes of a chloroform solution of **H** (8.0 μM, black line) upon gradual addition of **SS**•Br (2.8–30.8 μM, colored line) and (*Right panels*) nonlinear least-squares fitting, assuming 1:1 stoichiometry of **SS**•Br with **H**, to determine the binding constant ($K_{\mathrm{anion}}$) at room temperature at (a) 40, (b) 80, (c) 120, (d) 160, (e) 200, (f) 240, (g) 280, and (h) 320 MPa, measured in a high-pressure cell.

**Table S2. Stoichiometric 1:1 Binding Constants ($K_{anion}$) and Apparent Reaction Volume Changes ($\Delta V^{\circ}_{T}$) for the Anion Receptor (H) with Some Anions in $CHCl_3$ under Hydrostatic Pressures[a]**

| Guest | $P$/MPa | $K$/$M^{-1}$ | $\Delta V^{\circ}_{T}$/$cm^3$ $mol^{-1}$ | Guest | $P$/MPa | $K$/$M^{-1}$ | $\Delta V^{\circ}_{T}$/$cm^3$ $mol^{-1}$ |
|---|---|---|---|---|---|---|---|
| TBA•Cl | 40 | $(2.65 \pm 0.28) \times 10^5$ | $-1.5 \pm 0.2$ | TBA•Br | 40 | $(1.77 \pm 0.14) \times 10^4$ | $-0.3 \pm 0.2$ |
| | 80 | $(2.69 \pm 0.27) \times 10^5$ | | | 80 | $(1.69 \pm 0.15) \times 10^4$ | |
| | 120 | $(2.70 \pm 0.29) \times 10^5$ | | | 120 | $(1.70 \pm 0.17) \times 10^4$ | |
| | 160 | $(2.74 \pm 0.29) \times 10^5$ | | | 160 | $(1.72 \pm 0.18) \times 10^4$ | |
| | 200 | $(2.85 \pm 0.31) \times 10^5$ | | | 200 | $(1.76 \pm 0.20) \times 10^4$ | |
| | 240 | $(2.87 \pm 0.31) \times 10^5$ | | | 240 | $(1.75 \pm 0.20) \times 10^4$ | |
| | 280 | $(3.04 \pm 0.39) \times 10^5$ | | | 280 | $(1.78 \pm 0.23) \times 10^4$ | |
| | 320 | $(3.12 \pm 0.39) \times 10^5$ | | | 320 | $(1.78 \pm 0.23) \times 10^4$ | |

[a] Measured at 298 K.

(a)

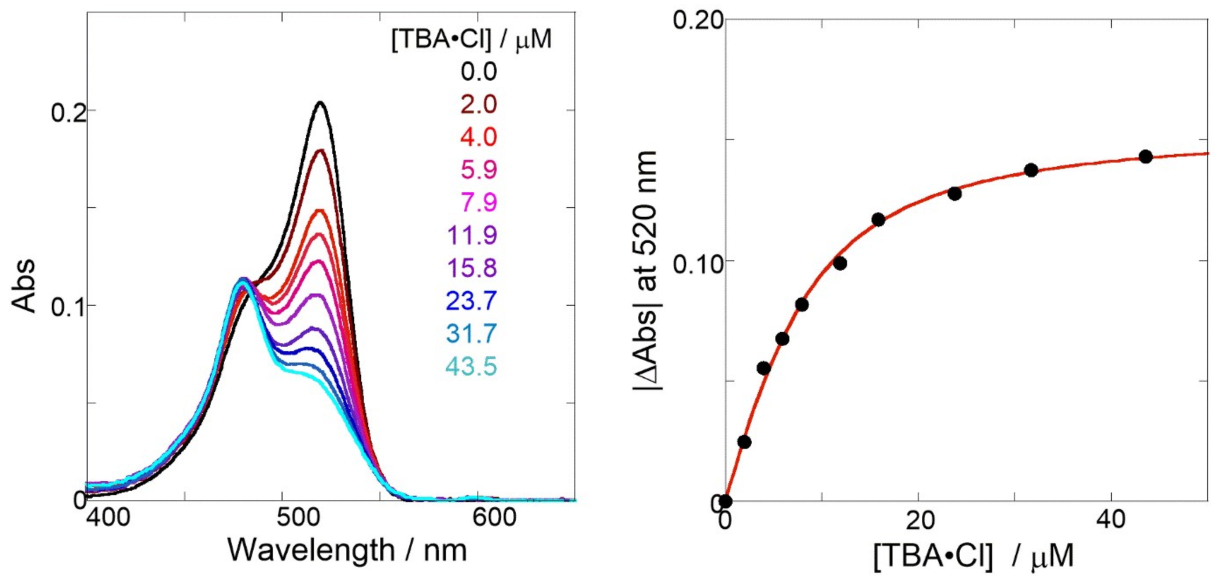
[TBA•Cl] / μM
0.0
2.0
4.0
5.9
7.9
11.9
15.8
23.7
31.7
43.5
Abs
Wavelength / nm
|ΔAbs| at 520 nm
[TBA•Cl] / μM

(b)

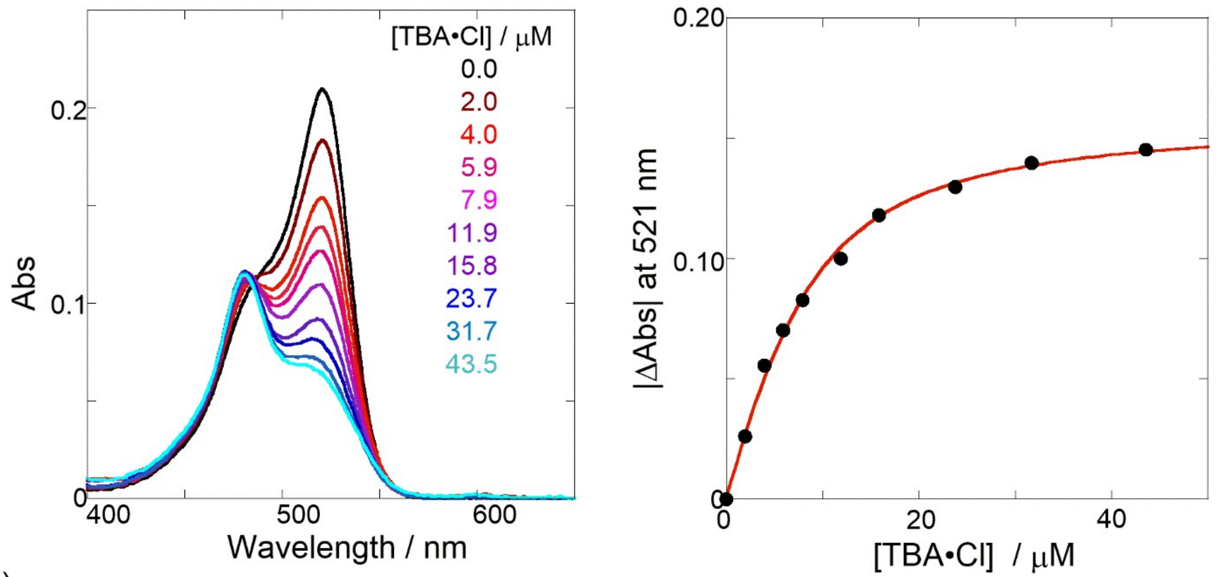
[TBA•Cl] / μM
0.0
2.0
4.0
5.9
7.9
11.9
15.8
23.7
31.7
43.5
Abs
Wavelength / nm
|ΔAbs| at 521 nm
[TBA•Cl] / μM

(c)

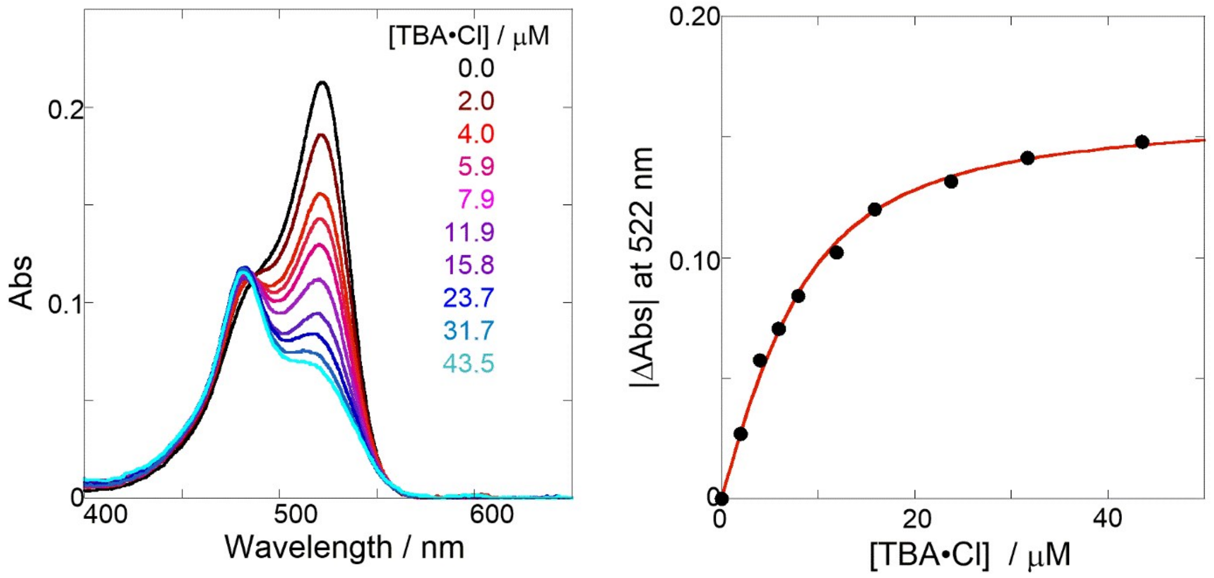
[TBA•Cl] / μM
0.0
2.0
4.0
5.9
7.9
11.9
15.8
23.7
31.7
43.5
Abs
Wavelength / nm
|ΔAbs| at 522 nm
[TBA•Cl] / μM

(d)

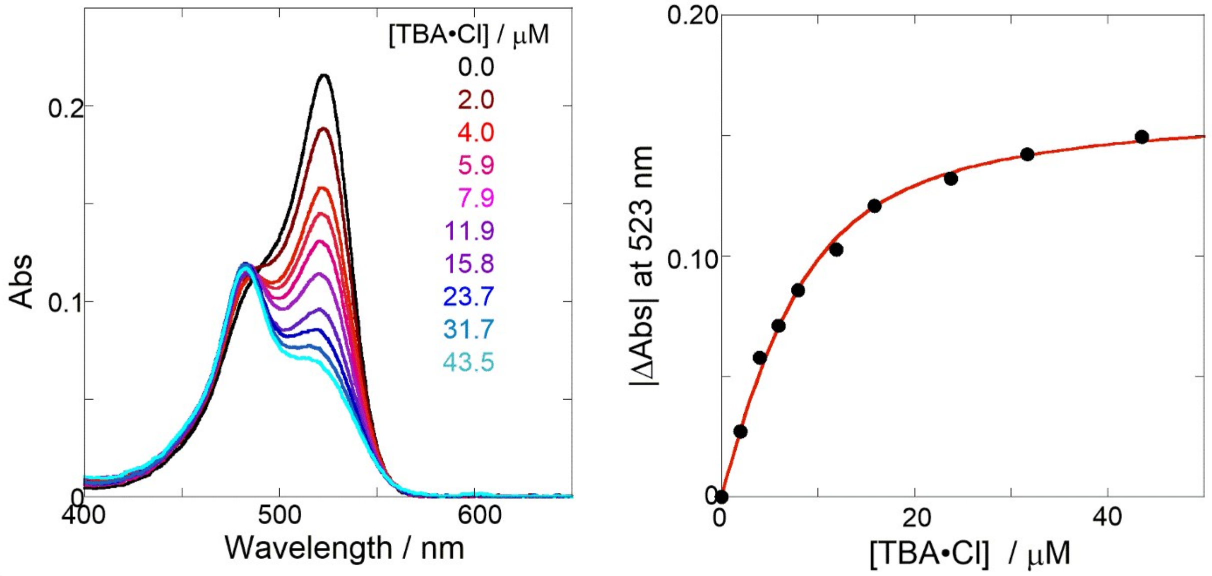

[TBA•Cl] / μM
0.0
2.0
4.0
5.9
7.9
11.9
15.8
23.7
31.7
43.5
Abs
0.2
0.1
0
400
500
600
Wavelength / nm
|ΔAbs| at 523 nm
0.20
0.10
0
20
40
[TBA•Cl] / μM


(e)

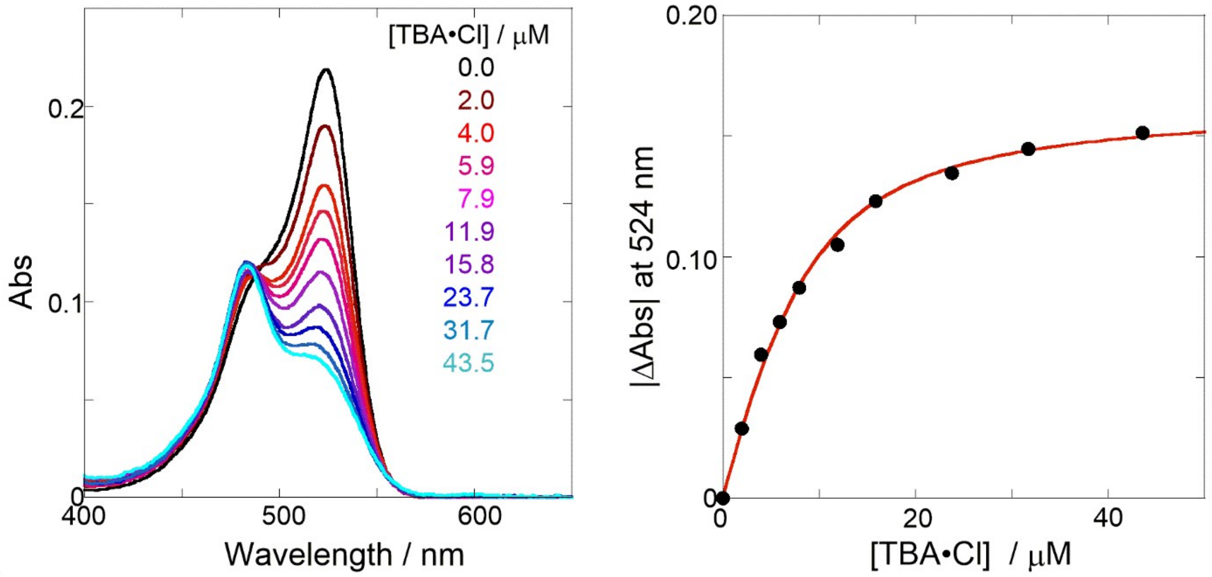

[TBA•Cl] / μM
0.0
2.0
4.0
5.9
7.9
11.9
15.8
23.7
31.7
43.5
Abs
0.2
0.1
0
400
500
600
Wavelength / nm
|ΔAbs| at 524 nm
0.20
0.10
0
20
40
[TBA•Cl] / μM


(f)

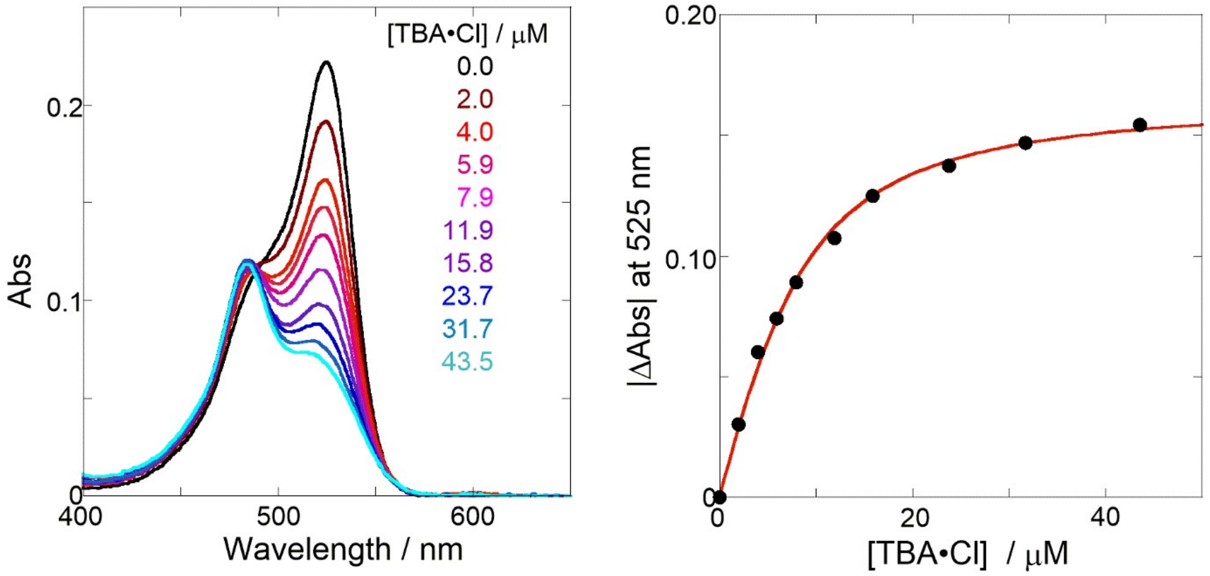

[TBA•Cl] / μM
0.0
2.0
4.0
5.9
7.9
11.9
15.8
23.7
31.7
43.5
Abs
0.2
0.1
0
400
500
600
Wavelength / nm
|ΔAbs| at 525 nm
0.20
0.10
0
20
40
[TBA•Cl] / μM

(g)

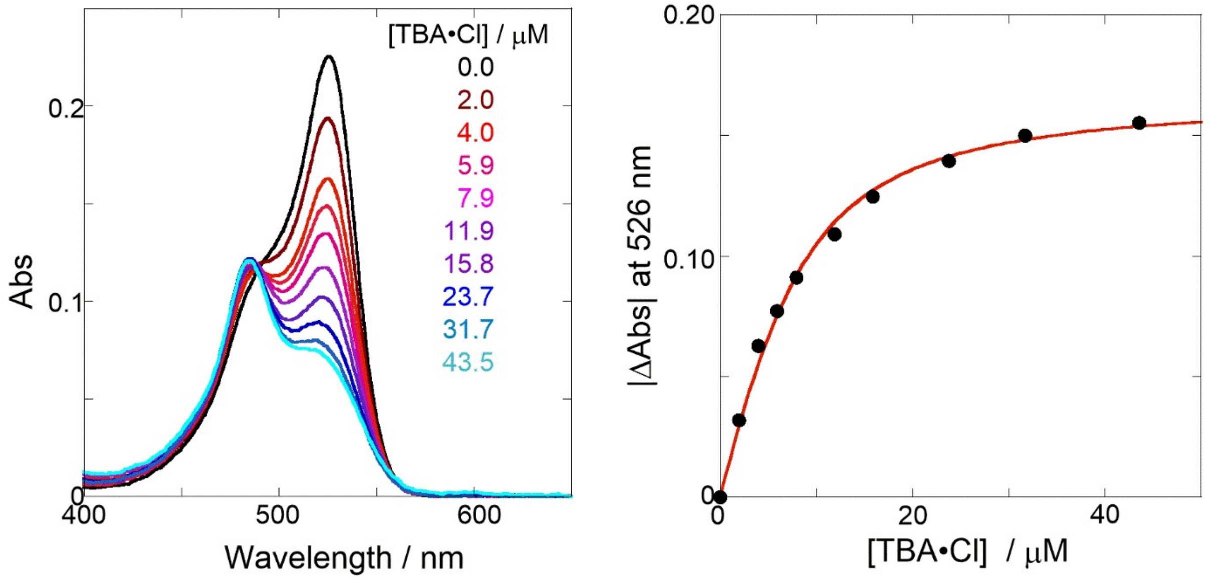


(h)

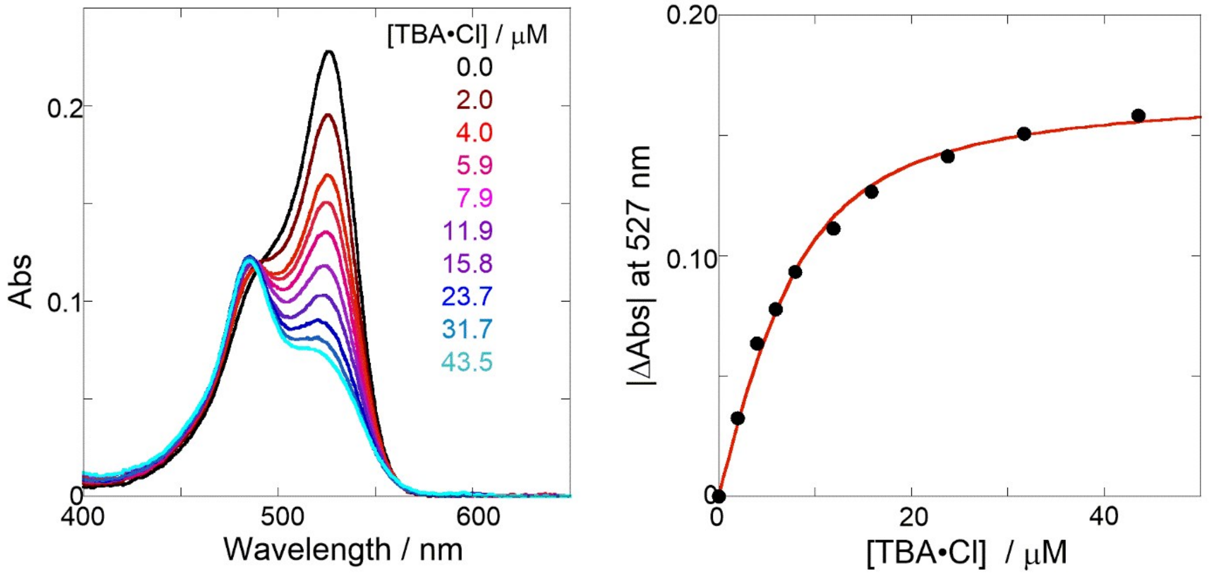


**Figure S11.** (*Left panels*) UV/vis absorption spectral changes of a chloroform solution of **H** (8.0 μM, black line) upon gradual addition of TBA•Cl (2.0–43.5 μM, colored line) and (*Right panels*) nonlinear least-squares fitting, assuming 1:1 stoichiometry of TBA•Cl with **H**, to determine the binding constant ($K_{\text{anion}}$) at room temperature at (a) 40, (b) 80, (c) 120, (d) 160, (e) 200, (f) 240, (g) 280, and (h) 320 MPa, measured in a high-pressure cell.

(a)

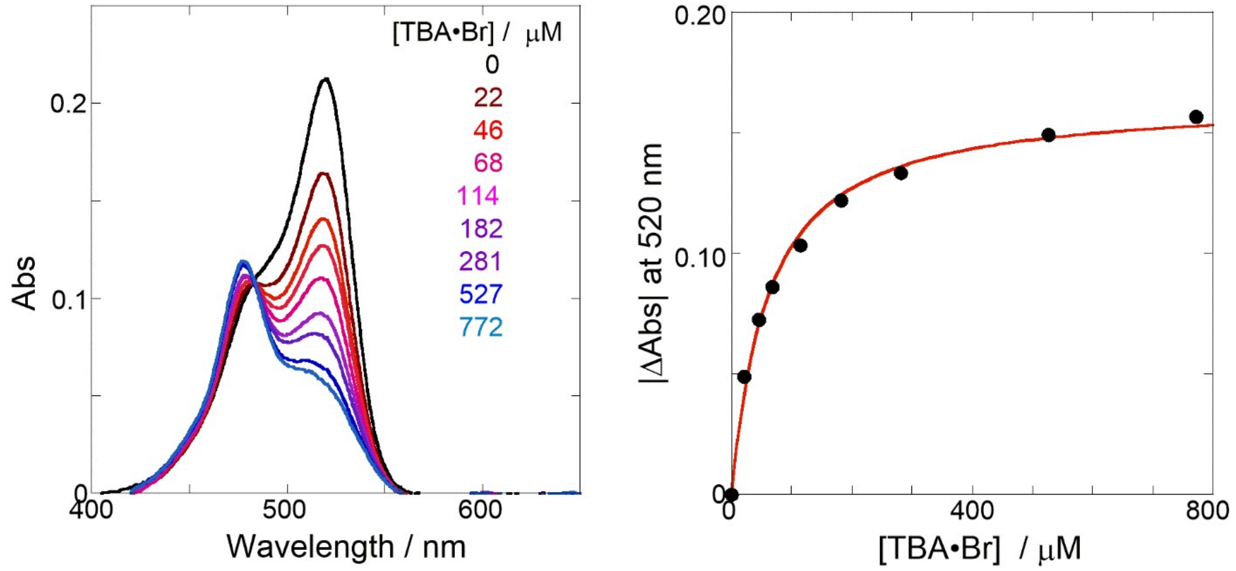
[TBA•Br] / μM
0
22
46
68
114
182
281
527
772
Abs
Wavelength / nm
|ΔAbs| at 520 nm
[TBA•Br] / μM

(b)

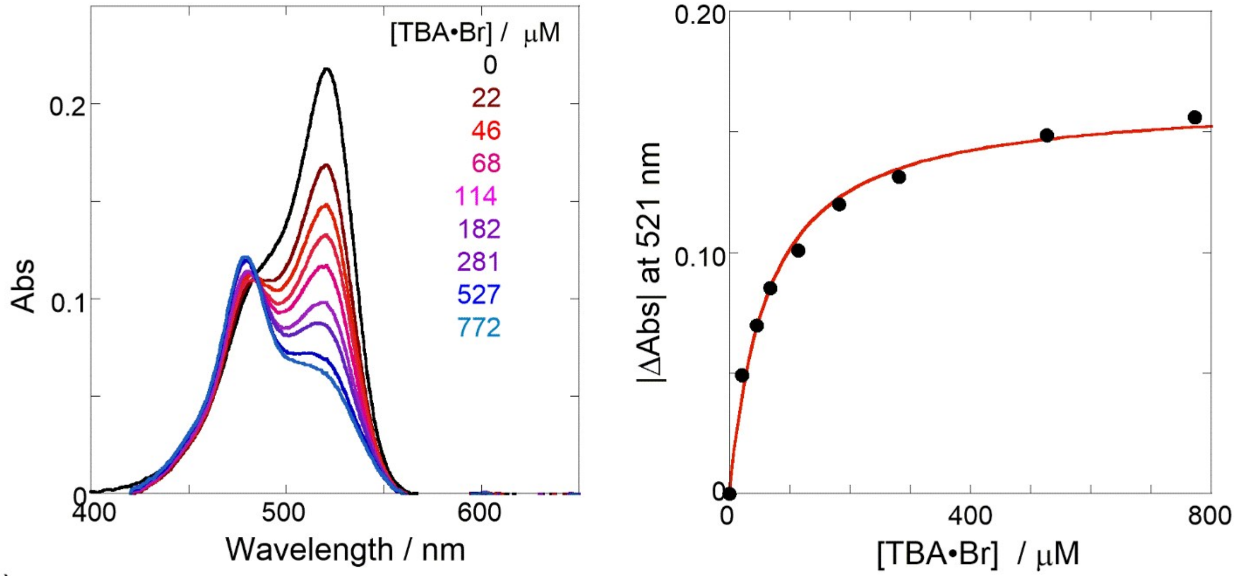
[TBA•Br] / μM
0
22
46
68
114
182
281
527
772
Abs
Wavelength / nm
|ΔAbs| at 521 nm
[TBA•Br] / μM

(c)

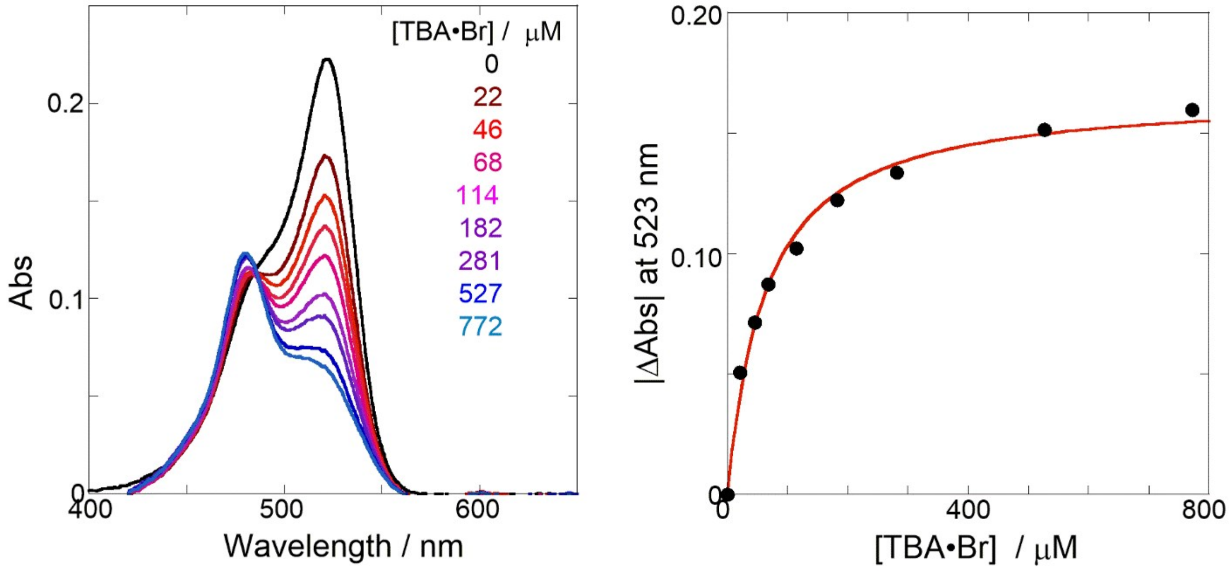
[TBA•Br] / μM
0
22
46
68
114
182
281
527
772
Abs
Wavelength / nm
|ΔAbs| at 523 nm
[TBA•Br] / μM

(d)

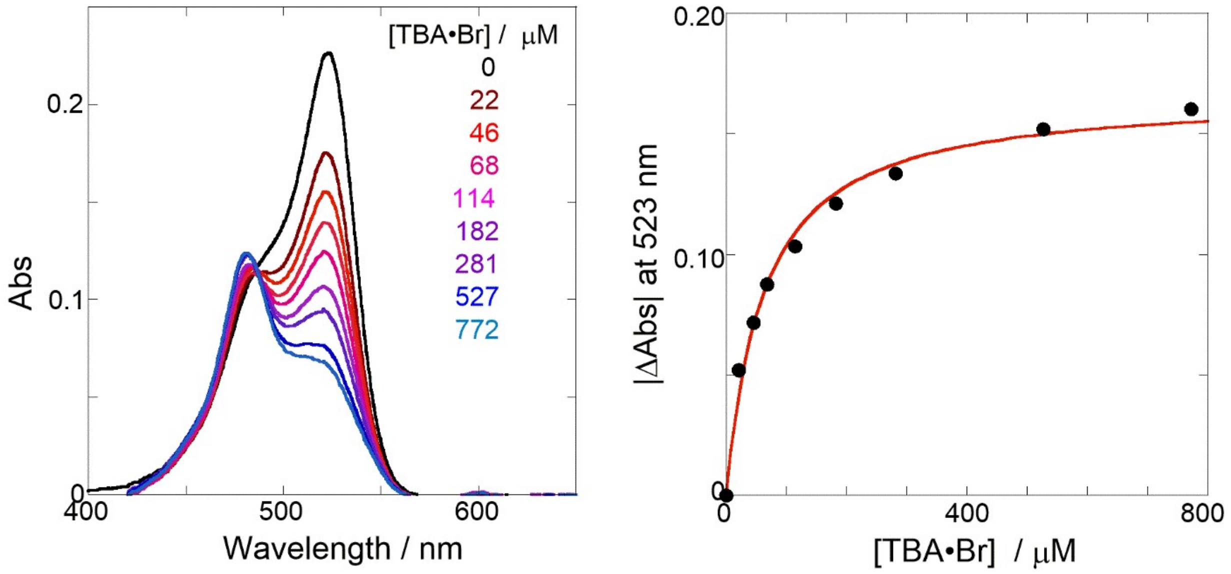
[TBA•Br] / μM
0
22
46
68
114
182
281
527
772
Abs
0.2
0.1
0
400
500
600
Wavelength / nm
|ΔAbs| at 523 nm
0.20
0.10
0
400
800
[TBA•Br] / μM

(e)

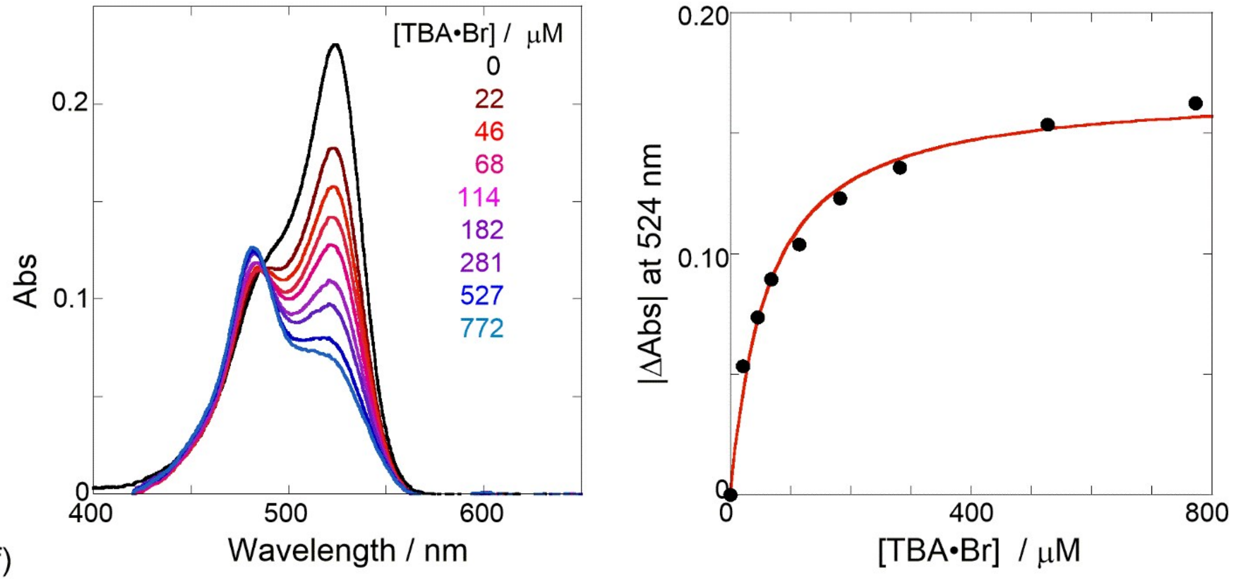
[TBA•Br] / μM
0
22
46
68
114
182
281
527
772
Abs
0.2
0.1
0
400
500
600
Wavelength / nm
|ΔAbs| at 524 nm
0.20
0.10
0
400
800
[TBA•Br] / μM

(f)

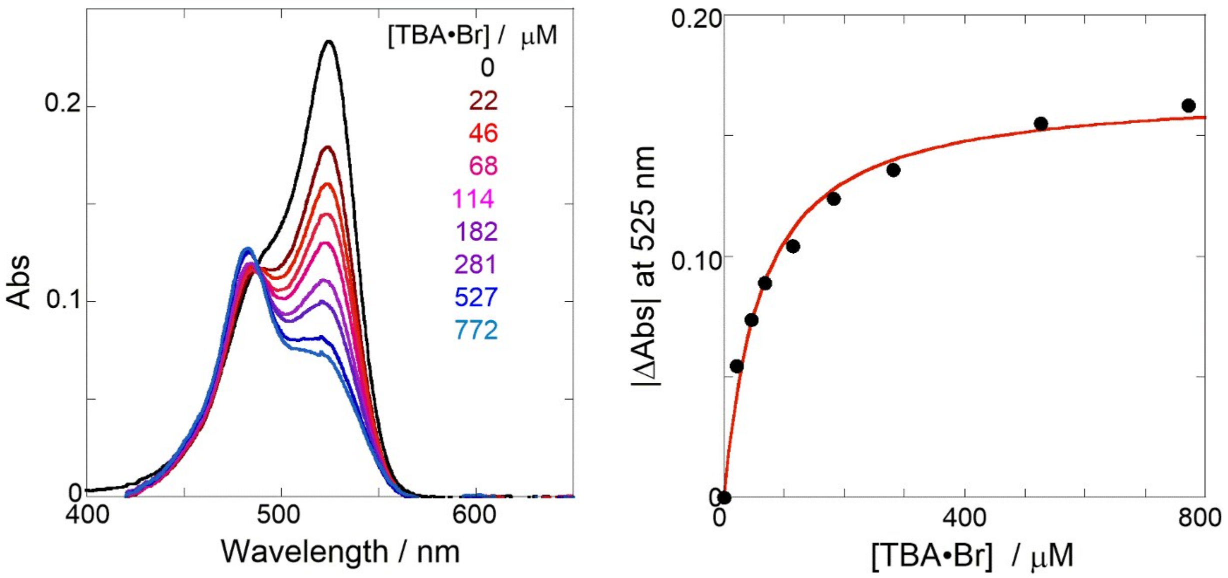
[TBA•Br] / μM
0
22
46
68
114
182
281
527
772
Abs
0.2
0.1
0
400
500
600
Wavelength / nm
|ΔAbs| at 525 nm
0.20
0.10
0
400
800
[TBA•Br] / μM

(g)

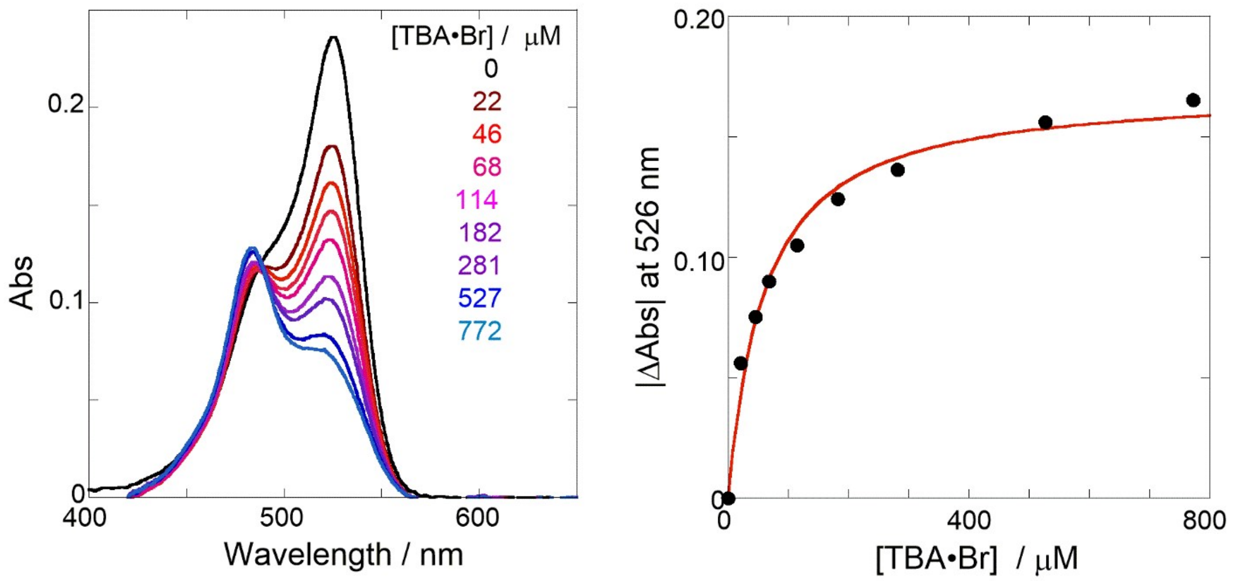


(h)

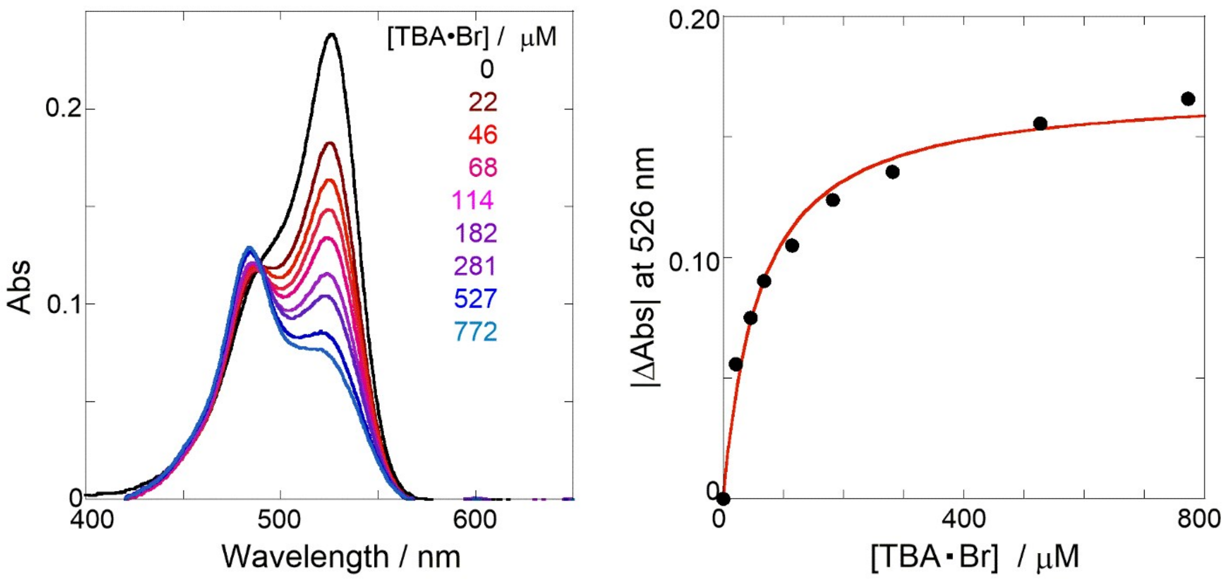


**Figure S12.** (*Left panels*) UV/vis absorption spectral changes of a chloroform solution of **H** (7.5 μM, black line) upon gradual addition of TBA•Br (22–772 μM, colored line) and (*Right panels*) nonlinear least-squares fitting, assuming 1:1 stoichiometry of TBA•Br with **H**, to determine the binding constant ($K_{anion}$) at room temperature at (a) 40, (b) 80, (c) 120, (d) 160, (e) 200, (f) 240, (g) 280, and (h) 320 MPa, measured in a high-pressure cell.

**Table S3. Stoichiometric 1:1 Binding Constants ($K_{anion}$) and Apparent Reaction Volume Changes ($\Delta V^{\circ}_{T}$) for the Anion Receptor (H) with Some Anions in $CHCl_3$ Containing MeOH (0–0.3%) under Hydrostatic Pressures[a]**

| Guest | $P$/MPa | Methanol/% | $K$/$M^{-1}$ | $\Delta V^{\circ}_{T}$/$cm^3\ mol^{-1}$ | Guest | $P$/MPa | Methanol/% | $K$/$M^{-1}$ | $\Delta V^{\circ}_{T}$/$cm^3\ mol^{-1}$ |
|---|---|---|---|---|---|---|---|---|---|
| **RR**•Cl | 40 | 0 | $(6.28 \pm 4.17) \times 10^6$ | +9.1 ± 0.8 | **SS**•Br | 40 | 0 | $(5.19 \pm 0.56) \times 10^5$ | +2.6 ± 0.3 |
| | 80 | | $(4.97 \pm 3.41) \times 10^6$ | | | 80 | | $(4.88 \pm 0.56) \times 10^5$ | |
| | 120 | | $(3.81 \pm 2.14) \times 10^6$ | | | 120 | | $(4.43 \pm 0.62) \times 10^5$ | |
| | 160 | | $(3.36 \pm 1.80) \times 10^6$ | | | 160 | | $(4.20 \pm 0.70) \times 10^5$ | |
| | 200 | | $(2.93 \pm 1.51) \times 10^6$ | | | 200 | | $(4.18 \pm 0.68) \times 10^5$ | |
| | 240 | | $(2.74 \pm 1.34) \times 10^6$ | | | 240 | | $(4.04 \pm 0.75) \times 10^5$ | |
| | 280 | | $(2.50 \pm 1.15) \times 10^6$ | | | 280 | | $(3.92 \pm 0.70) \times 10^5$ | |
| | 320 | | $(2.08 \pm 0.91) \times 10^6$ | | | 320 | | $(3.82 \pm 0.67) \times 10^5$ | |
| **RR**•Cl | 40 | 0.1 | $(4.02 \pm 3.94) \times 10^6$ | +9.7 ± 1.2 | **SS**•Br | 40 | 0.1 | $(6.77 \pm 1.36) \times 10^5$ | +2.3 ± 0.6 |
| | 80 | | $(2.68 \pm 2.11) \times 10^6$ | | | 80 | | $(7.20 \pm 1.06) \times 10^5$ | |
| | 120 | | $(2.19 \pm 1.57) \times 10^6$ | | | 120 | | $(7.18 \pm 1.42) \times 10^5$ | |
| | 160 | | $(1.75 \pm 1.27) \times 10^6$ | | | 160 | | $(7.06 \pm 1.26) \times 10^5$ | |
| | 200 | | $(1.63 \pm 0.93) \times 10^6$ | | | 200 | | $(6.06 \pm 1.00) \times 10^5$ | |
| | 240 | | $(1.60 \pm 1.02) \times 10^6$ | | | 240 | | $(6.39 \pm 1.15) \times 10^5$ | |
| | 280 | | $(1.33 \pm 0.76) \times 10^6$ | | | 280 | | $(5.86 \pm 1.00) \times 10^5$ | |
| | 320 | | $(1.18 \pm 0.58) \times 10^6$ | | | 320 | | $(5.42 \pm 0.89) \times 10^5$ | |
| **RR**•Cl | 40 | 0.2 | $(2.05 \pm 0.96) \times 10^6$ | +8.5 ± 0.6 | **SS**•Br | 40 | 0.2 | $(3.52 \pm 0.55) \times 10^5$ | +3.4 ± 0.1 |
| | 80 | | $(1.70 \pm 0.75) \times 10^6$ | | | 80 | | $(3.30 \pm 0.61) \times 10^5$ | |
| | 120 | | $(1.33 \pm 0.54) \times 10^6$ | | | 120 | | $(3.04 \pm 0.64) \times 10^5$ | |
| | 160 | | $(1.19 \pm 0.45) \times 10^6$ | | | 160 | | $(2.99 \pm 0.68) \times 10^5$ | |
| | 200 | | $(1.07 \pm 0.39) \times 10^6$ | | | 200 | | $(2.79 \pm 0.70) \times 10^5$ | |
| | 240 | | $(0.93 \pm 0.32) \times 10^6$ | | | 240 | | $(2.69 \pm 0.68) \times 10^5$ | |
| | 280 | | $(0.85 \pm 0.28) \times 10^6$ | | | 280 | | $(2.53 \pm 0.68) \times 10^5$ | |
| | 320 | | $(0.77 \pm 0.25) \times 10^6$ | | | 320 | | $(2.34 \pm 0.69) \times 10^5$ | |
| **RR**•Cl | 40 | 0.3 | $(1.71 \pm 0.66) \times 10^6$ | +9.3 ± 1.1 | **SS**•Br | 40 | 0.3 | $(1.61 \pm 0.20) \times 10^5$ | +3.5 ± 0.8 |
| | 80 | | $(1.13 \pm 0.39) \times 10^6$ | | | 80 | | $(1.43 \pm 0.25) \times 10^5$ | |
| | 120 | | $(0.93 \pm 0.28) \times 10^6$ | | | 120 | | $(1.18 \pm 0.16) \times 10^5$ | |
| | 160 | | $(0.81 \pm 0.23) \times 10^6$ | | | 160 | | $(1.17 \pm 0.21) \times 10^5$ | |
| | 200 | | $(0.70 \pm 0.19) \times 10^6$ | | | 200 | | $(1.05 \pm 0.21) \times 10^5$ | |
| | 240 | | $(0.65 \pm 0.18) \times 10^6$ | | | 240 | | $(1.17 \pm 0.27) \times 10^5$ | |
| | 280 | | $(0.60 \pm 0.15) \times 10^6$ | | | 280 | | $(1.04 \pm 0.25) \times 10^5$ | |
| | 320 | | $(0.53 \pm 0.12) \times 10^6$ | | | 320 | | $(1.05 \pm 0.30) \times 10^5$ | |

[a] Measured at 298 K.

(a)

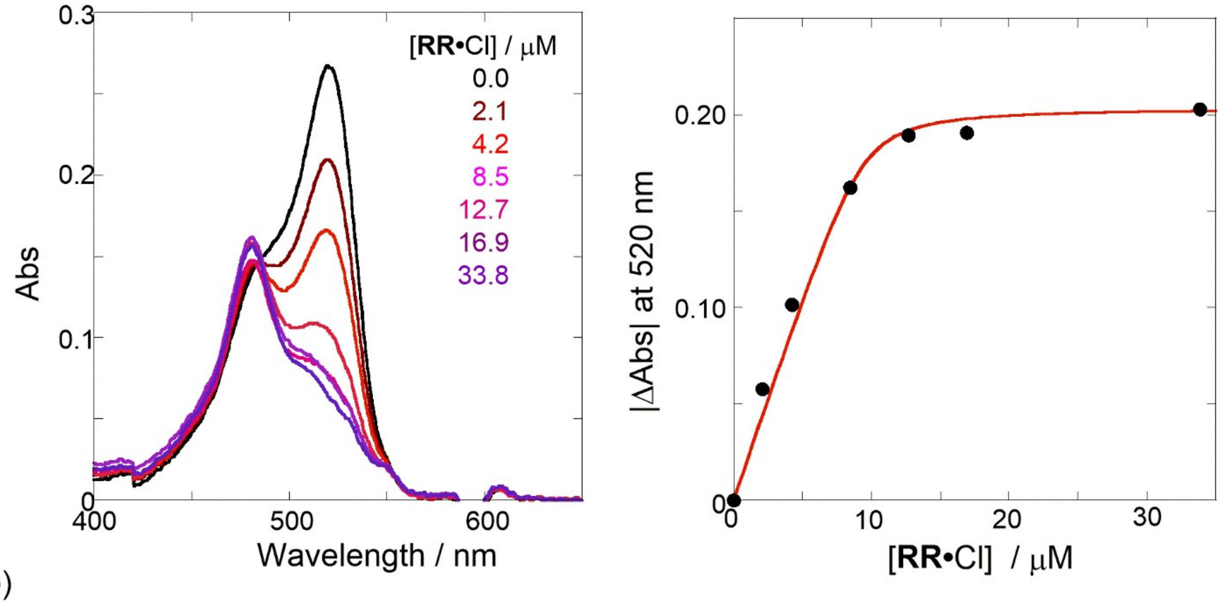

[RR•Cl] / μM
0.0
2.1
4.2
8.5
12.7
16.9
33.8
Abs
Wavelength / nm
|ΔAbs| at 520 nm
[RR•Cl] / μM


(b)

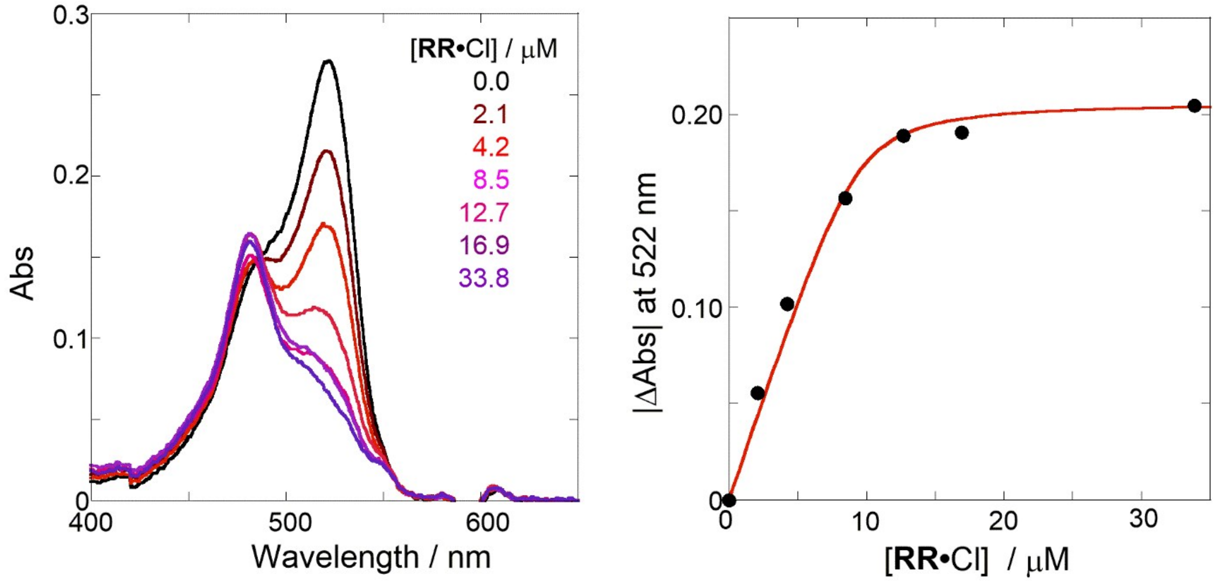

[RR•Cl] / μM
0.0
2.1
4.2
8.5
12.7
16.9
33.8
Abs
Wavelength / nm
|ΔAbs| at 522 nm
[RR•Cl] / μM


(c)

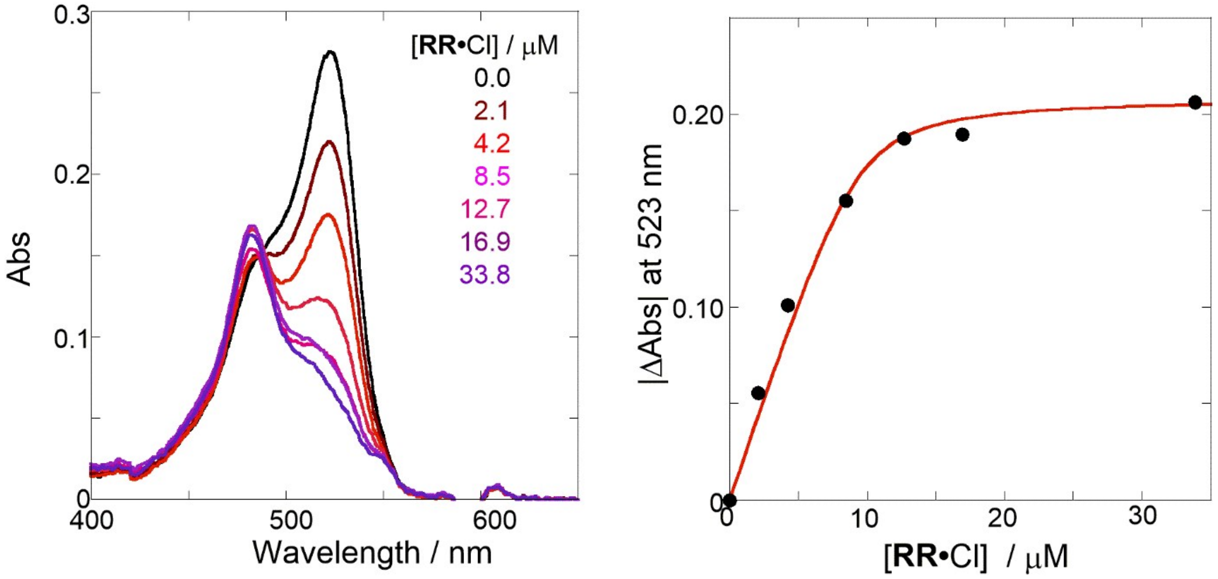

[RR•Cl] / μM
0.0
2.1
4.2
8.5
12.7
16.9
33.8
Abs
Wavelength / nm
|ΔAbs| at 523 nm
[RR•Cl] / μM

(d)

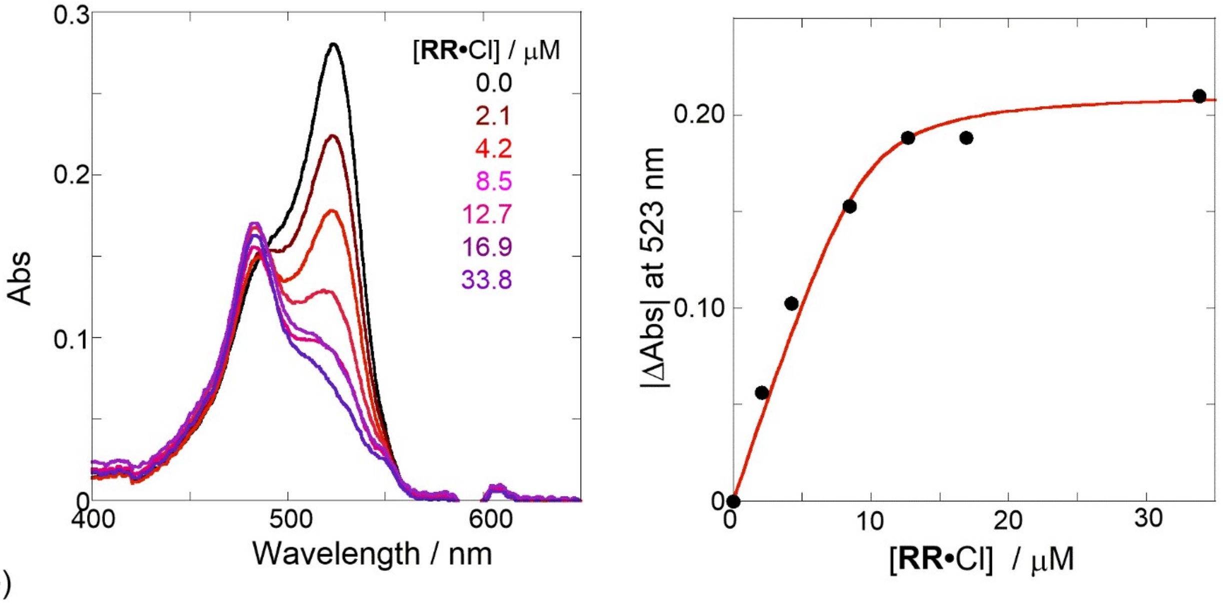

Abs
Wavelength / nm
[RR•Cl] / μM
0.0
2.1
4.2
8.5
12.7
16.9
33.8
|ΔAbs| at 523 nm
[RR•Cl] / μM


(e)

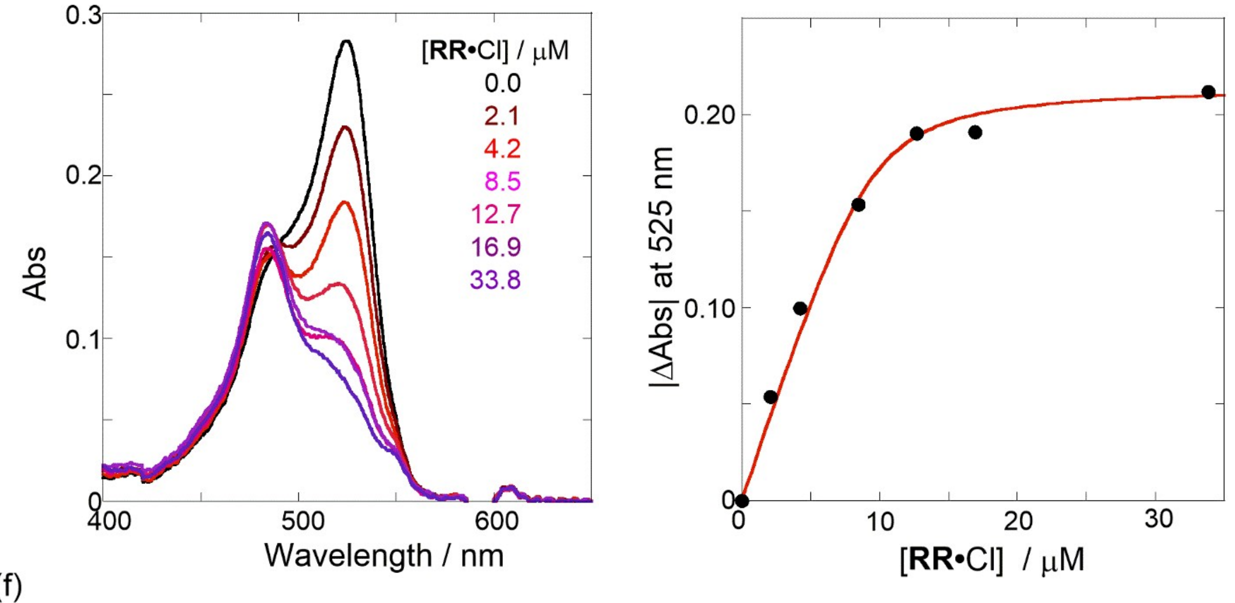

Abs
Wavelength / nm
[RR•Cl] / μM
0.0
2.1
4.2
8.5
12.7
16.9
33.8
|ΔAbs| at 525 nm
[RR•Cl] / μM


(f)

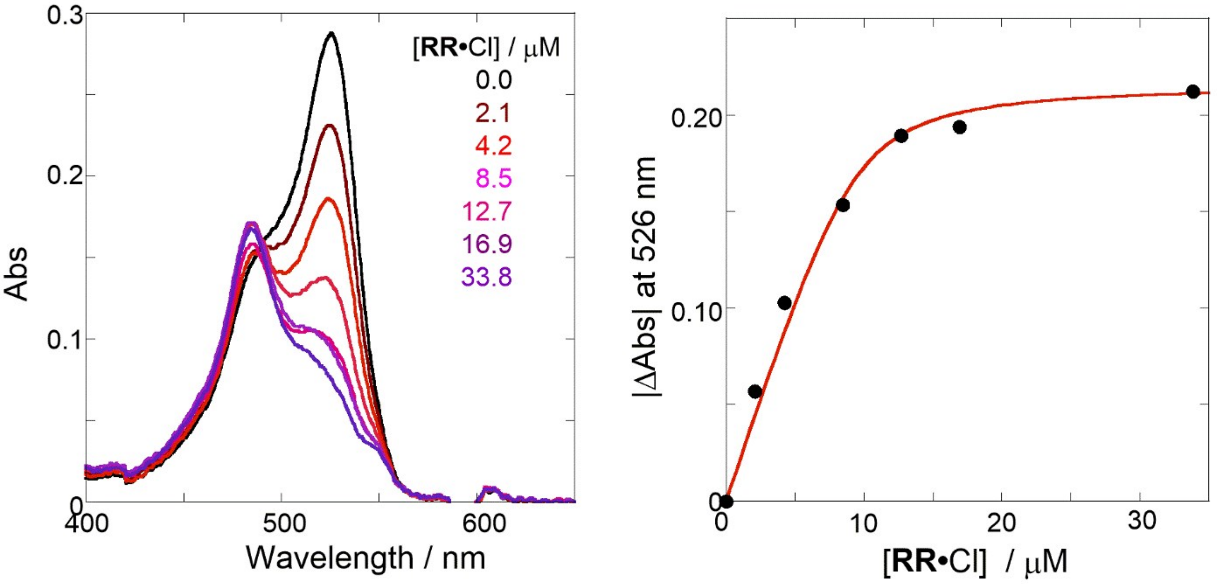

Abs
Wavelength / nm
[RR•Cl] / μM
0.0
2.1
4.2
8.5
12.7
16.9
33.8
|ΔAbs| at 526 nm
[RR•Cl] / μM

(g)

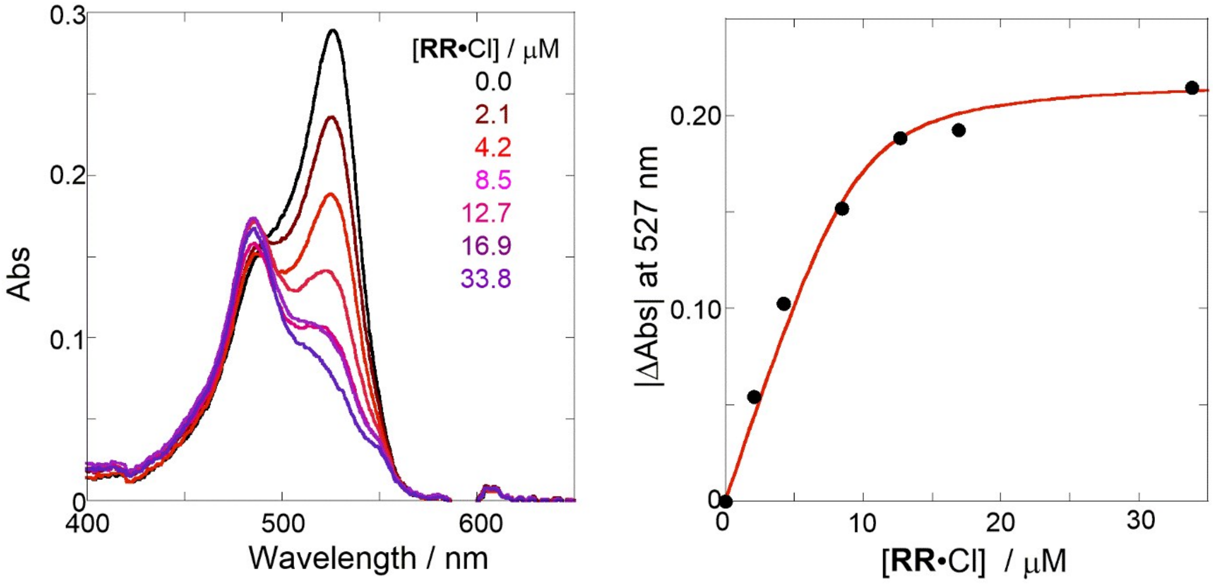


(h)

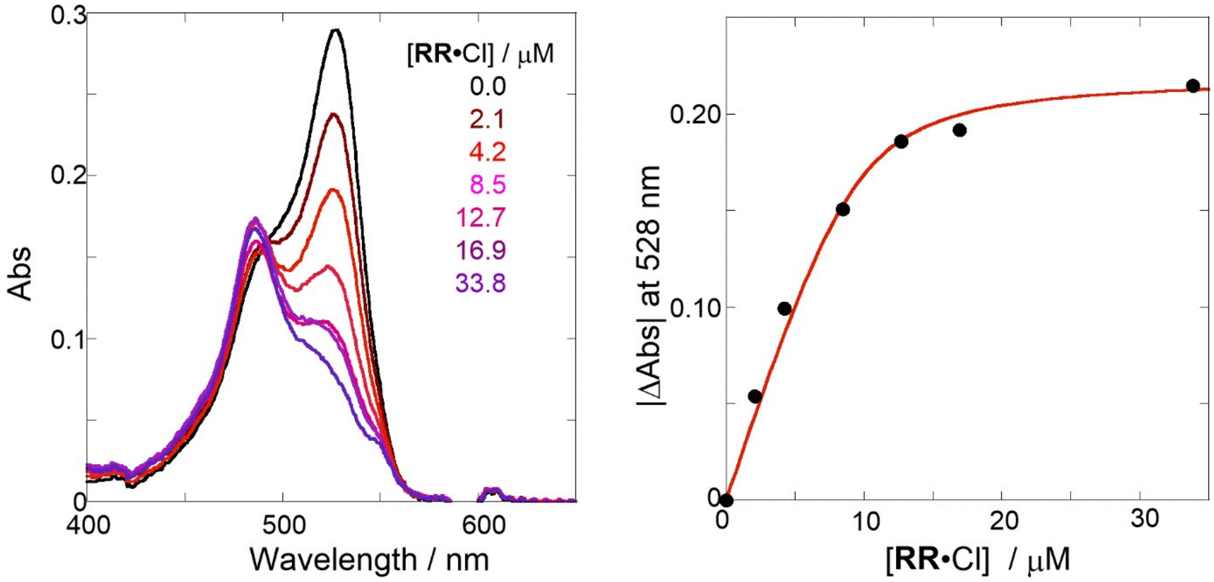


**Figure S13.** (*Left panels*) UV/vis absorption spectral changes of a chloroform solution containing methanol (0.1 vol%) of **H** (9.4 μM) upon gradual addition of **RR**•Cl (2.1–33.8 μM, colored line) and (*Right panels*) nonlinear least-squares fitting, assuming 1:1 stoichiometry of **RR**•Cl with **H**, to determine the binding constant ($K_{anion}$) at room temperature at (a) 40, (b) 80, (c) 120, (d) 160, (e) 200, (f) 240, (g) 280, and (h) 320 MPa, measured in a high-pressure cell.

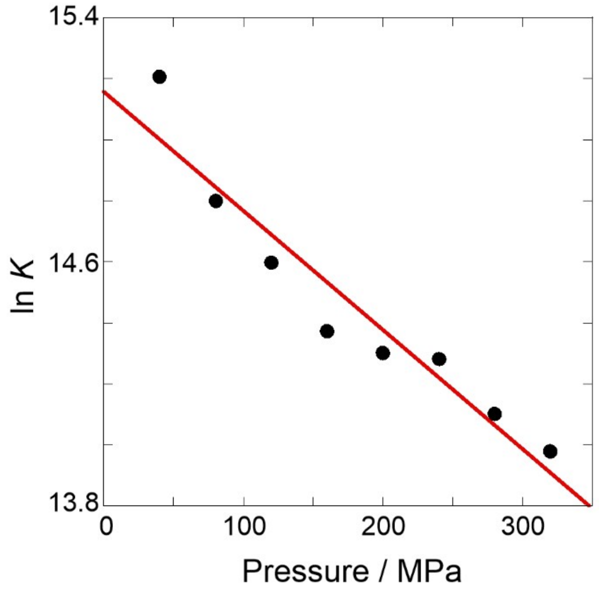


**Figure S14.** Pressure dependence of binding constant ($K_{anion}$) in anion recognition of **H** with **RR**•Cl in chloroform containing methanol (0.1 vol%) at room temperature.

(a)

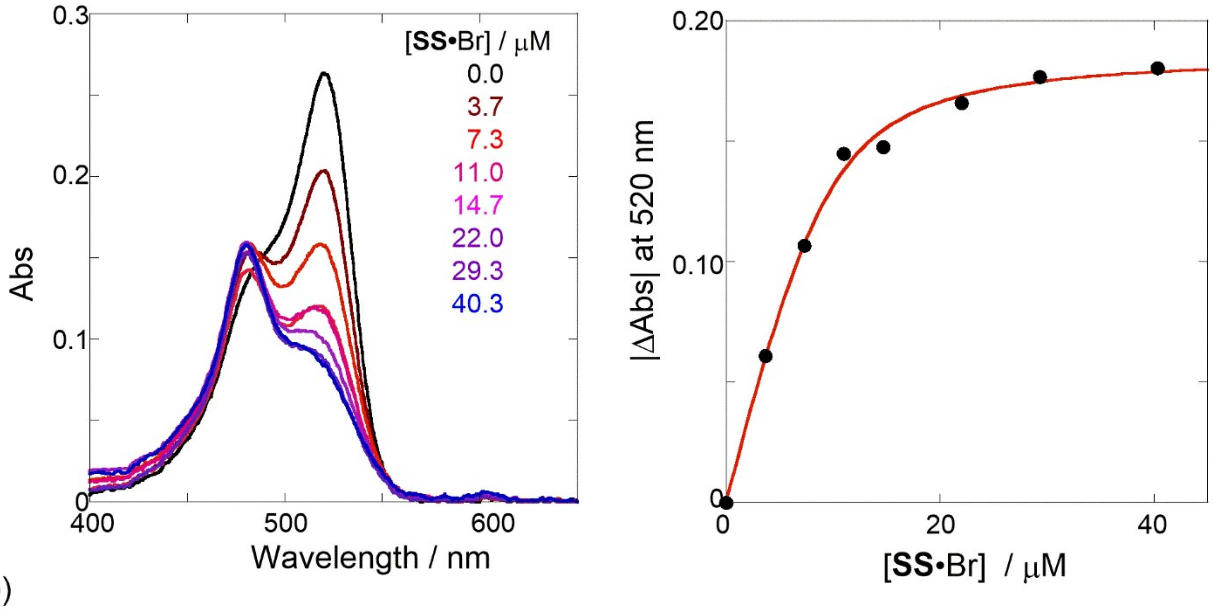
[SS•Br] / μM
0.0
3.7
7.3
11.0
14.7
22.0
29.3
40.3
Abs
Wavelength / nm
|ΔAbs| at 520 nm
[SS•Br] / μM

(b)

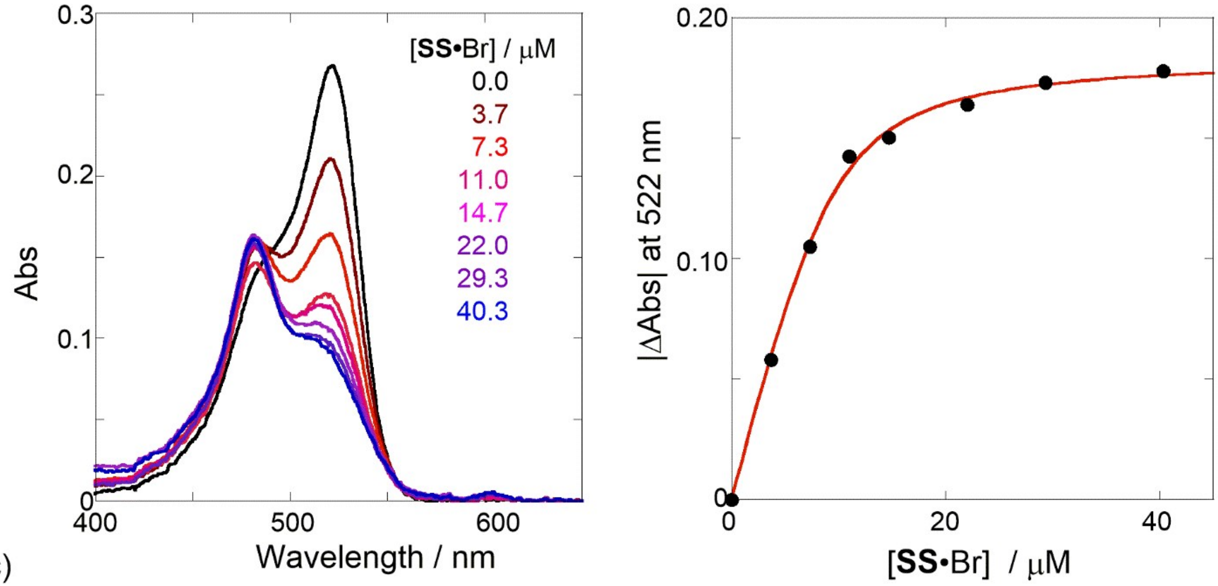
[SS•Br] / μM
0.0
3.7
7.3
11.0
14.7
22.0
29.3
40.3
Abs
Wavelength / nm
|ΔAbs| at 522 nm
[SS•Br] / μM

(c)

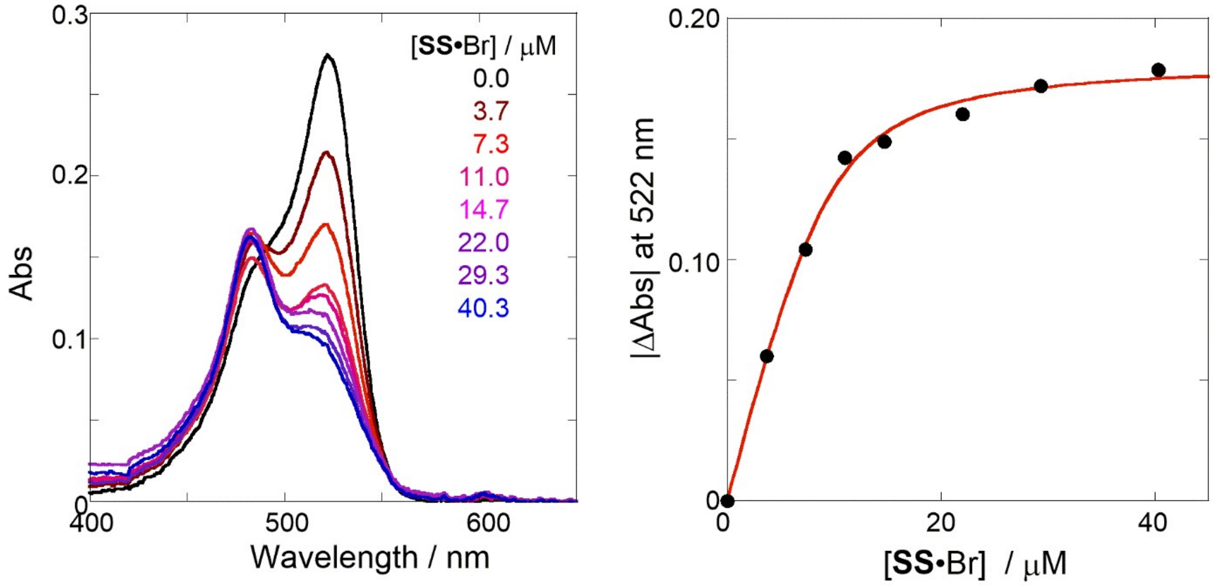
[SS•Br] / μM
0.0
3.7
7.3
11.0
14.7
22.0
29.3
40.3
Abs
Wavelength / nm
|ΔAbs| at 522 nm
[SS•Br] / μM

(d)

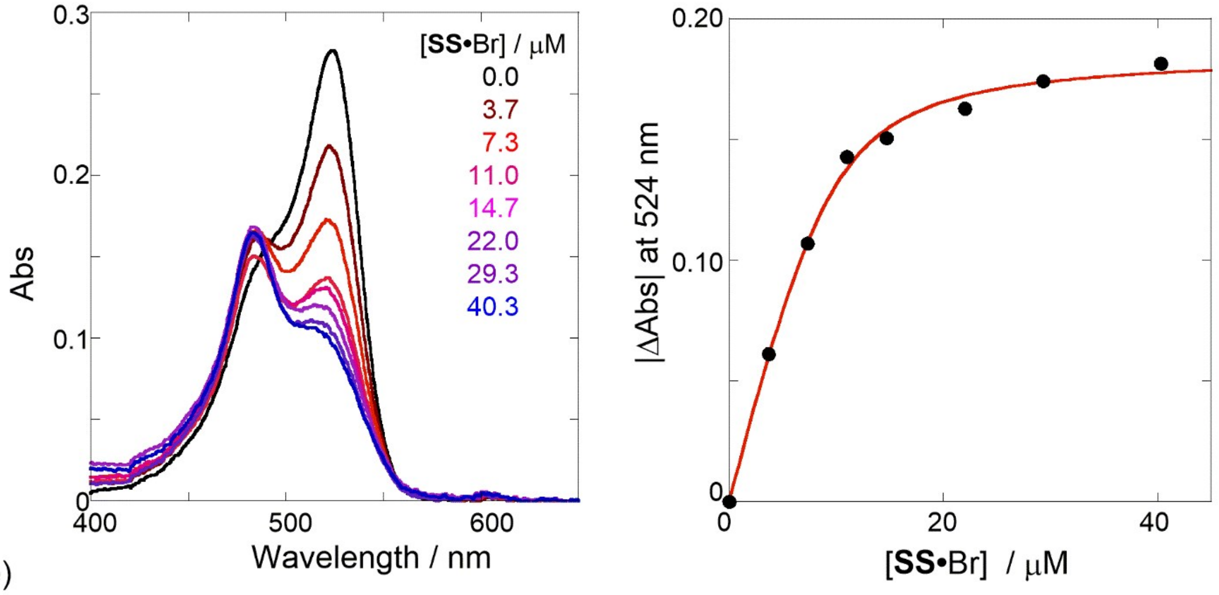
[SS•Br] / μM
0.0
3.7
7.3
11.0
14.7
22.0
29.3
40.3
Abs
Wavelength / nm
|ΔAbs| at 524 nm
[SS•Br] / μM

(e)

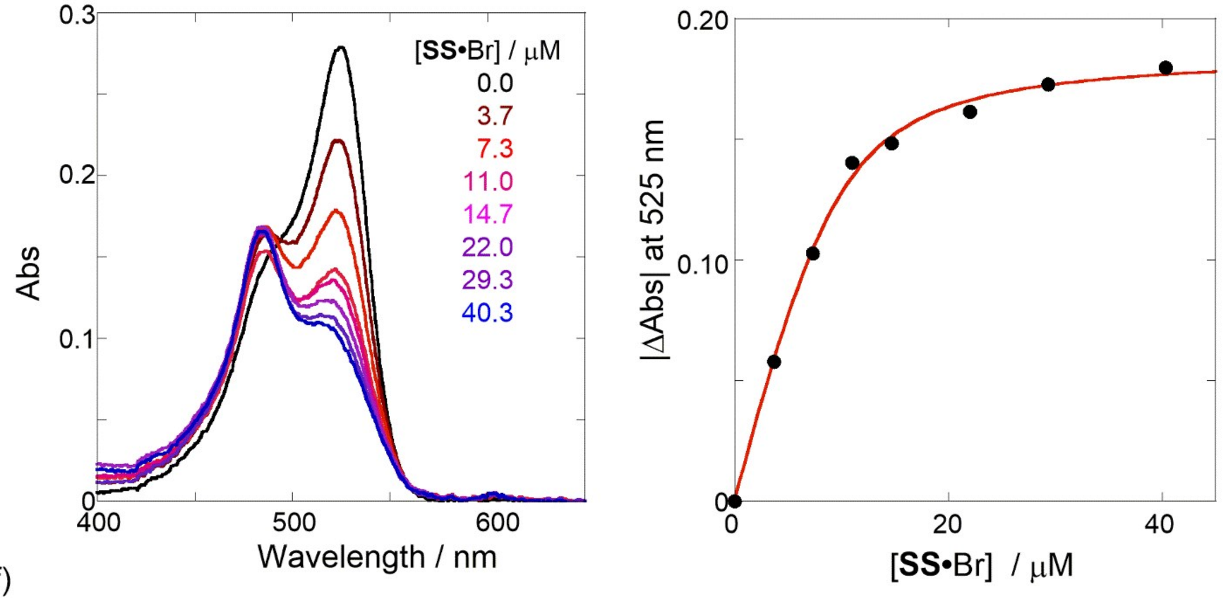
[SS•Br] / μM
0.0
3.7
7.3
11.0
14.7
22.0
29.3
40.3
Abs
Wavelength / nm
|ΔAbs| at 525 nm
[SS•Br] / μM

(f)

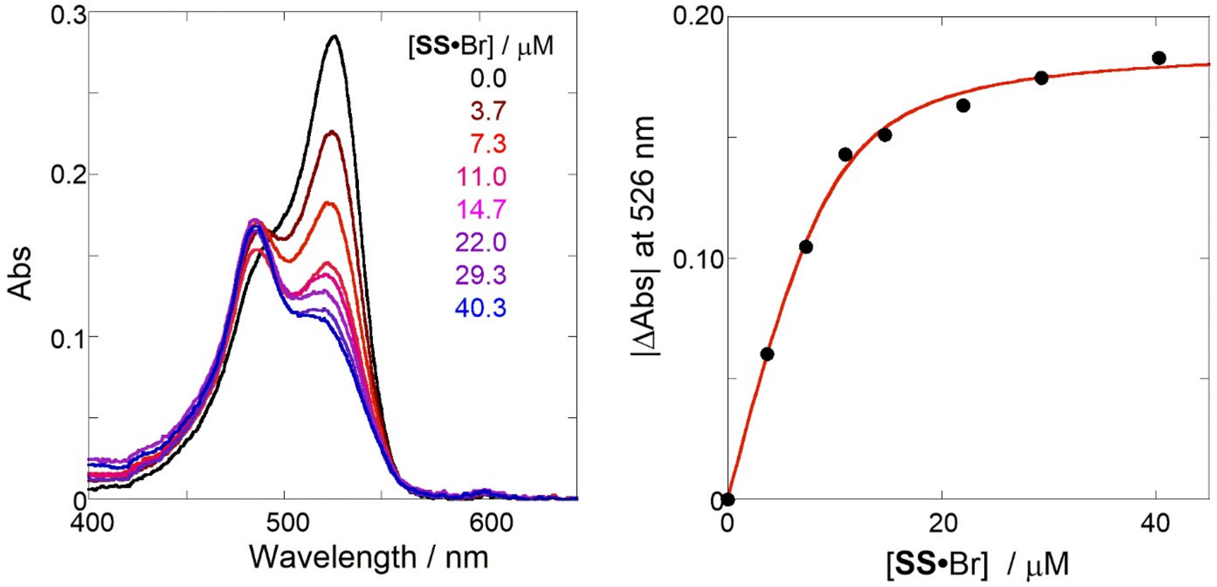
[SS•Br] / μM
0.0
3.7
7.3
11.0
14.7
22.0
29.3
40.3
Abs
Wavelength / nm
|ΔAbs| at 526 nm
[SS•Br] / μM

(g)

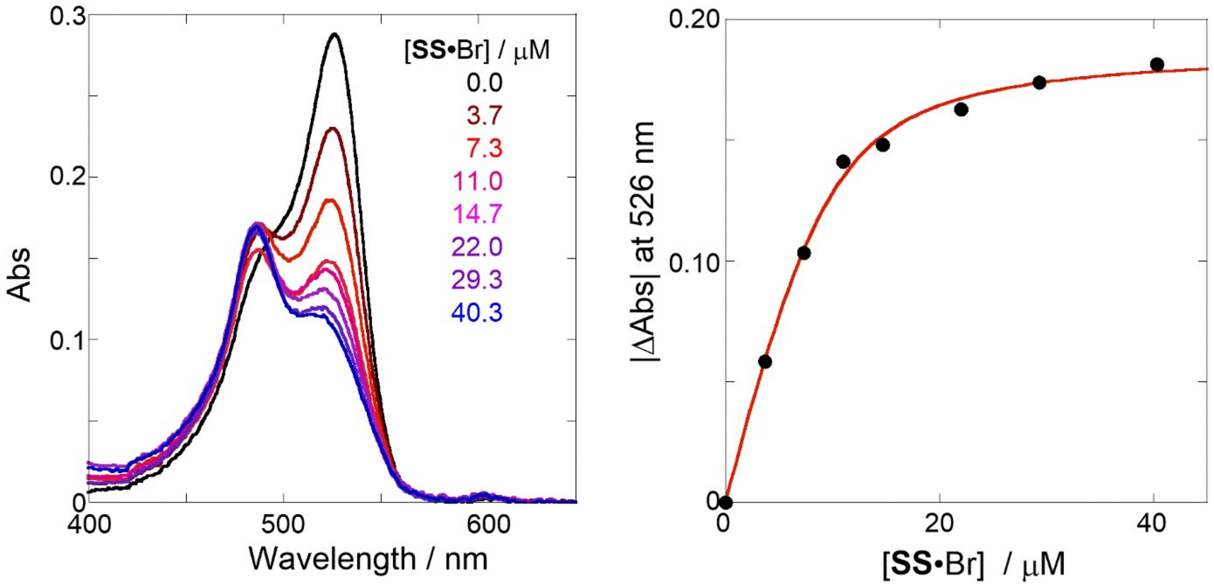


(h)

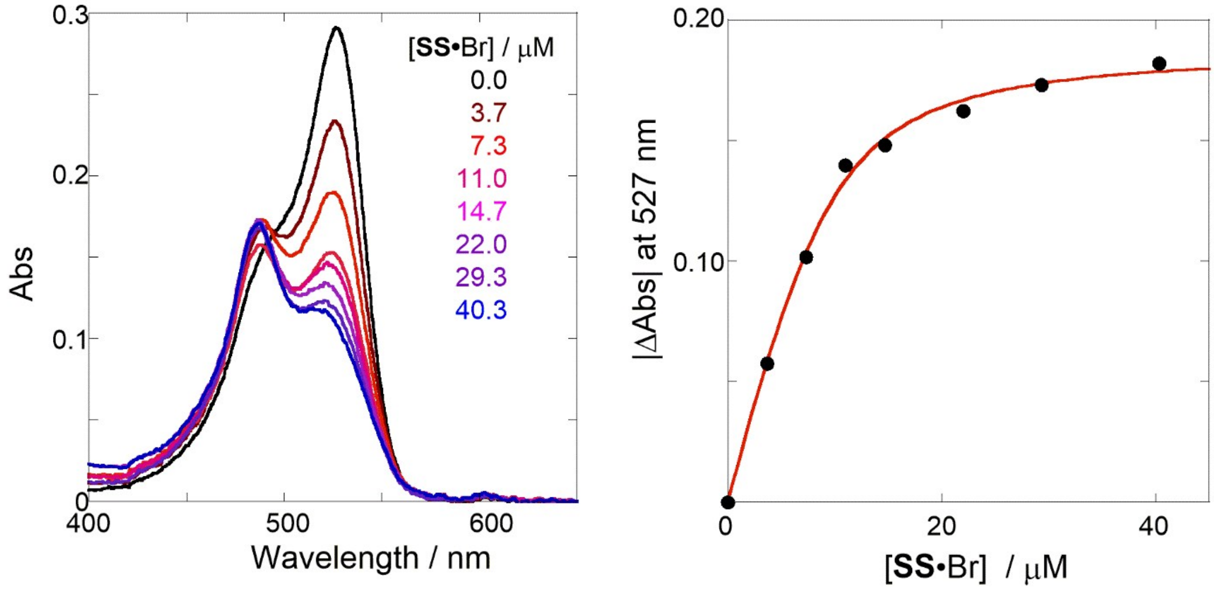


**Figure S15.** (*Left panels*) UV/vis absorption spectral changes of a chloroform solution containing methanol (0.1 vol%) of **H** (9.3 μM, black line) upon gradual addition of **SS**•Br (3.7–40.3 μM, colored line) and (*Right panels*) nonlinear least-squares fitting, assuming 1:1 stoichiometry of **SS**•Br with **H**, to determine the binding constant ($K_{anion}$) at room temperature at (a) 40, (b) 80, (c) 120, (d) 160, (e) 200, (f) 240, (g) 280, and (h) 320 MPa, measured in a high-pressure cell.

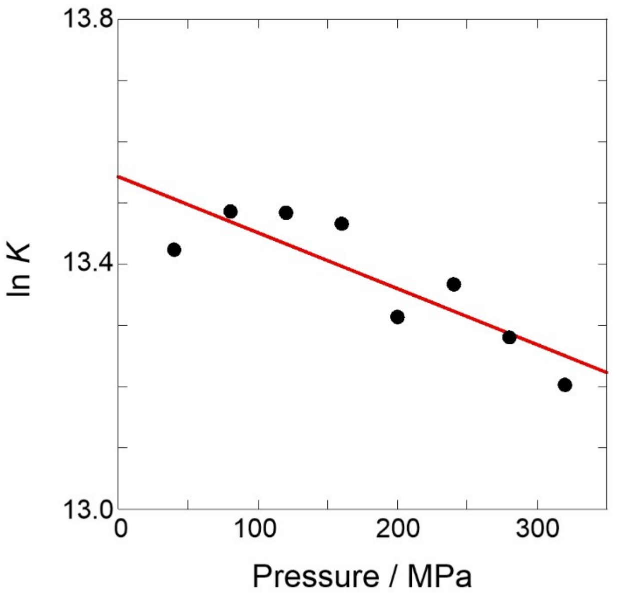


**Figure S16.** Pressure dependence of binding constant ($K_{anion}$) in anion recognition of **H** with **SS**•Br in chloroform containing methanol (0.1 vol%) at room temperature.

(a)

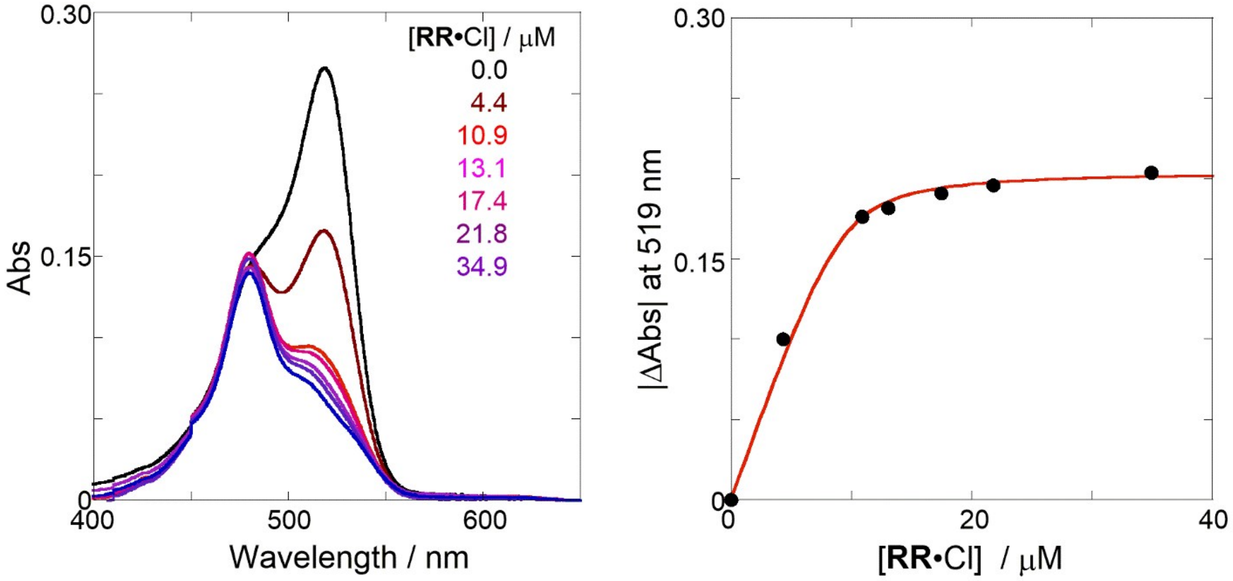
[RR•Cl] / μM
0.0
4.4
10.9
13.1
17.4
21.8
34.9
Abs
Wavelength / nm
|ΔAbs| at 519 nm
[RR•Cl] / μM

(b)

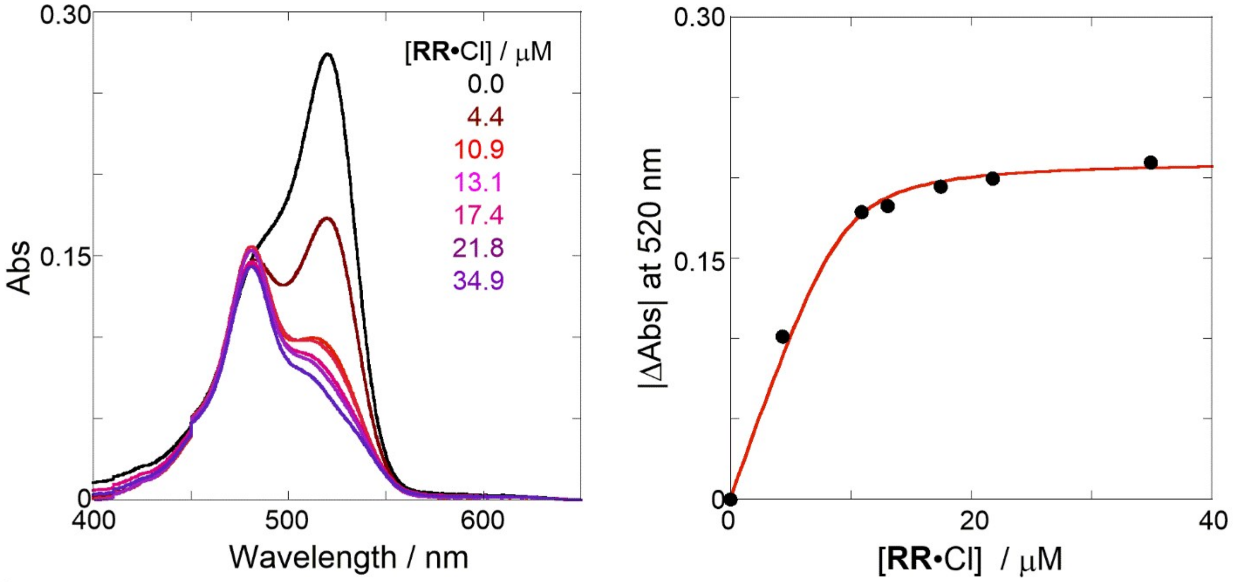
[RR•Cl] / μM
0.0
4.4
10.9
13.1
17.4
21.8
34.9
Abs
Wavelength / nm
|ΔAbs| at 520 nm
[RR•Cl] / μM

(c)

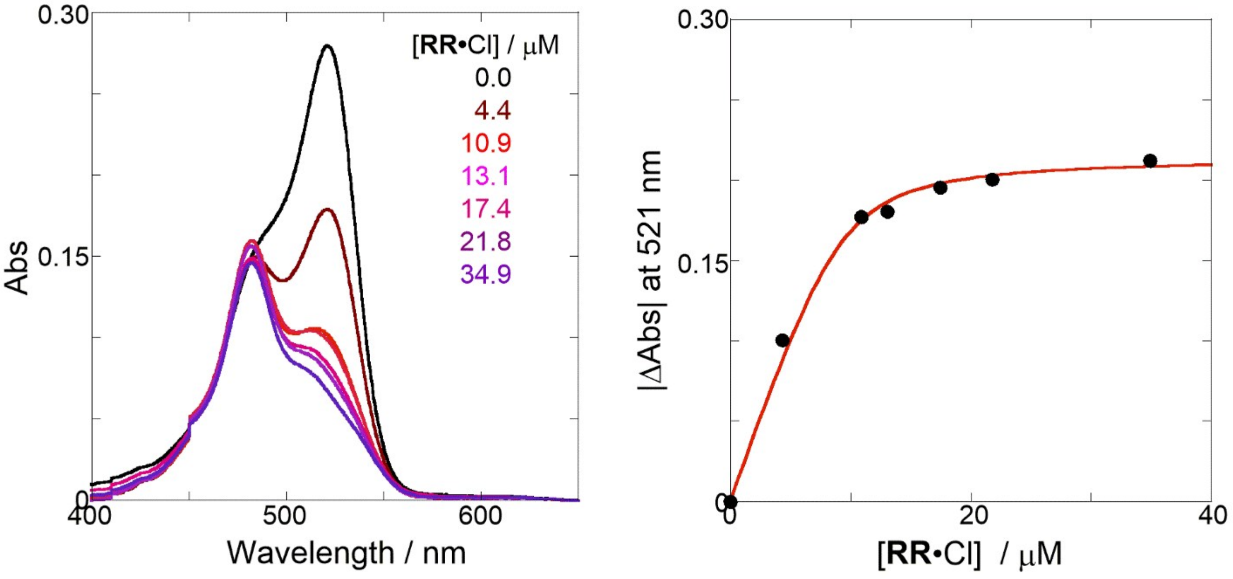
[RR•Cl] / μM
0.0
4.4
10.9
13.1
17.4
21.8
34.9
Abs
Wavelength / nm
|ΔAbs| at 521 nm
[RR•Cl] / μM

(d)

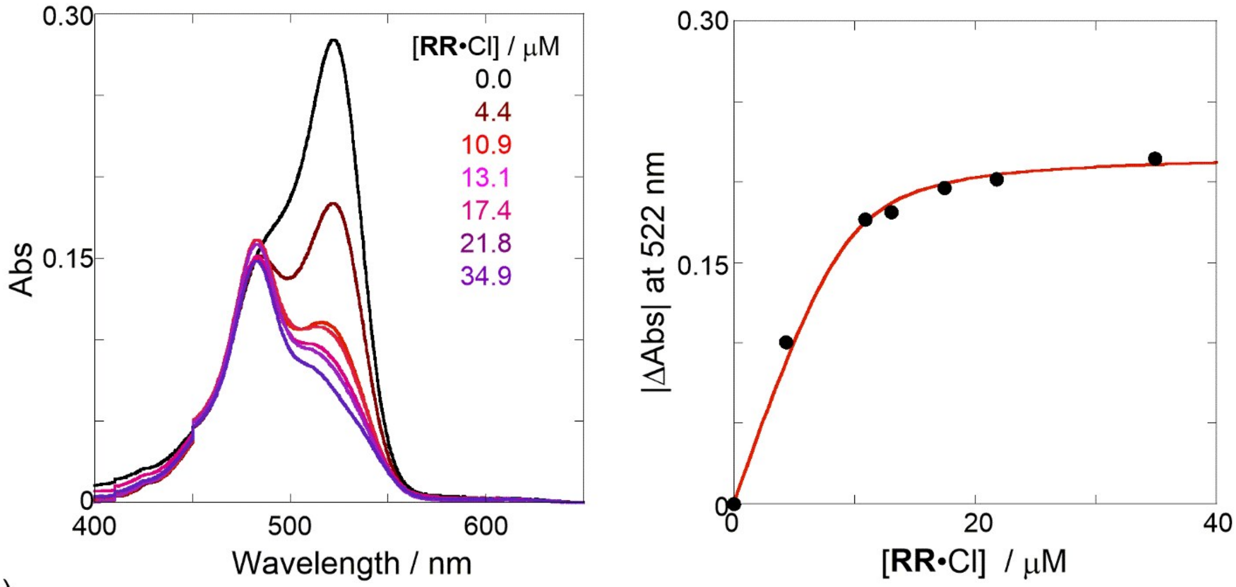

[RR•Cl] / μM
0.0
4.4
10.9
13.1
17.4
21.8
34.9
Abs
Wavelength / nm
|ΔAbs| at 522 nm
[RR•Cl] / μM


(e)

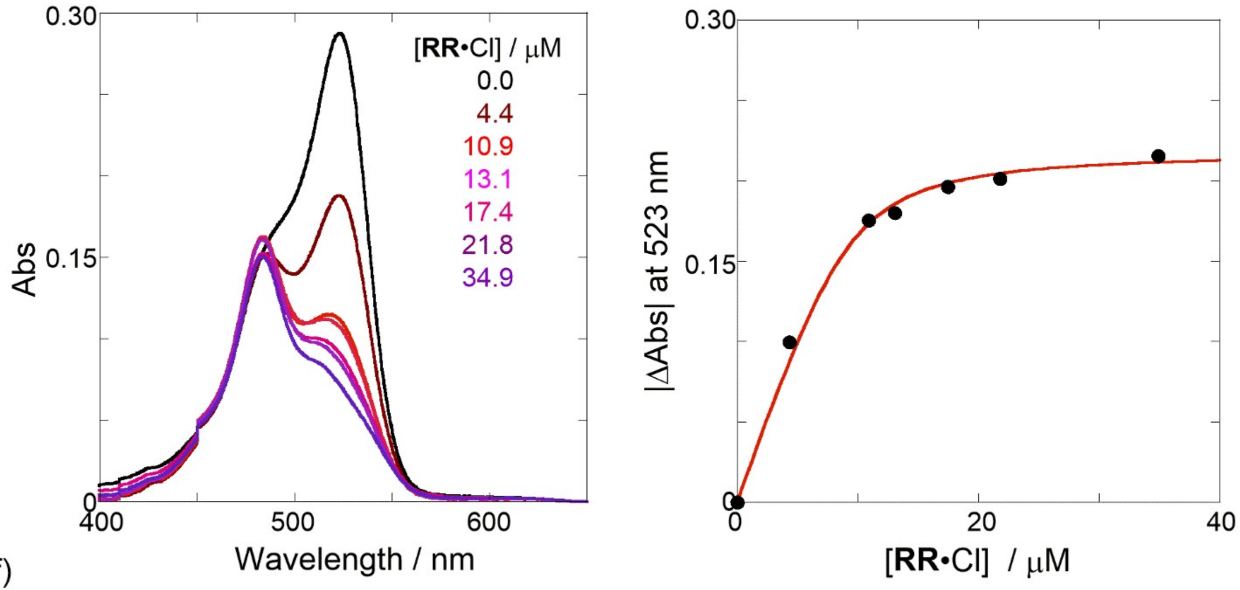

[RR•Cl] / μM
0.0
4.4
10.9
13.1
17.4
21.8
34.9
Abs
Wavelength / nm
|ΔAbs| at 523 nm
[RR•Cl] / μM


(f)

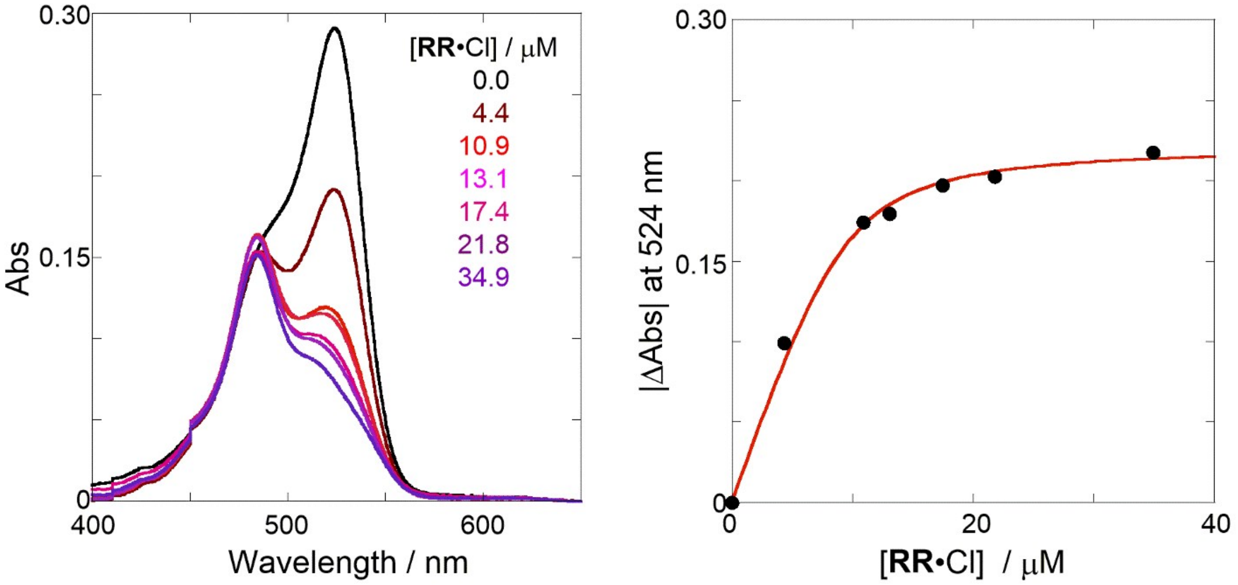

[RR•Cl] / μM
0.0
4.4
10.9
13.1
17.4
21.8
34.9
Abs
Wavelength / nm
|ΔAbs| at 524 nm
[RR•Cl] / μM

(g)

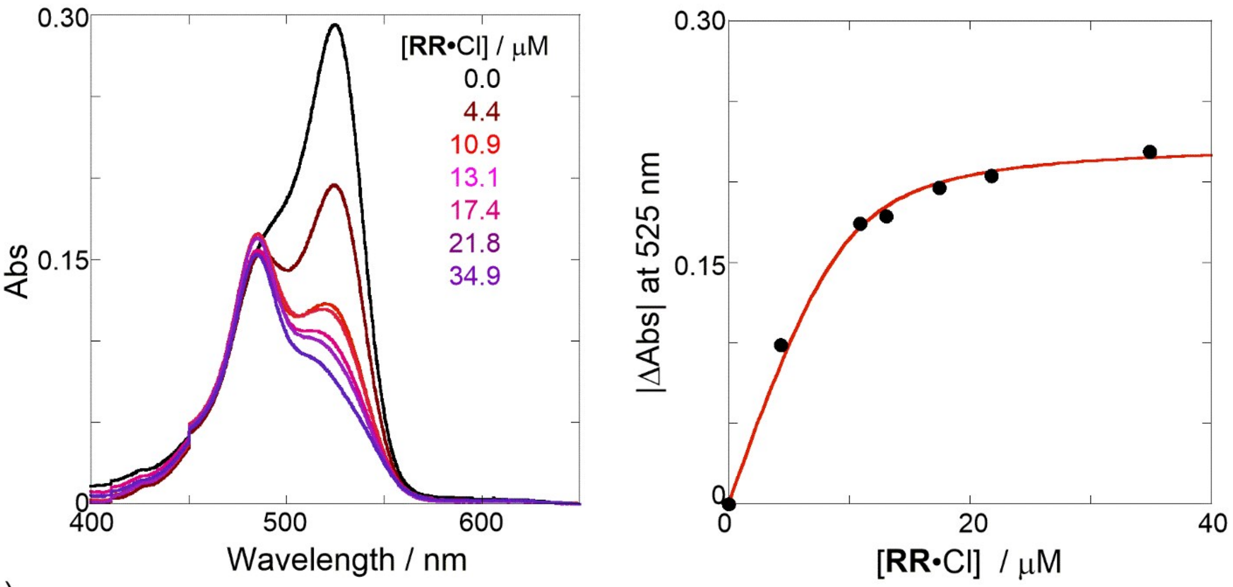


(h)

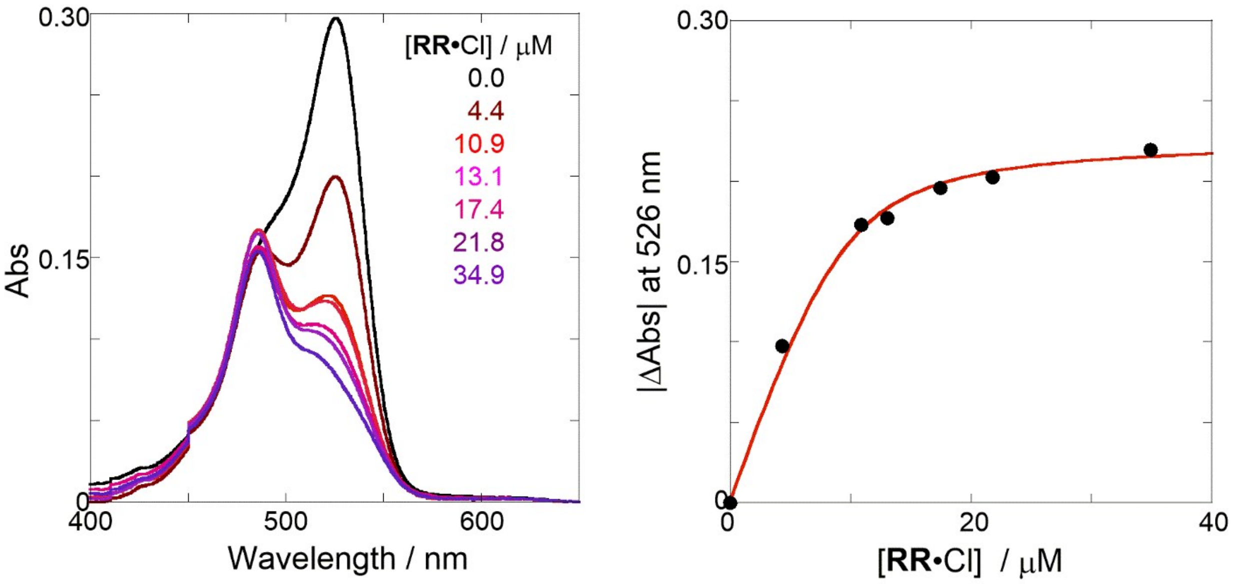


**Figure S17.** (*Left panels*) UV/vis absorption spectral changes of a chloroform solution containing methanol (0.2 vol%) of **H** (9.3 μM, black line) upon gradual addition of **RR**•Cl (4.4–34.9 μM, colored line) and (*Right panels*) nonlinear least-squares fitting, assuming 1:1 stoichiometry of **RR**•Cl with **H**, to determine the binding constant ($K_{anion}$) at room temperature at (a) 40, (b) 80, (c) 120, (d) 160, (e) 200, (f) 240, (g) 280, and (h) 320 MPa, measured in a high-pressure cell.

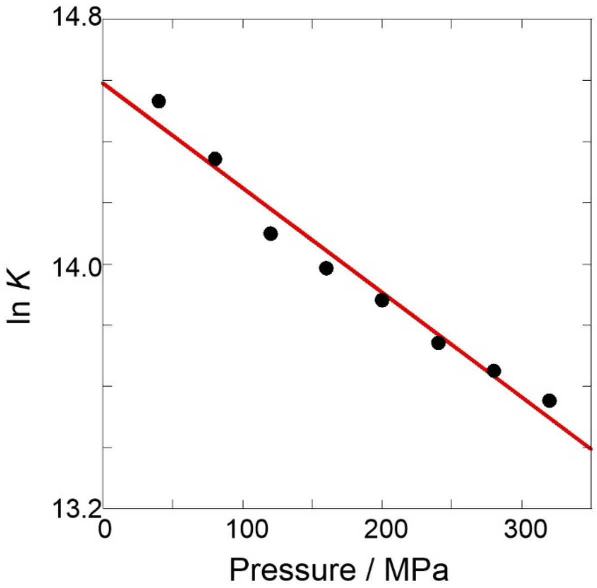


**Figure S18.** Pressure dependence of binding constant ($K_{anion}$) in anion recognition of **H** with **RR**•Cl in chloroform containing methanol (0.2 vol%) at room temperature.

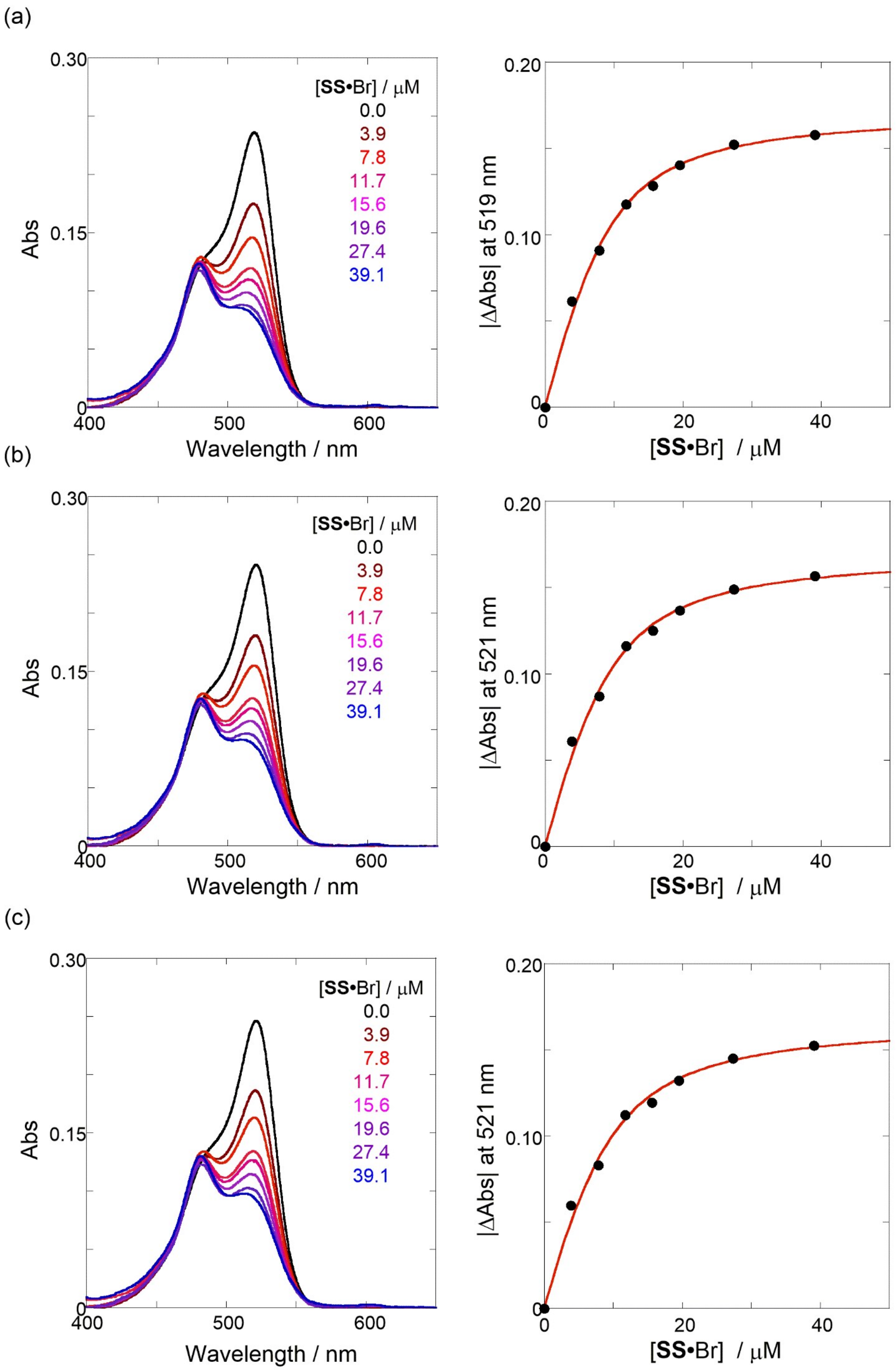
(a)
0.30
0.15
0
Abs
400
500
600
Wavelength / nm
[SS•Br] / μM
0.0
3.9
7.8
11.7
15.6
19.6
27.4
39.1
0.20
0.10
0
|ΔAbs| at 519 nm
0
20
40
[SS•Br] / μM
(b)
0.30
0.15
0
Abs
400
500
600
Wavelength / nm
[SS•Br] / μM
0.0
3.9
7.8
11.7
15.6
19.6
27.4
39.1
0.20
0.10
0
|ΔAbs| at 521 nm
0
20
40
[SS•Br] / μM
(c)
0.30
0.15
0
Abs
400
500
600
Wavelength / nm
[SS•Br] / μM
0.0
3.9
7.8
11.7
15.6
19.6
27.4
39.1
0.20
0.10
0
|ΔAbs| at 521 nm
0
20
40
[SS•Br] / μM

(d)

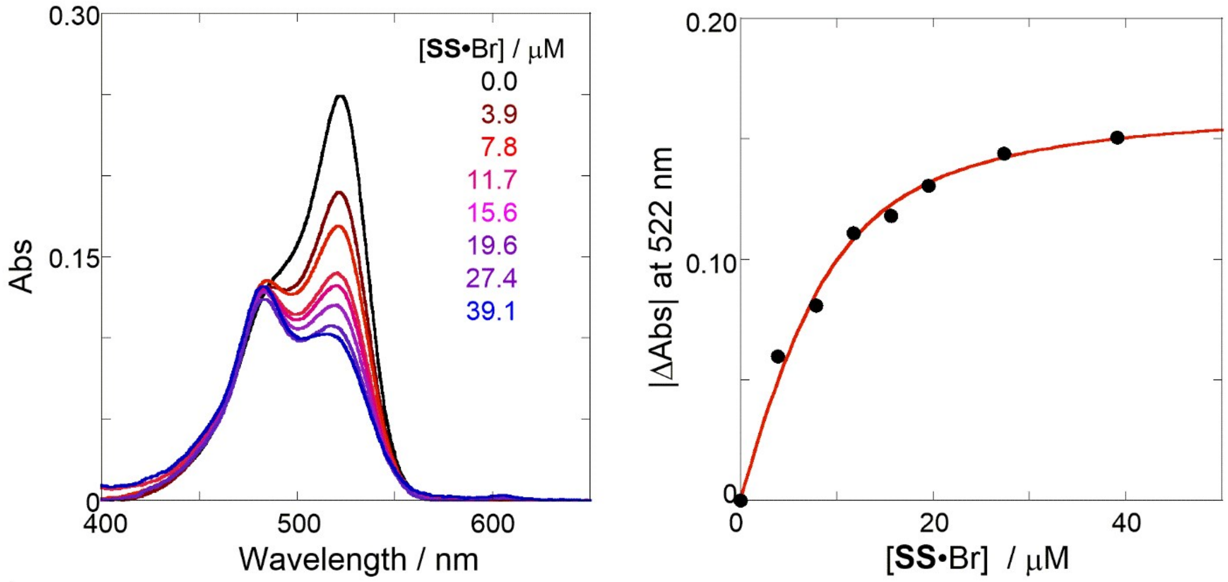
0.30
0.15
0
Abs
400
500
600
Wavelength / nm
[SS•Br] / μM
0.0
3.9
7.8
11.7
15.6
19.6
27.4
39.1
0.20
0.10
0
|ΔAbs| at 522 nm
0
20
40
[SS•Br] / μM

(e)

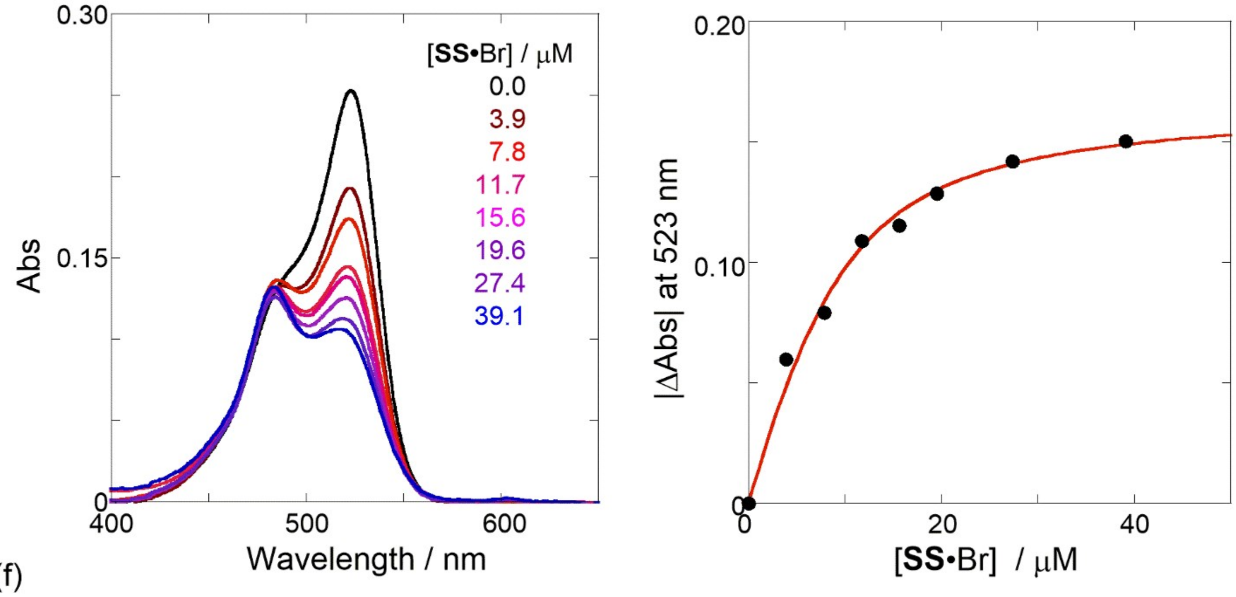
0.30
0.15
0
Abs
400
500
600
Wavelength / nm
[SS•Br] / μM
0.0
3.9
7.8
11.7
15.6
19.6
27.4
39.1
0.20
0.10
0
|ΔAbs| at 523 nm
0
20
40
[SS•Br] / μM

(f)

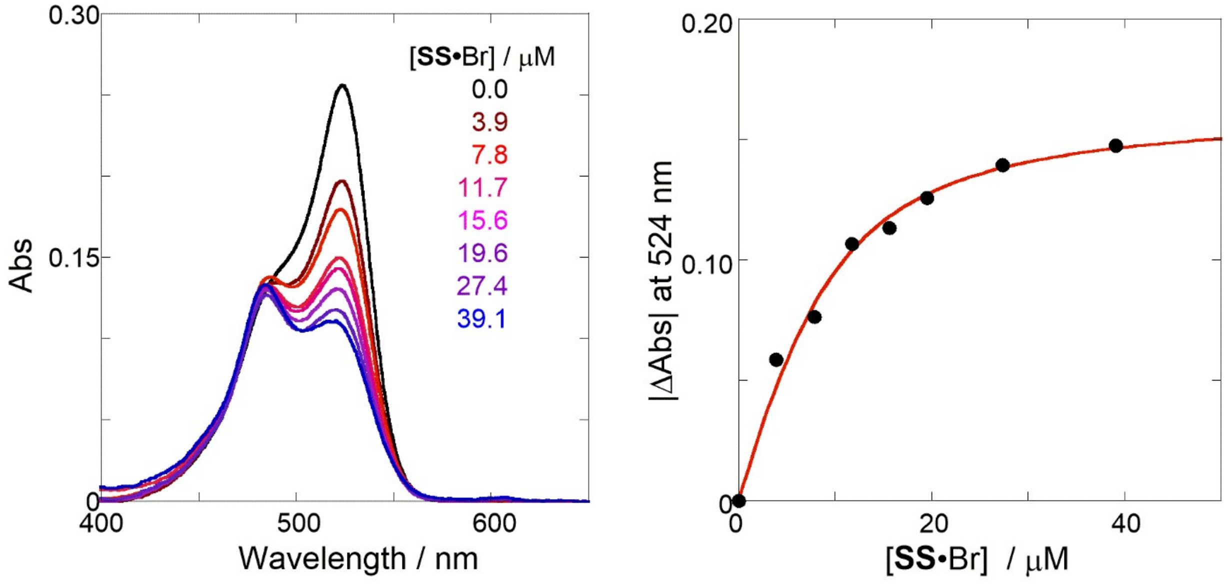
0.30
0.15
0
Abs
400
500
600
Wavelength / nm
[SS•Br] / μM
0.0
3.9
7.8
11.7
15.6
19.6
27.4
39.1
0.20
0.10
0
|ΔAbs| at 524 nm
0
20
40
[SS•Br] / μM

(g)

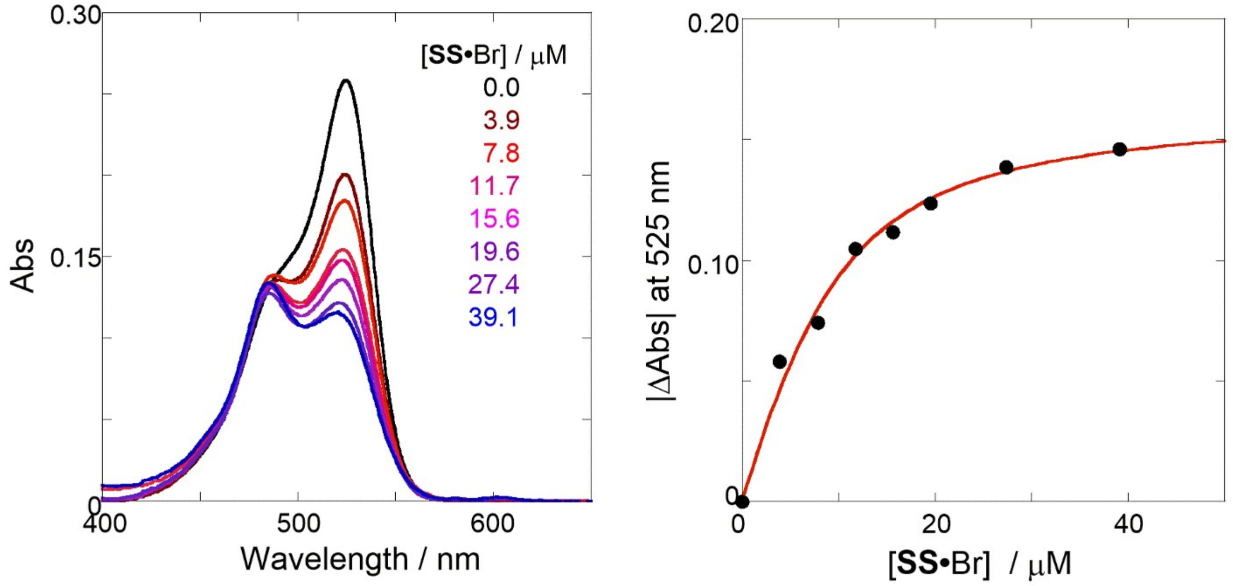


(h)

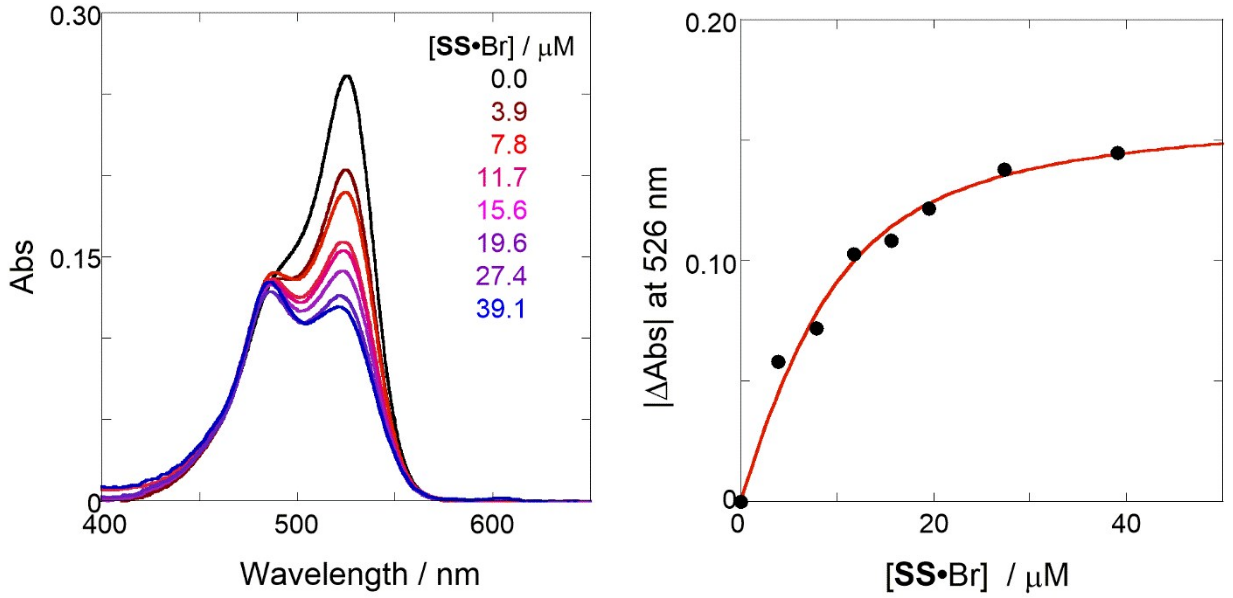


**Figure S19.** (*Left panels*) UV/vis absorption spectral changes of a chloroform solution containing methanol (0.2 vol%) of **H** (8.3 μM, black line) upon gradual addition of **SS**•Br (3.9–39.1 μM, colored line) and (*Right panels*) nonlinear least-squares fitting, assuming 1:1 stoichiometry of **SS**•Br with **H**, to determine the binding constant ($K_{anion}$) at room temperature at (a) 40, (b) 80, (c) 120, (d) 160, (e) 200, (f) 240, (g) 280, and (h) 320 MPa, measured in a high-pressure cell.

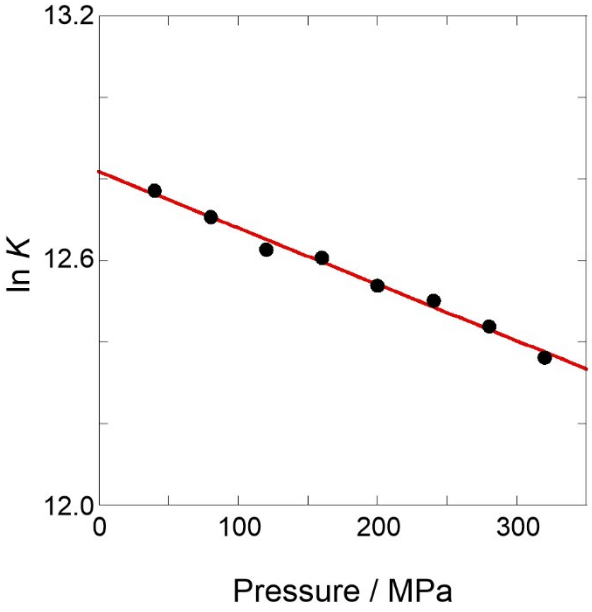


**Figure S20.** Pressure dependence of binding constant ($K_{anion}$) in anion recognition of **H** with **SS**•Br in chloroform containing methanol (0.2 vol%) at room temperature.

(a)

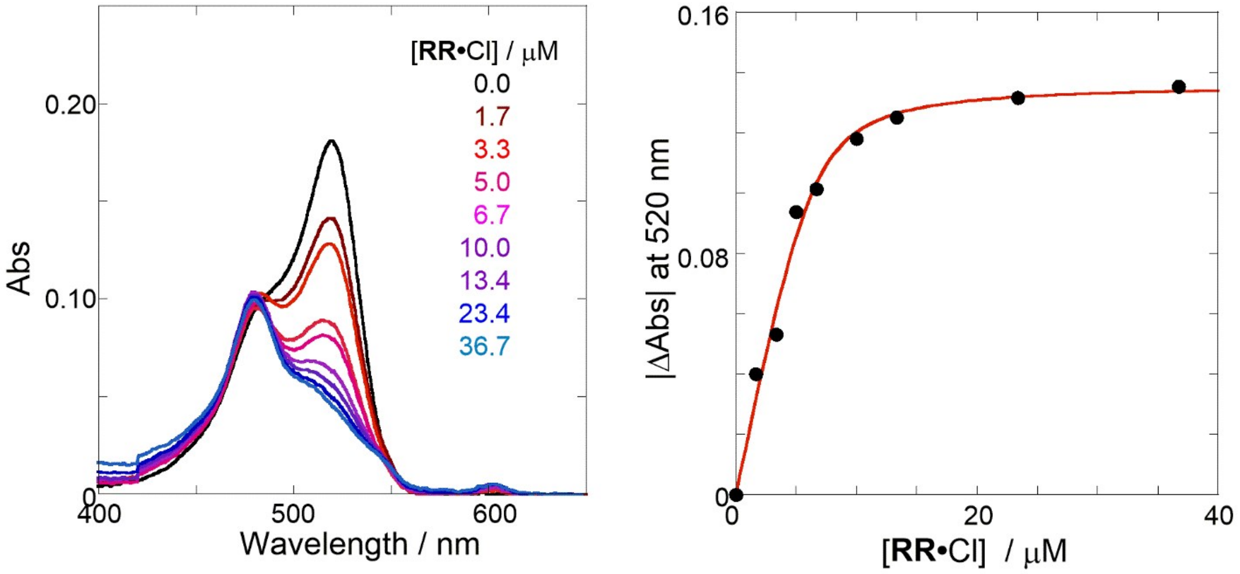
[RR•Cl] / μM
0.0
1.7
3.3
5.0
6.7
10.0
13.4
23.4
36.7
Abs
Wavelength / nm
|ΔAbs| at 520 nm
[RR•Cl] / μM

(b)

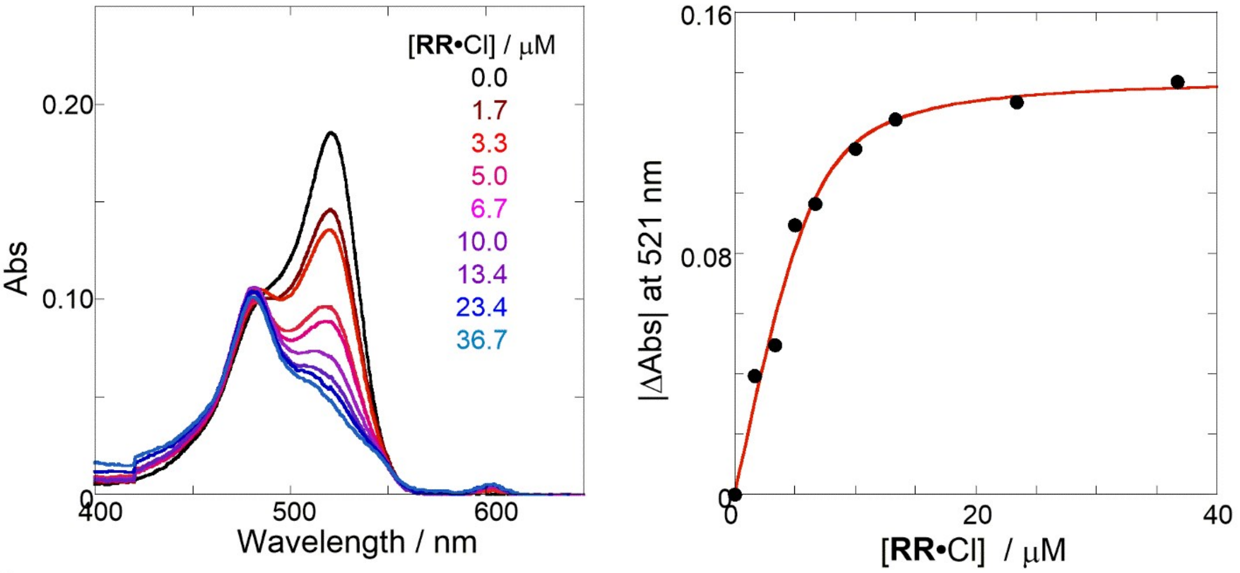
[RR•Cl] / μM
0.0
1.7
3.3
5.0
6.7
10.0
13.4
23.4
36.7
Abs
Wavelength / nm
|ΔAbs| at 521 nm
[RR•Cl] / μM

(c)

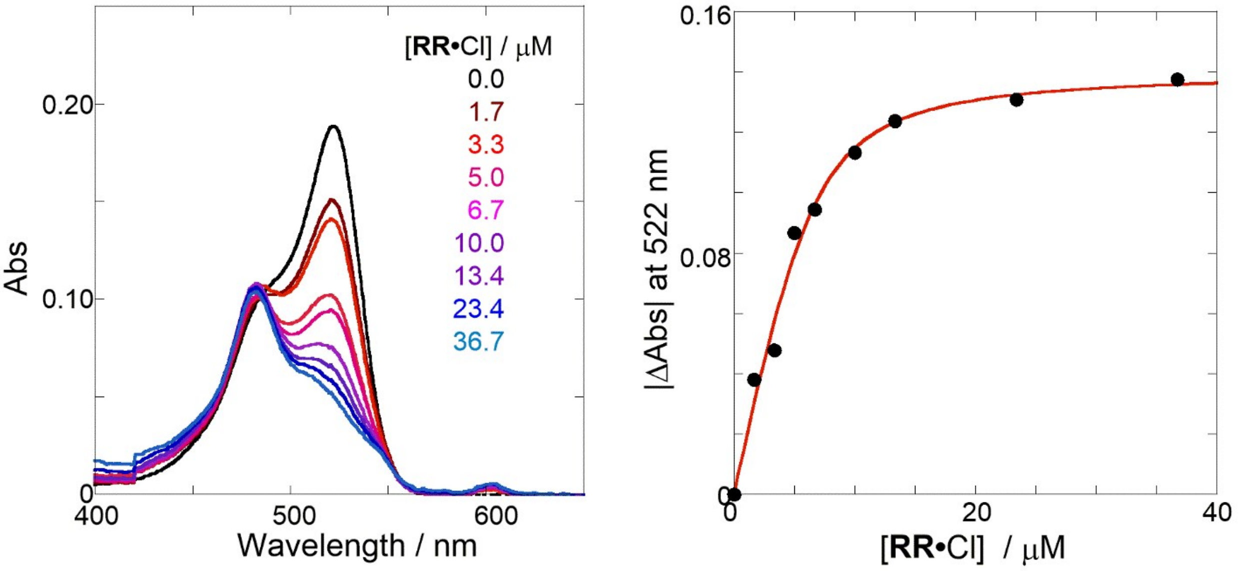
[RR•Cl] / μM
0.0
1.7
3.3
5.0
6.7
10.0
13.4
23.4
36.7
Abs
Wavelength / nm
|ΔAbs| at 522 nm
[RR•Cl] / μM

(d)

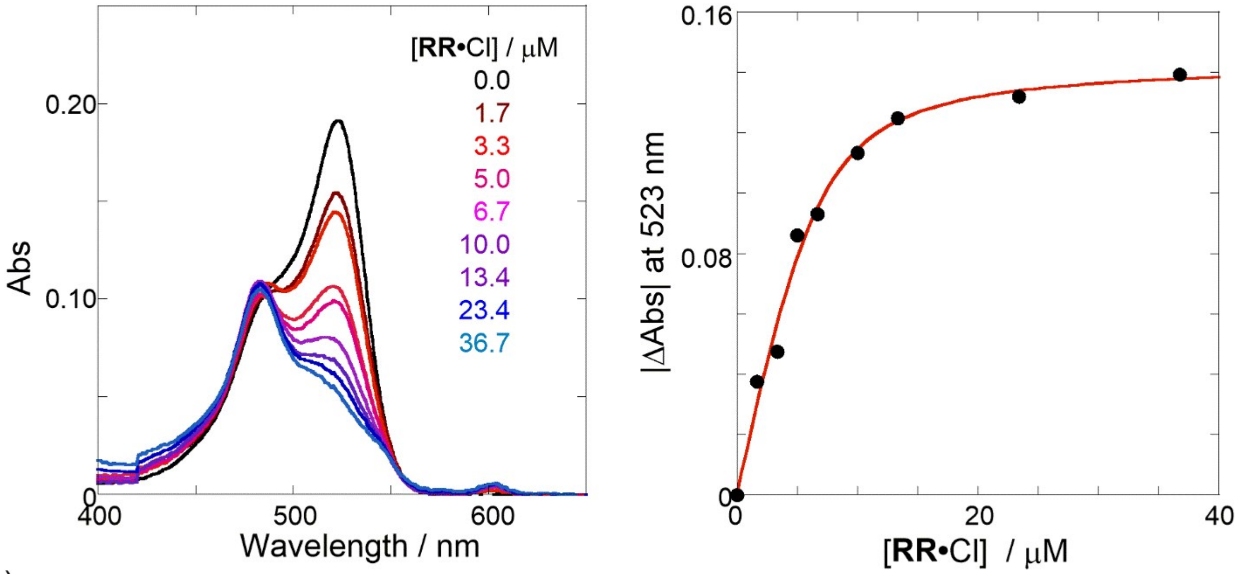
[RR•Cl] / μM
0.0
1.7
3.3
5.0
6.7
10.0
13.4
23.4
36.7
Abs
0.20
0.10
0
400
500
600
Wavelength / nm
|ΔAbs| at 523 nm
0.16
0.08
0
20
40
[RR•Cl] / μM

(e)

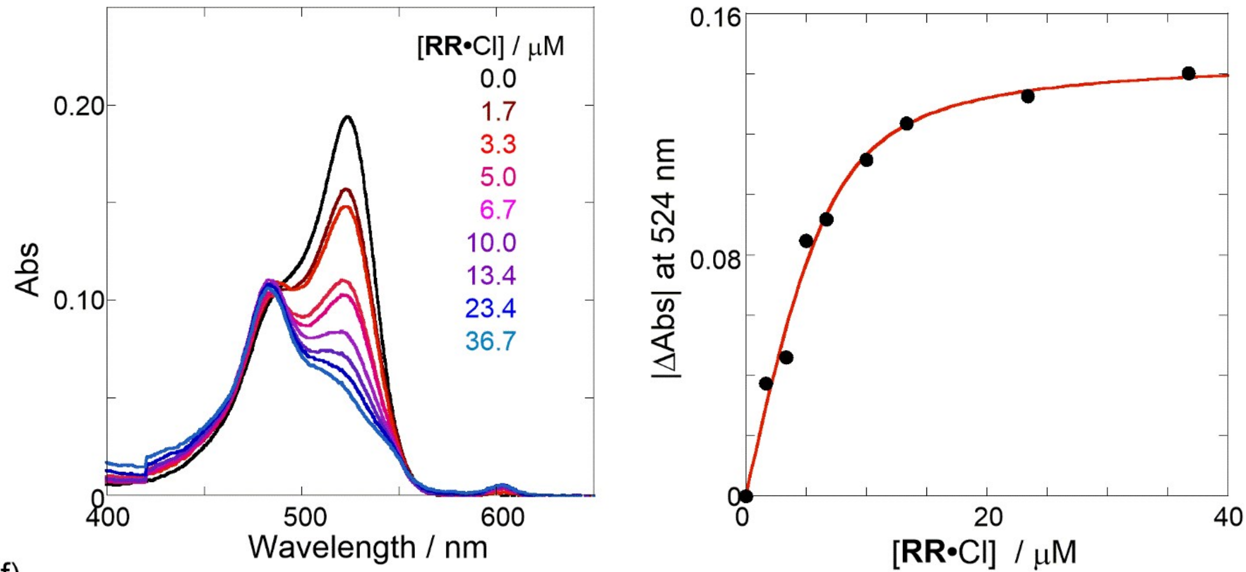
[RR•Cl] / μM
0.0
1.7
3.3
5.0
6.7
10.0
13.4
23.4
36.7
Abs
0.20
0.10
0
400
500
600
Wavelength / nm
|ΔAbs| at 524 nm
0.16
0.08
0
20
40
[RR•Cl] / μM

(f)

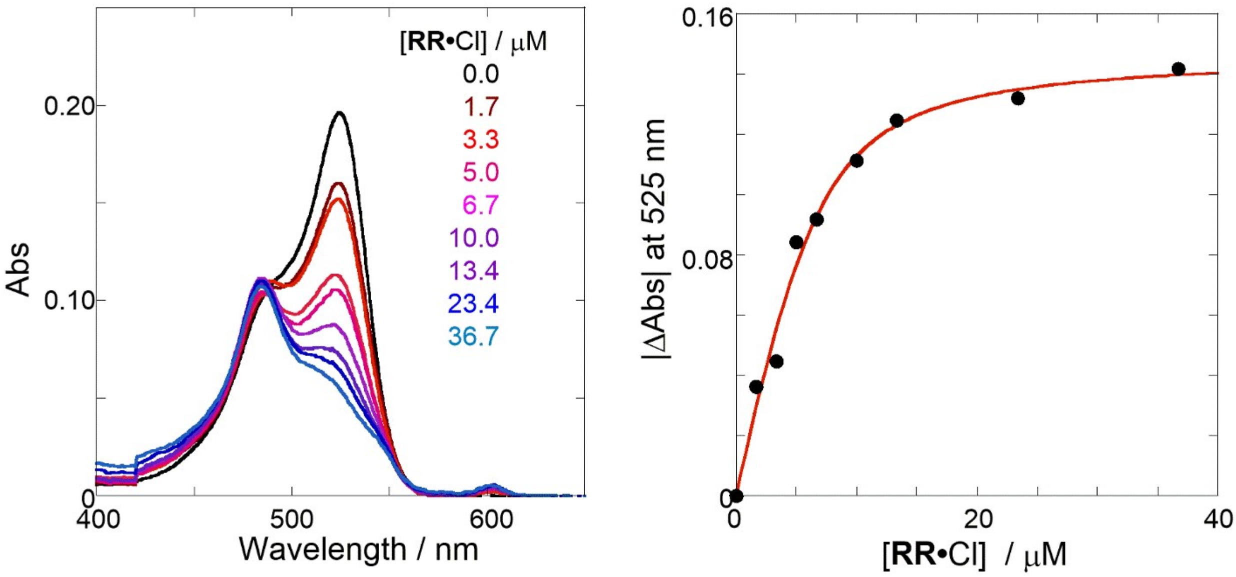
[RR•Cl] / μM
0.0
1.7
3.3
5.0
6.7
10.0
13.4
23.4
36.7
Abs
0.20
0.10
0
400
500
600
Wavelength / nm
|ΔAbs| at 525 nm
0.16
0.08
0
20
40
[RR•Cl] / μM

(g)

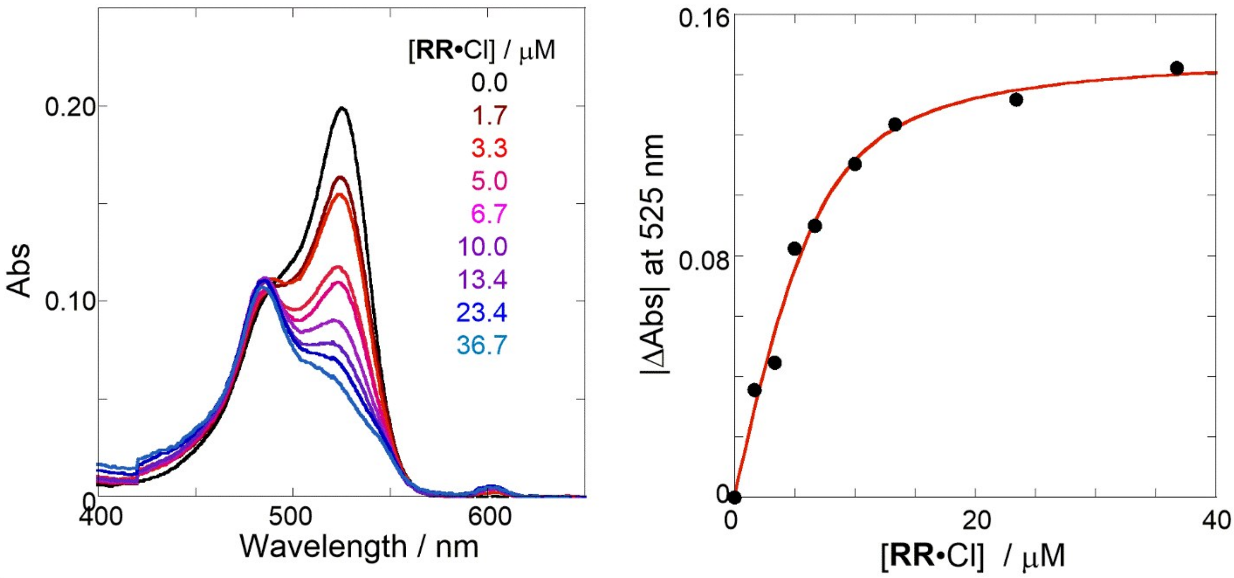


(h)

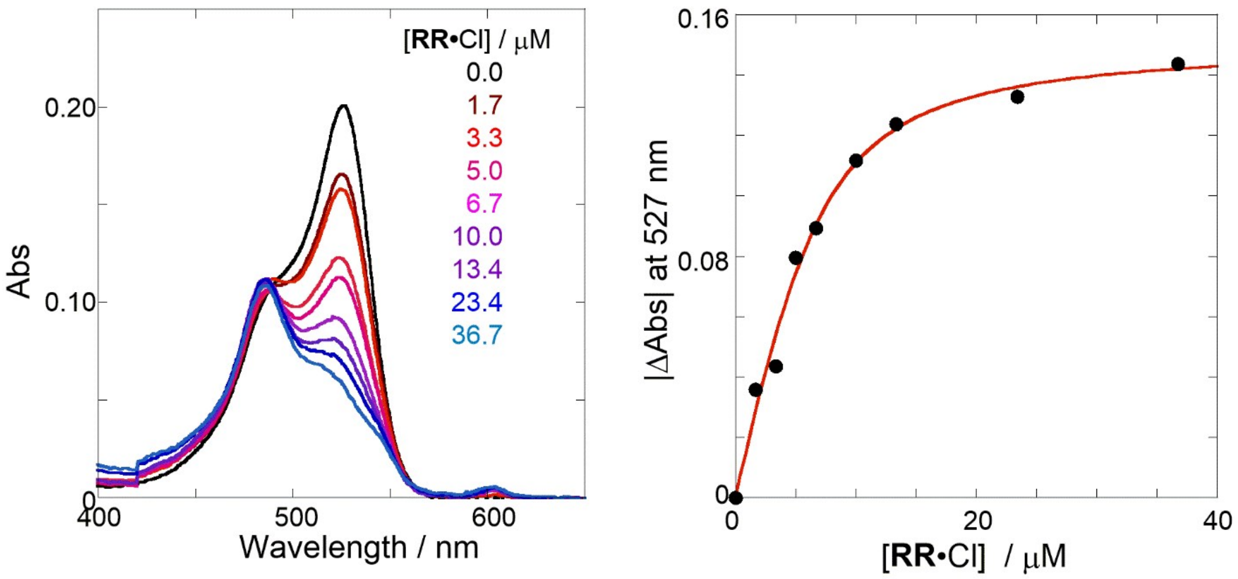


**Figure S21.** (*Left panels*) UV/vis absorption spectral changes of a chloroform solution containing methanol (0.3 vol%) of **H** (6.4 μM, black line) upon gradual addition of **RR**•Cl (1.7–36.7 μM, colored line) and (*Right panels*) nonlinear least-squares fitting, assuming 1:1 stoichiometry of **RR**•Cl with **H**, to determine the binding constant ($K_{anion}$) at room temperature at (a) 40, (b) 80, (c) 120, (d) 160, (e) 200, (f) 240, (g) 280, and (h) 320 MPa, measured in a high-pressure cell.

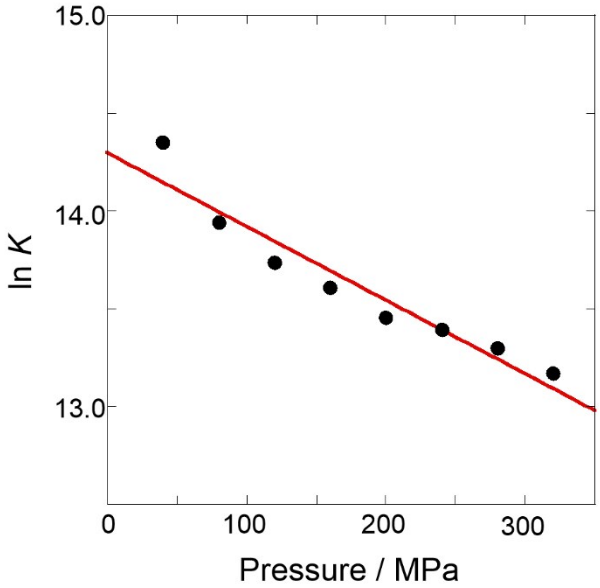


**Figure S22.** Pressure dependence of binding constant ($K_{anion}$) in anion recognition of **H** with **RR**•Cl in chloroform containing (0.3 vol%) at room temperature.

(a)

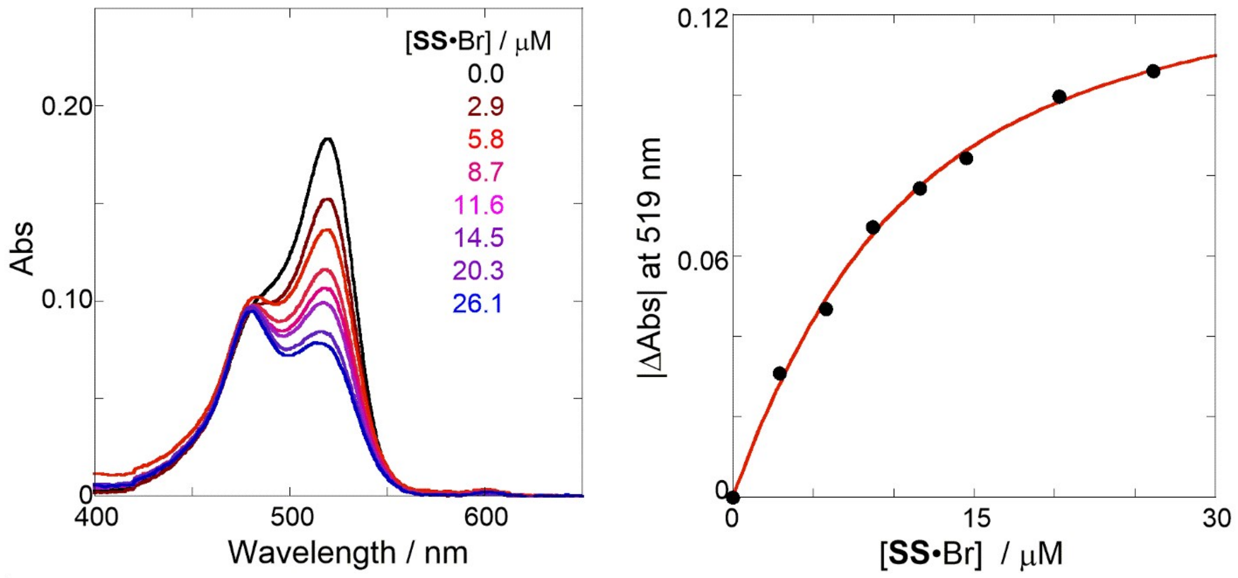
[SS•Br] / μM
0.0
2.9
5.8
8.7
11.6
14.5
20.3
26.1
Abs
0.20
0.10
0
400
500
600
Wavelength / nm
0.12
0.06
0
15
30
|ΔAbs| at 519 nm
[SS•Br] / μM

(b)

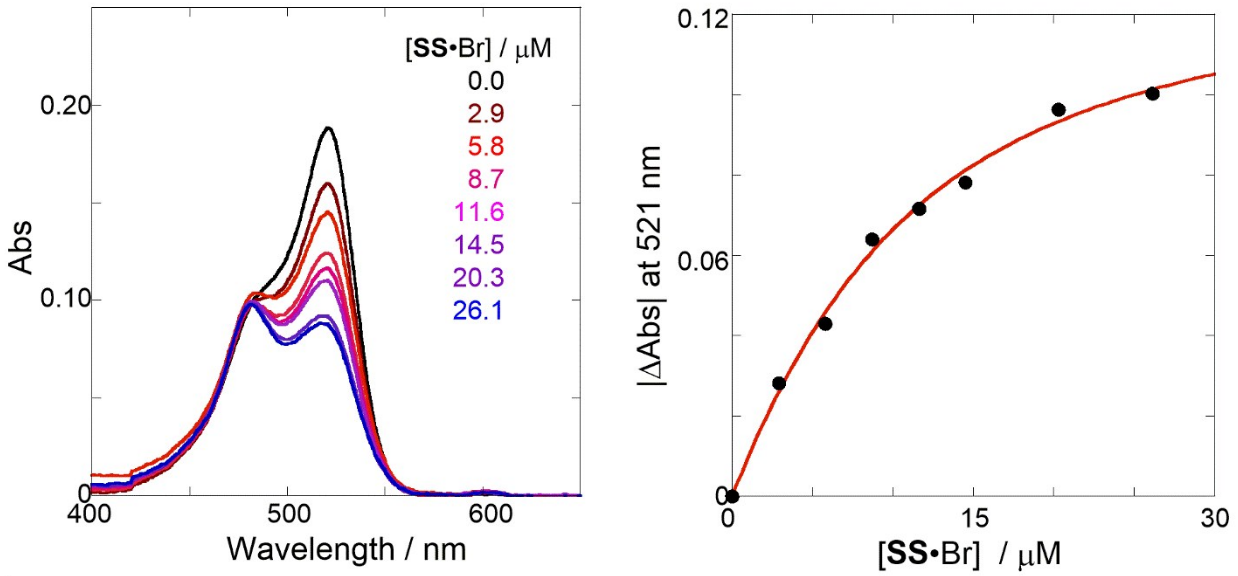
[SS•Br] / μM
0.0
2.9
5.8
8.7
11.6
14.5
20.3
26.1
Abs
0.20
0.10
0
400
500
600
Wavelength / nm
0.12
0.06
0
15
30
|ΔAbs| at 521 nm
[SS•Br] / μM

(c)

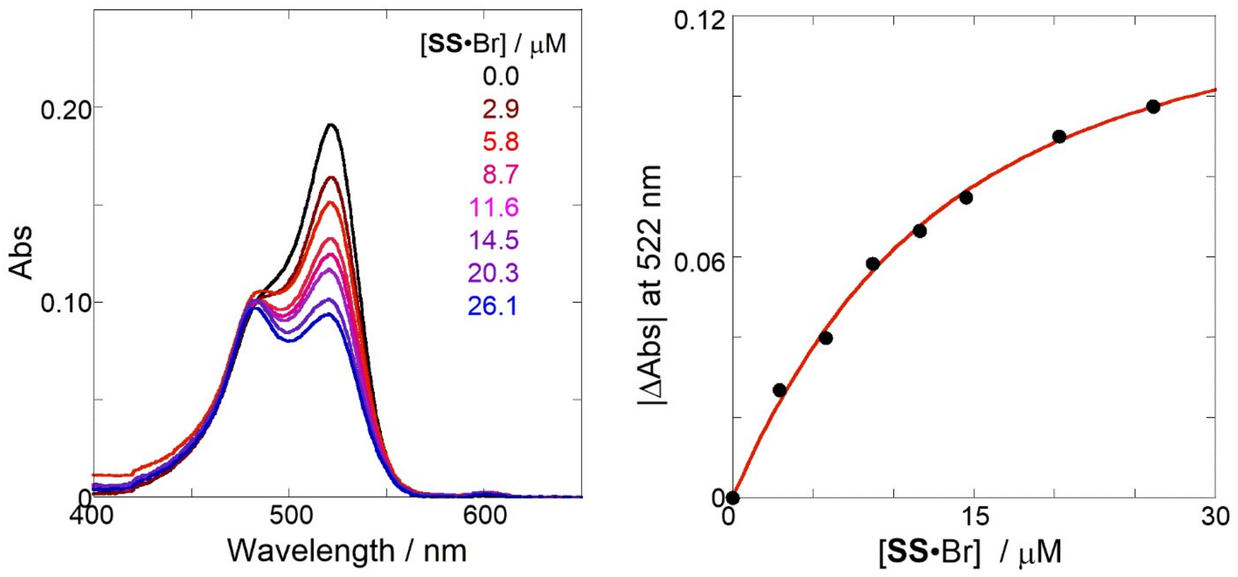
[SS•Br] / μM
0.0
2.9
5.8
8.7
11.6
14.5
20.3
26.1
Abs
0.20
0.10
0
400
500
600
Wavelength / nm
0.12
0.06
0
15
30
|ΔAbs| at 522 nm
[SS•Br] / μM

(d)

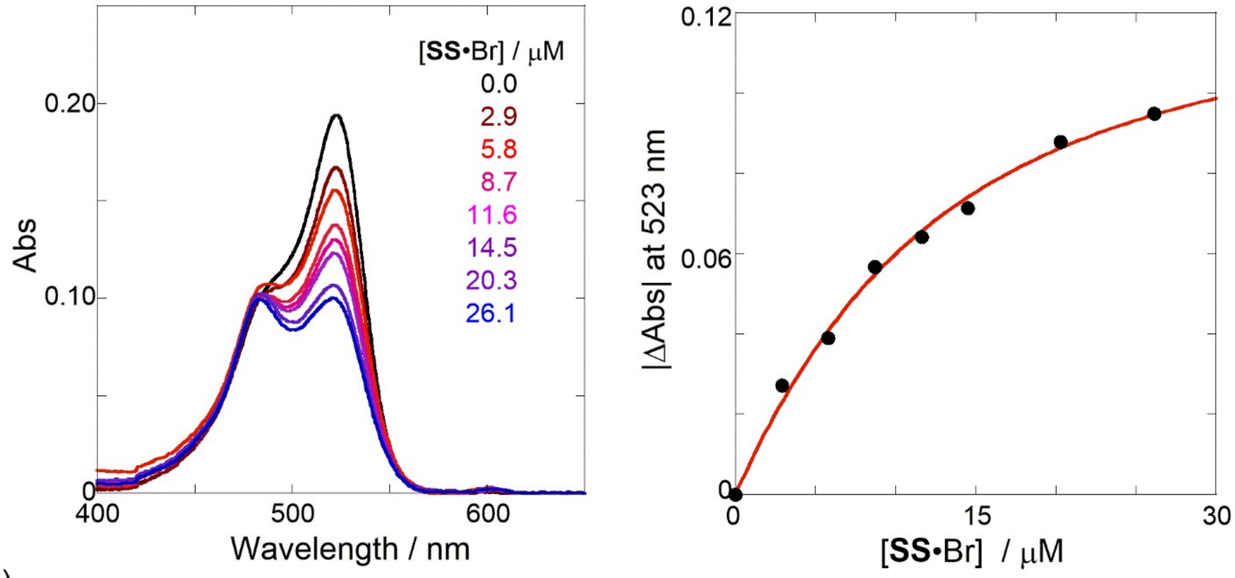
[SS•Br] / μM
0.0
2.9
5.8
8.7
11.6
14.5
20.3
26.1
Abs
0.20
0.10
0
400
500
600
Wavelength / nm
|ΔAbs| at 523 nm
0.12
0.06
0
15
30
[SS•Br] / μM

(e)

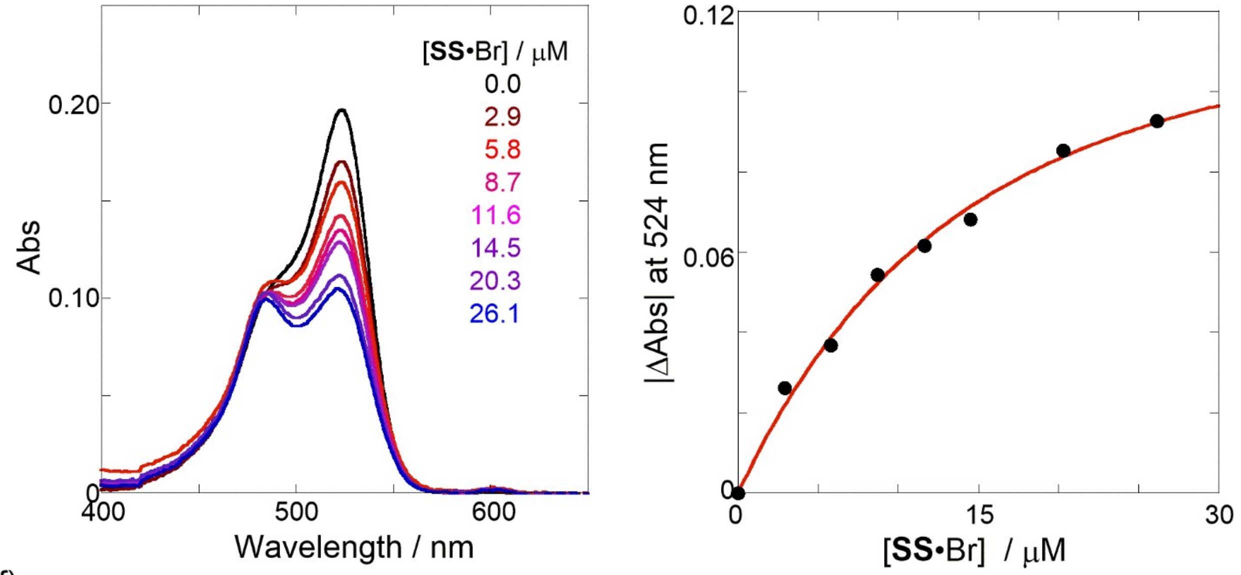
[SS•Br] / μM
0.0
2.9
5.8
8.7
11.6
14.5
20.3
26.1
Abs
0.20
0.10
0
400
500
600
Wavelength / nm
|ΔAbs| at 524 nm
0.12
0.06
0
15
30
[SS•Br] / μM

(f)

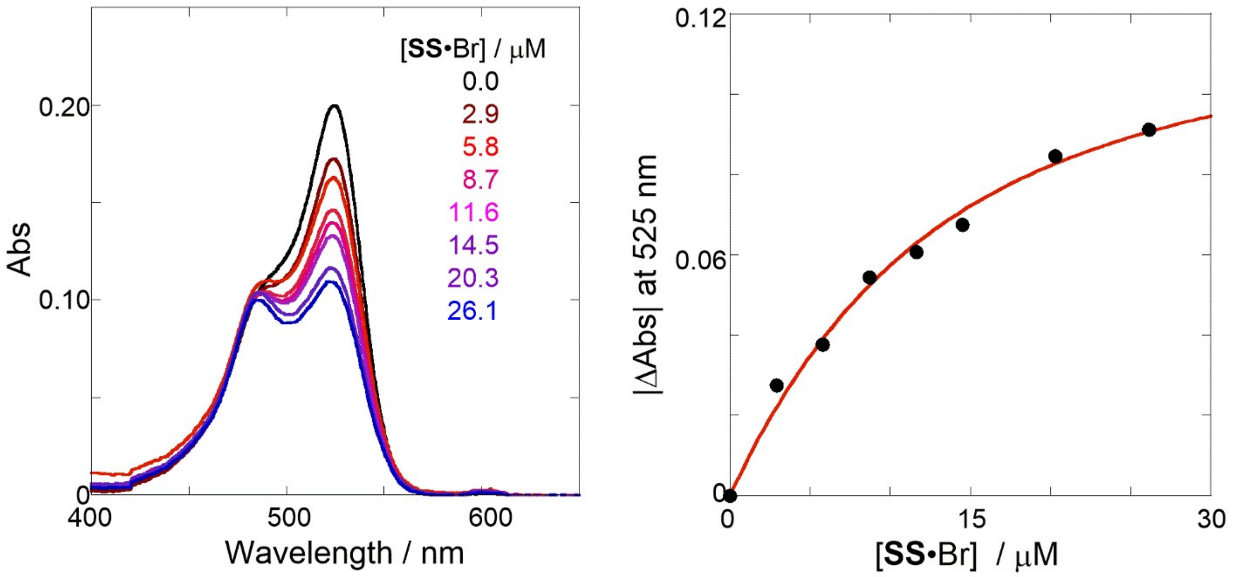
[SS•Br] / μM
0.0
2.9
5.8
8.7
11.6
14.5
20.3
26.1
Abs
0.20
0.10
0
400
500
600
Wavelength / nm
|ΔAbs| at 525 nm
0.12
0.06
0
15
30
[SS•Br] / μM

(g)

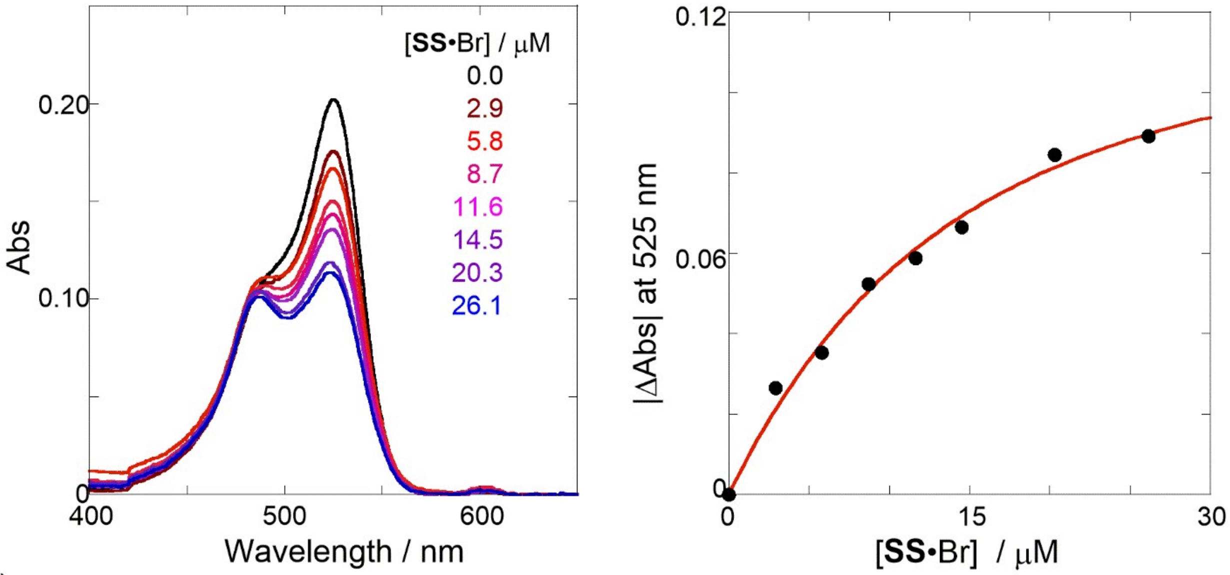


(h)

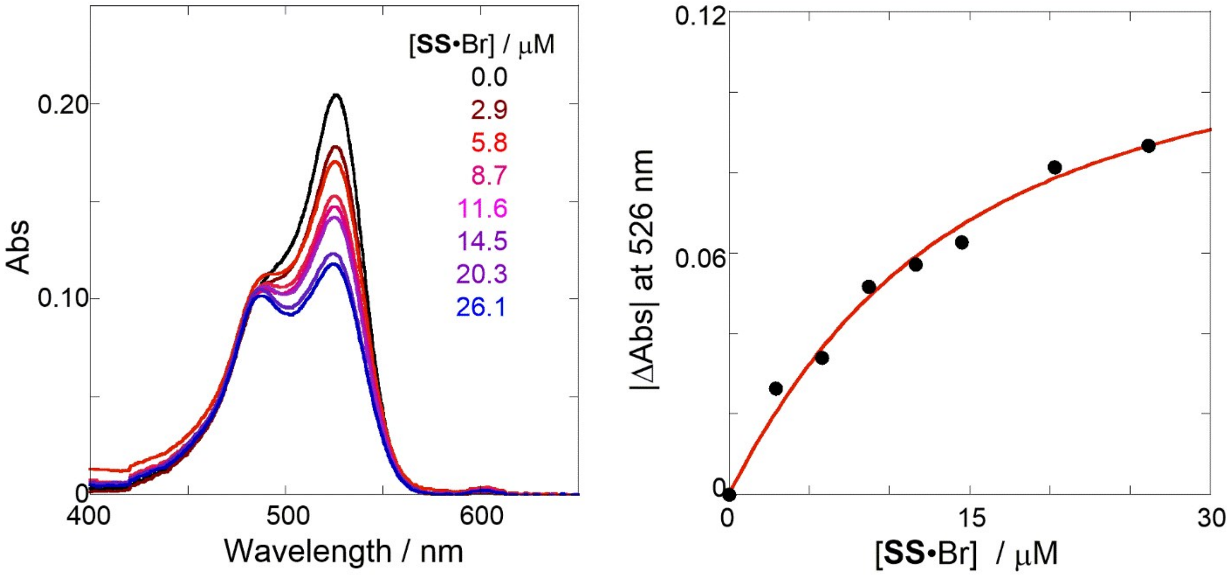


**Figure S23.** (*Left panels*) UV/vis absorption spectral changes of a chloroform solution containing methanol (0.3 vol%) of **H** (6.5 μM, black line) upon gradual addition of **SS**•Br (2.9–26.1 μM, colored line) and (*Right panels*) nonlinear least-squares fitting, assuming 1:1 stoichiometry of **SS**•Br with **H**, to determine the binding constant ($K_{anion}$) at room temperature at (a) 40, (b) 80, (c) 120, (d) 160, (e) 200, (f) 240, (g) 280, and (h) 320 MPa, measured in a high-pressure cell.

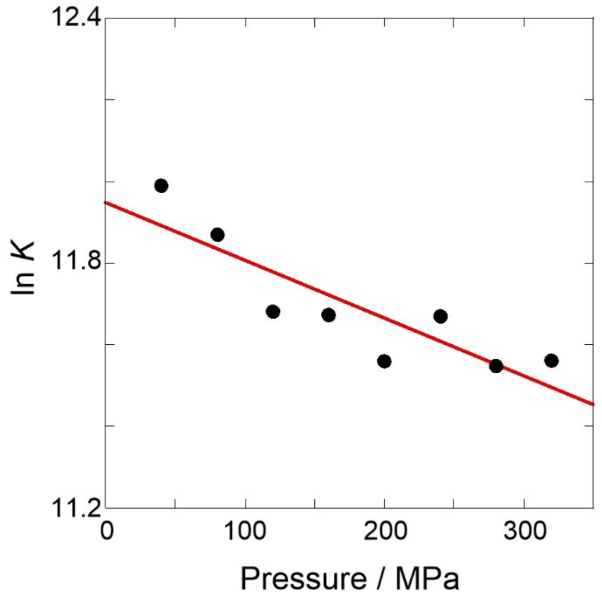


**Figure S24.** Pressure dependence of binding constant ($K_{anion}$) in anion recognition of **H** with **SS**•Br in chloroform containing methanol (0.3 vol%) at room temperature.

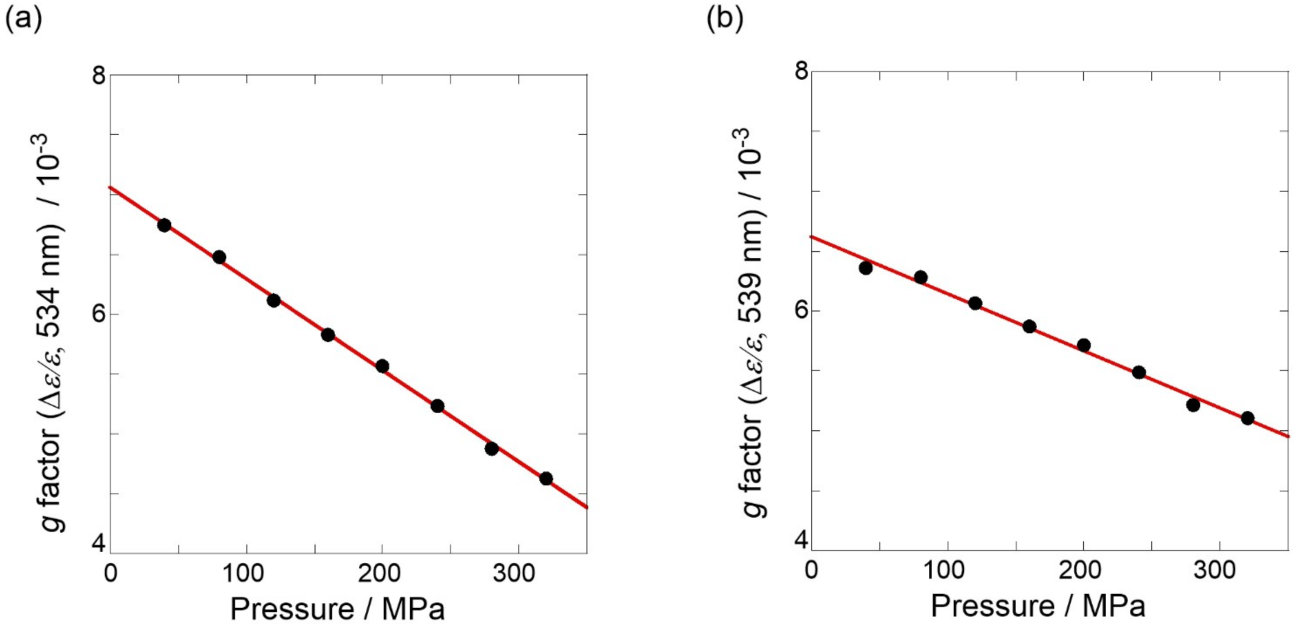


**Figure S25.** Plots of anisotropy (*g* factor) changes for the pressure-induced maxima of chloroform solutions of (a) **H** (34 μM) and **SS**•Cl (120 μM) and (b) **H** (34 μM) and **SS**•Br (130 μM) at room temperature; (a) $r = 0.999$, slope; $-7.66 \times 10^{-6}$ MPa$^{-1}$, (b) $r = 0.995$, slope; $-4.77 \times 10^{-6}$ MPa$^{-1}$.

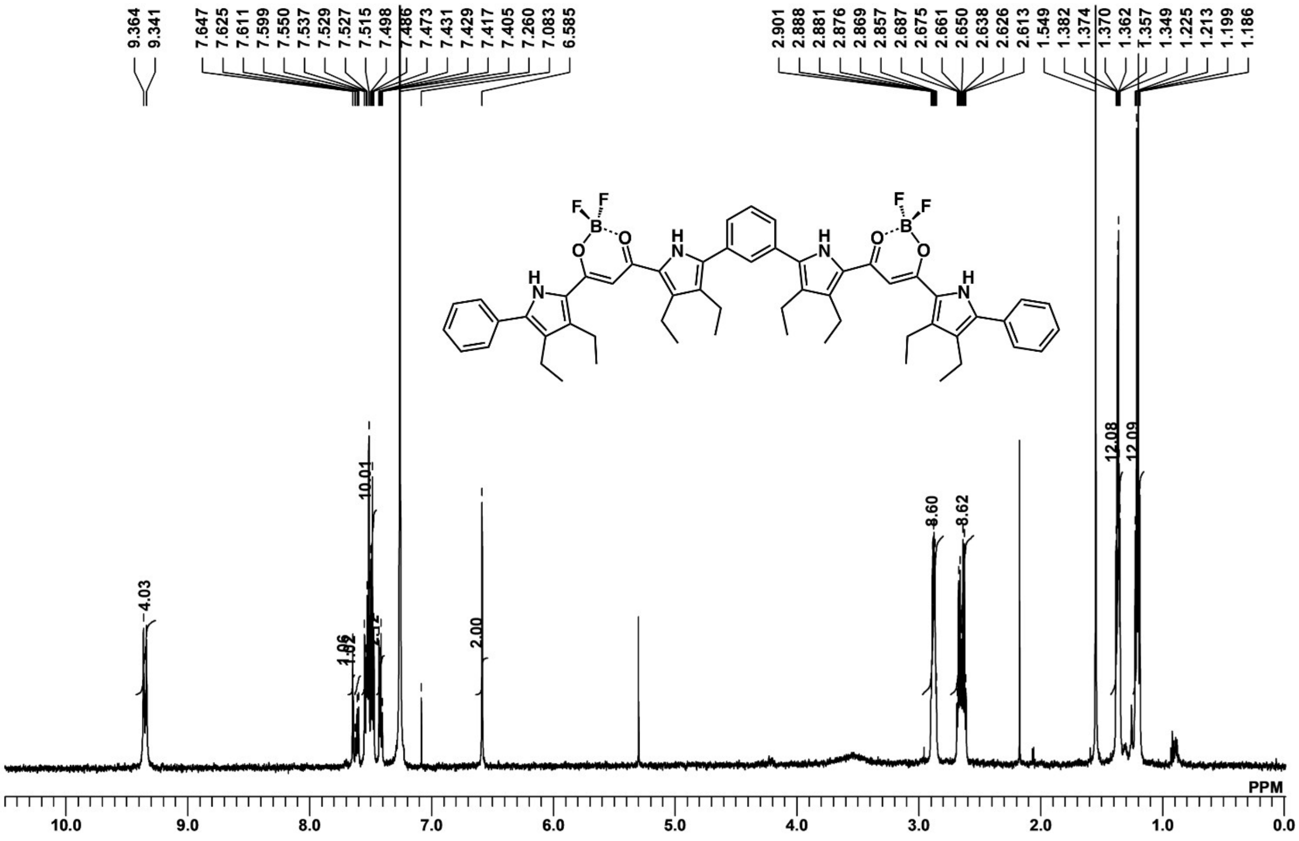


**Figure S26.** $^1$H NMR spectrum of **H** in $CDCl_3$.

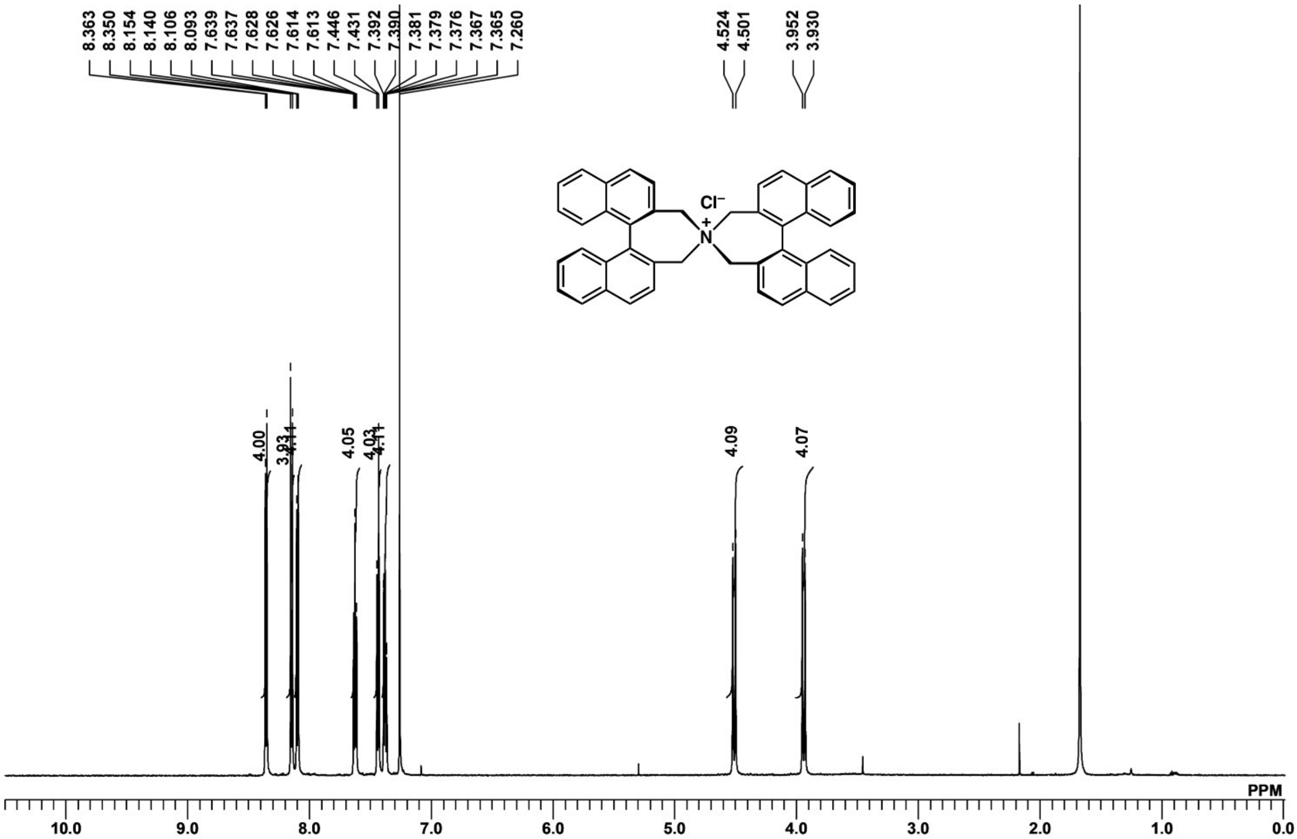


**Figure S27.** $^1$H NMR spectrum of **RR**•Cl in $CDCl_3$.

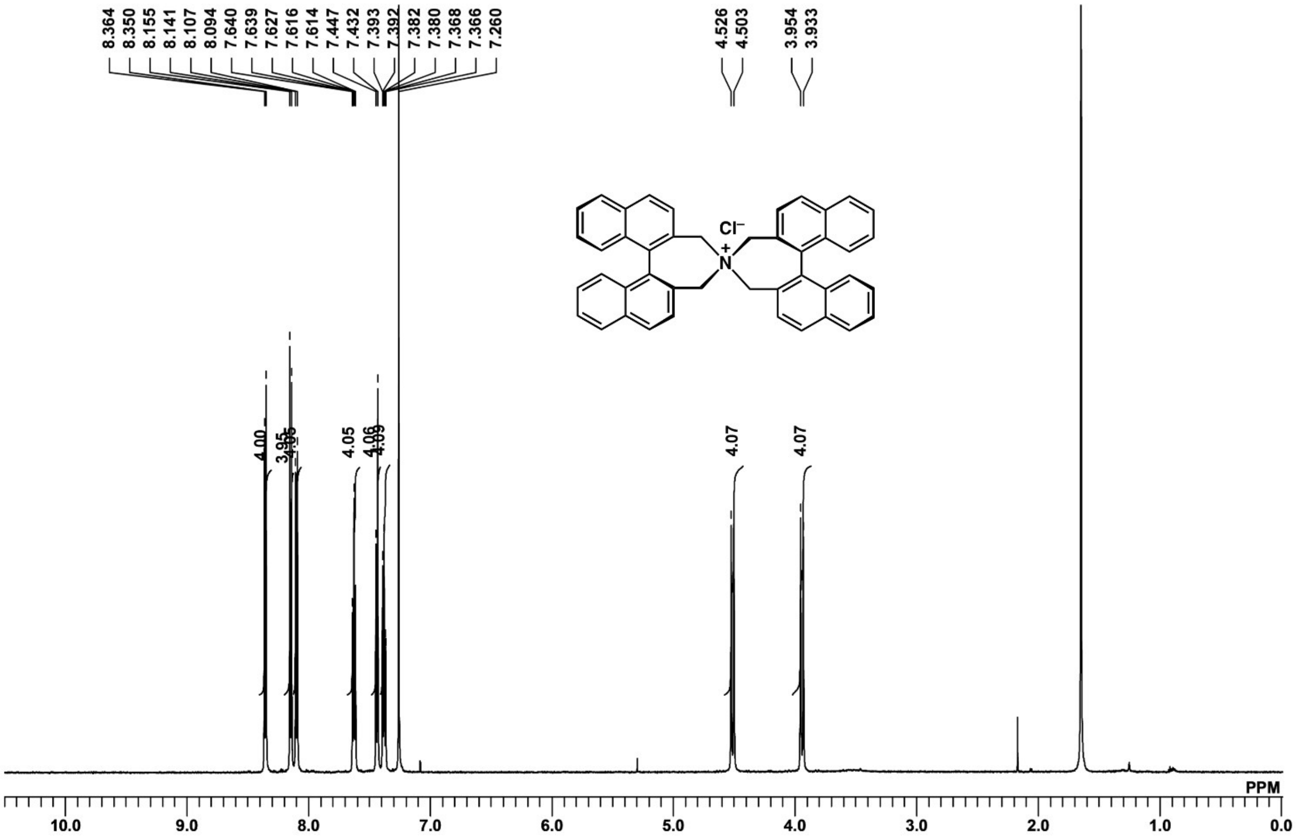


**Figure S28.** $^1$H NMR spectrum of **SS**•Cl in $CDCl_3$.

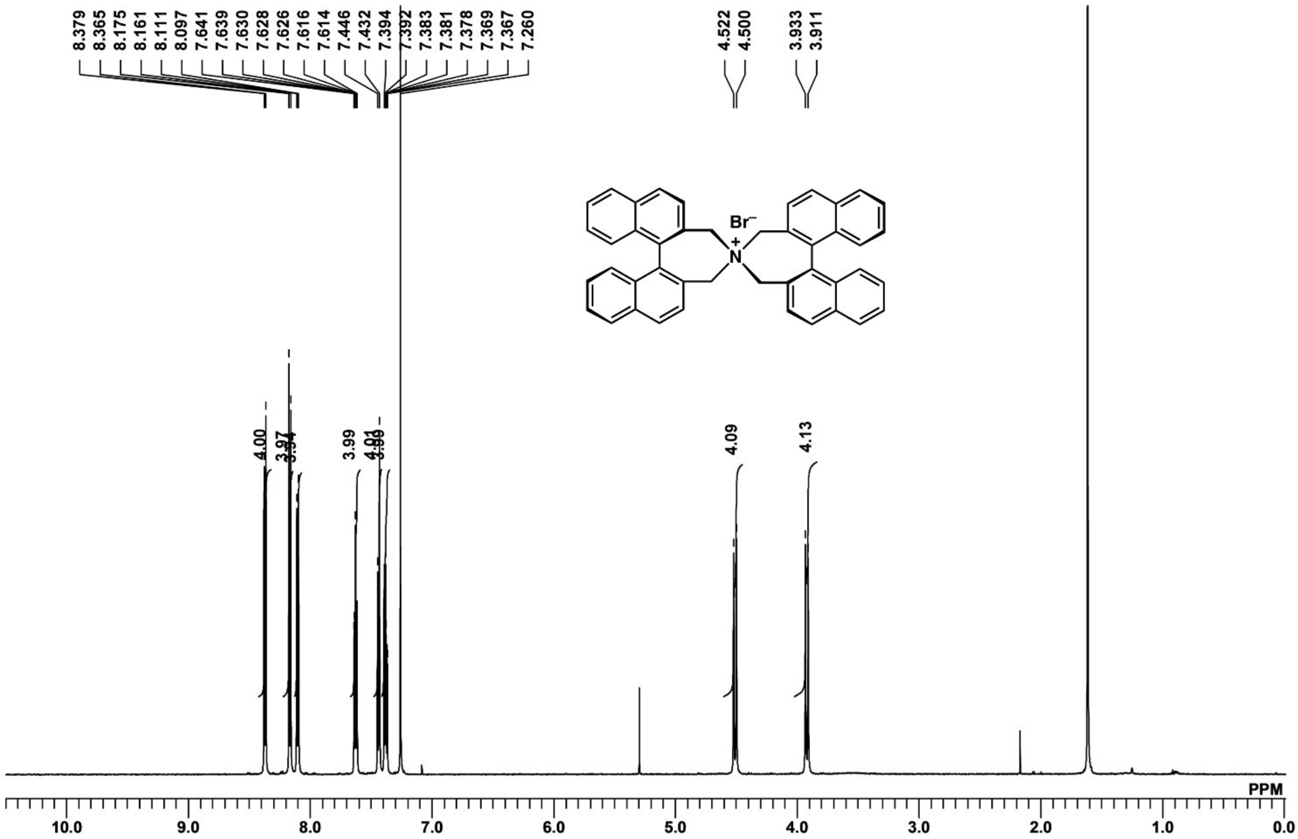


**Figure S29.** $^{1}$H NMR spectrum of **RR**•Br in $CDCl_3$.

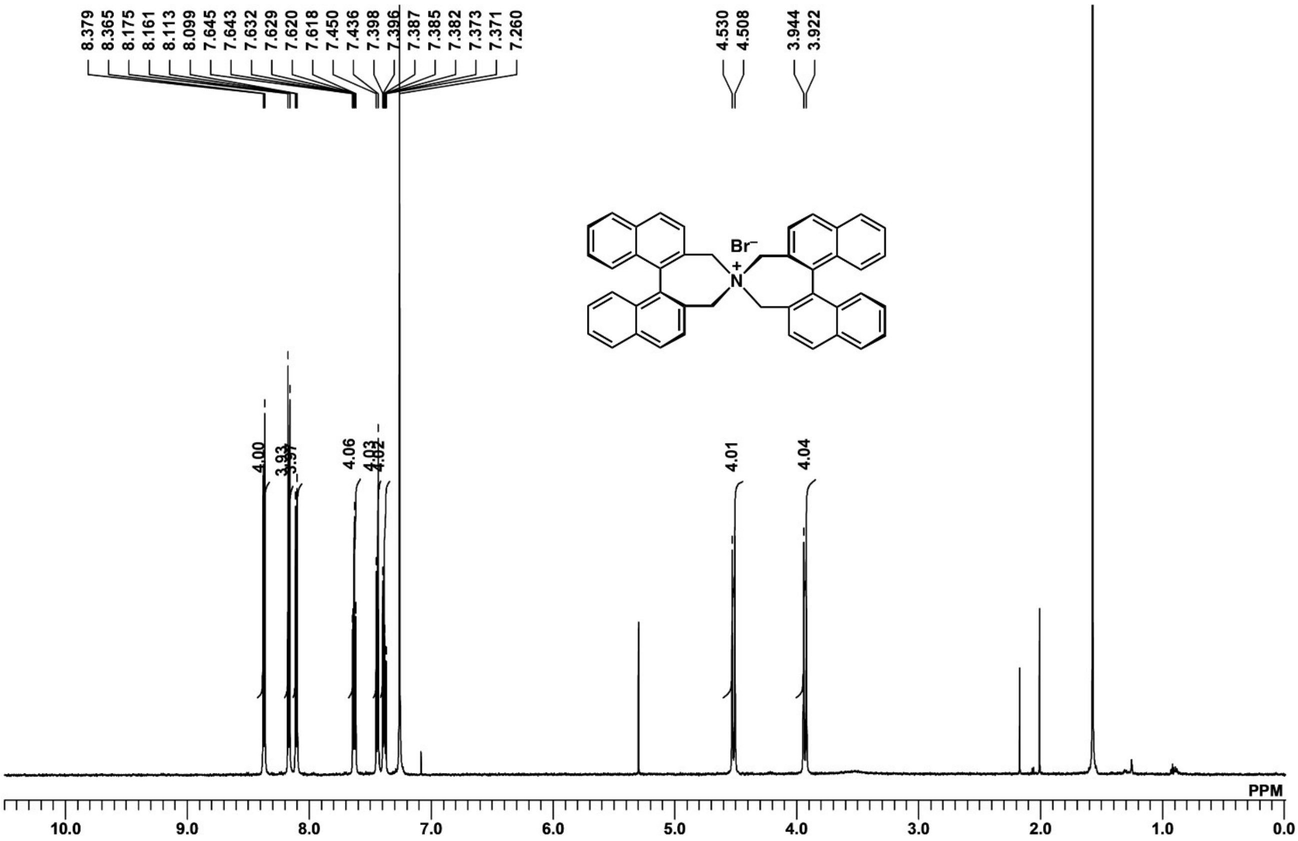


**Figure S30.** $^{1}$H NMR spectrum of **SS**•Br in $CDCl_3$.
